# Genetic and Antigenic Characterization of an Influenza A(H3N2) Outbreak in Cambodia and the Greater Mekong Subregion during the COVID-19 Pandemic, 2020

**DOI:** 10.1128/JVI.01267-21

**Published:** 2021-11-23

**Authors:** Jurre Y. Siegers, Vijaykrishna Dhanasekaran, Ruopeng Xie, Yi-Mo Deng, Sarika Patel, Vanra Ieng, Jean Moselen, Heidi Peck, Ammar Aziz, Borann Sarr, Savuth Chin, Seng Heng, Asheena Khalakdina, Michael Kinzer, Darapheak Chau, Philomena Raftery, Veasna Duong, Ly Sovann, Ian G. Barr, Erik A. Karlsson

**Affiliations:** a National Influenza Center of Cambodia, Virology Unit, Institut Pasteur du Cambodge, Phnom Penh, Cambodia; b School of Public Health, LKS Faculty of Medicine, The University of Hong Kong, Hong Kong, China; c HKU-Pasteur Research Pole, School of Public Health, LKS Faculty of Medicine, The University of Hong Kong, Hong Kong, China; d World Health Organization Collaborating Center, Victorian Infectious Diseases Reference Laboratory, Peter Doherty Institute for Infection and Immunity, Melbourne, Australia; e World Health Organization Country Office, Phnom Penh, Cambodia; f U.S. Centers for Disease Control and Prevention, Phnom Penh, Cambodia; g Centers for Disease Control and Prevention, Ministry of Health, Phnom Penh, Cambodia; h National Institute of Public Health, Ministry of Health, Phnom Penh, Cambodia; University of North Carolina at Chapel Hill

**Keywords:** Influenza, Cambodia, A(H3N2), vaccine, outbreak, COVID-19, Laos, Vietnam, influenza

## Abstract

Introduction of non-pharmaceutical interventions to control COVID-19 in early 2020 coincided with a global decrease in active influenza circulation. However, between July and November 2020, an influenza A(H3N2) epidemic occurred in Cambodia and in other neighboring countries in the Greater Mekong Subregion in Southeast Asia. We characterized the genetic and antigenic evolution of A(H3N2) in Cambodia and found that the 2020 epidemic comprised genetically and antigenically similar viruses of Clade3C2a1b/131K/94N, but they were distinct from the WHO recommended influenza A(H3N2) vaccine virus components for 2020–2021 Northern Hemisphere season. Phylogenetic analysis revealed multiple virus migration events between Cambodia and bordering countries, with Laos PDR and Vietnam also reporting similar A(H3N2) epidemics immediately following the Cambodia outbreak: however, there was limited circulation of these viruses elsewhere globally. In February 2021, a virus from the Cambodian outbreak was recommended by WHO as the prototype virus for inclusion in the 2021–2022 Northern Hemisphere influenza vaccine.

**IMPORTANCE** The 2019 coronavirus disease (COVID-19) pandemic has significantly altered the circulation patterns of respiratory diseases worldwide and disrupted continued surveillance in many countries. Introduction of control measures in early 2020 against Severe Acute Respiratory Syndrome Coronavirus-2 (SARS-CoV-2) infection has resulted in a remarkable reduction in the circulation of many respiratory diseases. Influenza activity has remained at historically low levels globally since March 2020, even when increased influenza testing was performed in some countries. Maintenance of the influenza surveillance system in Cambodia in 2020 allowed for the detection and response to an influenza A(H3N2) outbreak in late 2020, resulting in the inclusion of this virus in the 2021–2022 Northern Hemisphere influenza vaccine.

## INTRODUCTION

Seasonal influenza viruses cause significant human morbidity and mortality worldwide (1). Epidemics occur annually in temperate regions and seasonality can vary in tropical/subtropical regions with year‐round circulation in some areas and epidemic intensity varying by year and by region (2, 3). The disease burden of influenza can be ameliorated by prophylactic vaccination (4), and mitigated with antiviral drugs and social interventions such as school closures (5, 6). However, continued global surveillance is necessary to ensure the availability of vaccine strains that best match the circulating seasonal influenza viruses as well as potential pandemic influenza viruses (5, 7). Emerging evidence suggests that the SARS-CoV-2 pandemic has significantly altered the circulation patterns of respiratory diseases worldwide and disrupted their continued surveillance in many countries. Indeed, introduction of control measures in early 2020 against Severe Acute Respiratory Syndrome Coronavirus-2 (SARS-CoV-2) infection has resulted in a remarkable reduction in the circulation of many respiratory diseases (8–10). Influenza activity, for example, has remained at historically low levels globally since March 2020, even when increased influenza testing was performed in some countries (11, 12). Interestingly, the majority of influenza detections in 2020 and in the first part of 2021 have been in Southeast Asia and other tropical regions such as Western Africa (13–15).

The Kingdom of Cambodia (population: 16.5 million) is a tropical country in the Greater Mekong Subregion (GMS) of Southeast Asia, sharing international borders with Thailand, Laos PDR, and Vietnam. Influenza detection in Cambodia normally increases during March–June, and peaks between June and November during rainy season, consistent with influenza circulation patterns in the temperate regions of the Southern Hemisphere, although low level year‐round circulation of influenza is detected in Cambodia (16). Due to a robust and swift response, SARS–CoV-2 was well managed and controlled in Cambodia and other parts of the GMS, and Cambodia detected no widespread community transmission throughout 2020. However, in July 2020, influenza detections began to increase in the Cambodian influenza-like illness (ILI) sentinel surveillance systems reflecting a country-wide outbreak that included clustered cases in closed/semi-closed settings (prisons/pagodas) and spreading into the general community (13). Influenza cases were subsequently detected in Vietnam, Laos PDR, and Thailand (14, 15) by their WHO National Influenza Centers (NICs). Here we describe virological findings from sentinel ILI surveillance in Cambodia and the region. In addition, genetic and antigenic characteristics of influenza viruses detected during the regional outbreaks were assessed to determine the degree of vaccine match with the currently recommended A(H3N2) influenza vaccine and the likelihood of a more global spread in the future.

## RESULTS

### Laboratory detection of influenza in Cambodia, 2020, in relation to stringency.

**FIG 1:**
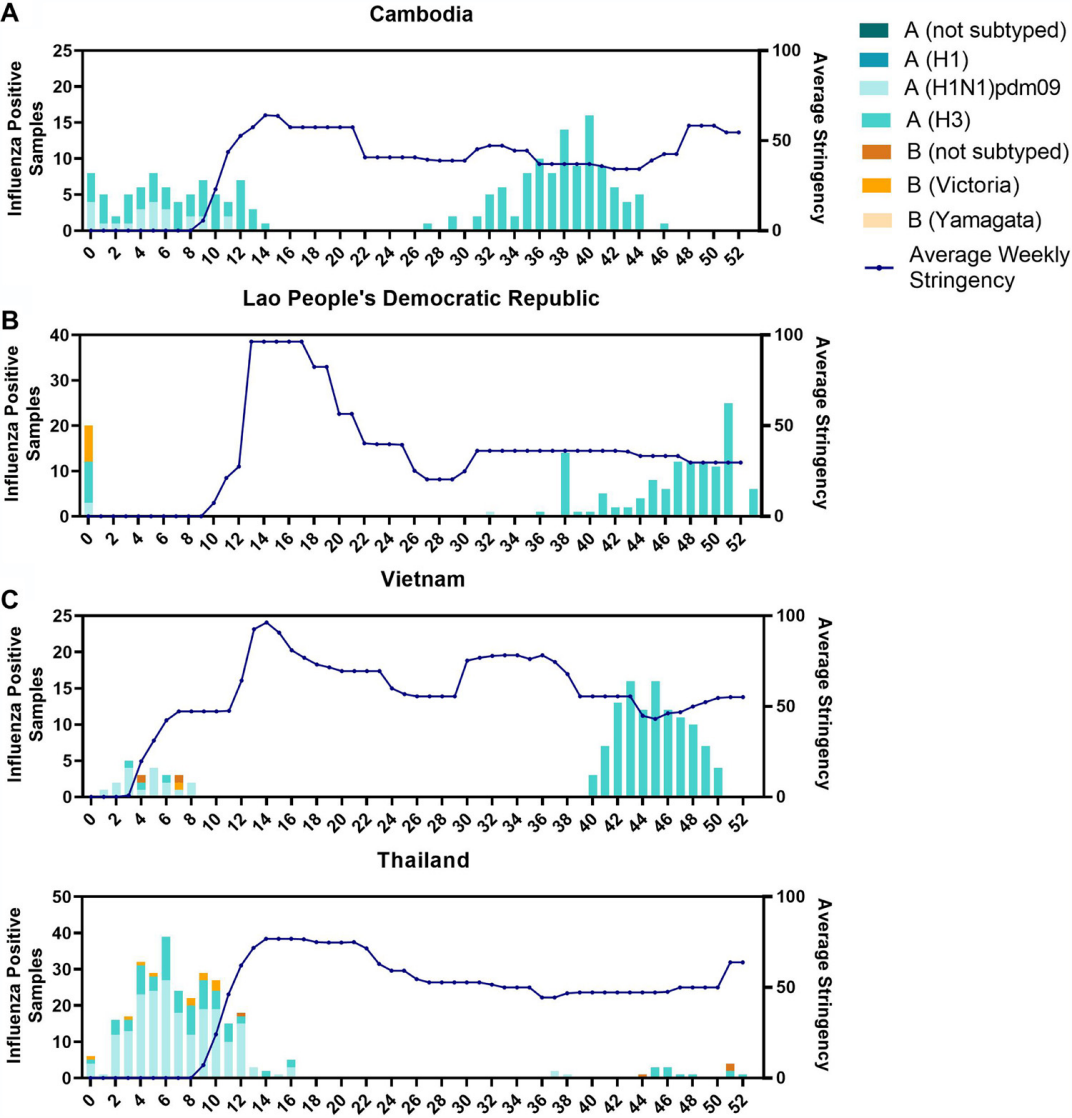
Influenza detection by week in Cambodia, Laos PDR, Thailand, and Vietnam in 2020 compared to pandemic response stringency. Influenza detection in Cambodia (A), Laos PDR (B), Thailand (C), and Vietnam (D) compared to average weekly stringency (19) of public health policies enacted in accordance with the COVID-19 pandemic (blue line).

While influenza virus detection typically increases in Cambodia from April to June (16), no laboratory confirmed cases of influenza were detected in Cambodia between April (week 14) until mid-June (week 24, Fig. 1) 2020. This reduction coincided with the implementation of several COVID-19 public health prevention measures and intervention policies (19) (Fig. 1). However, a significant rise in laboratory confirmed influenza A(H3N2) cases occurred from the end of July 2020 (week 31) until mid-November (week 46). Peak positivity occurred at week 34, with 36% (12/33) of the ILI and SARI samples testing positive for influenza A(H3N2). In total, there were 368 laboratory-confirmed cases of influenza A(H3N2) from 11 outbreaks in 10 provinces between July and November 2020 in Cambodia. Overall positivity rate for laboratory confirmed influenza A(H3N2) in Cambodian ILI samples was 77% (368/475).

### Phylogenetic analysis of recent Cambodian influenza A(H3N2) viruses.

**FIG 2:**
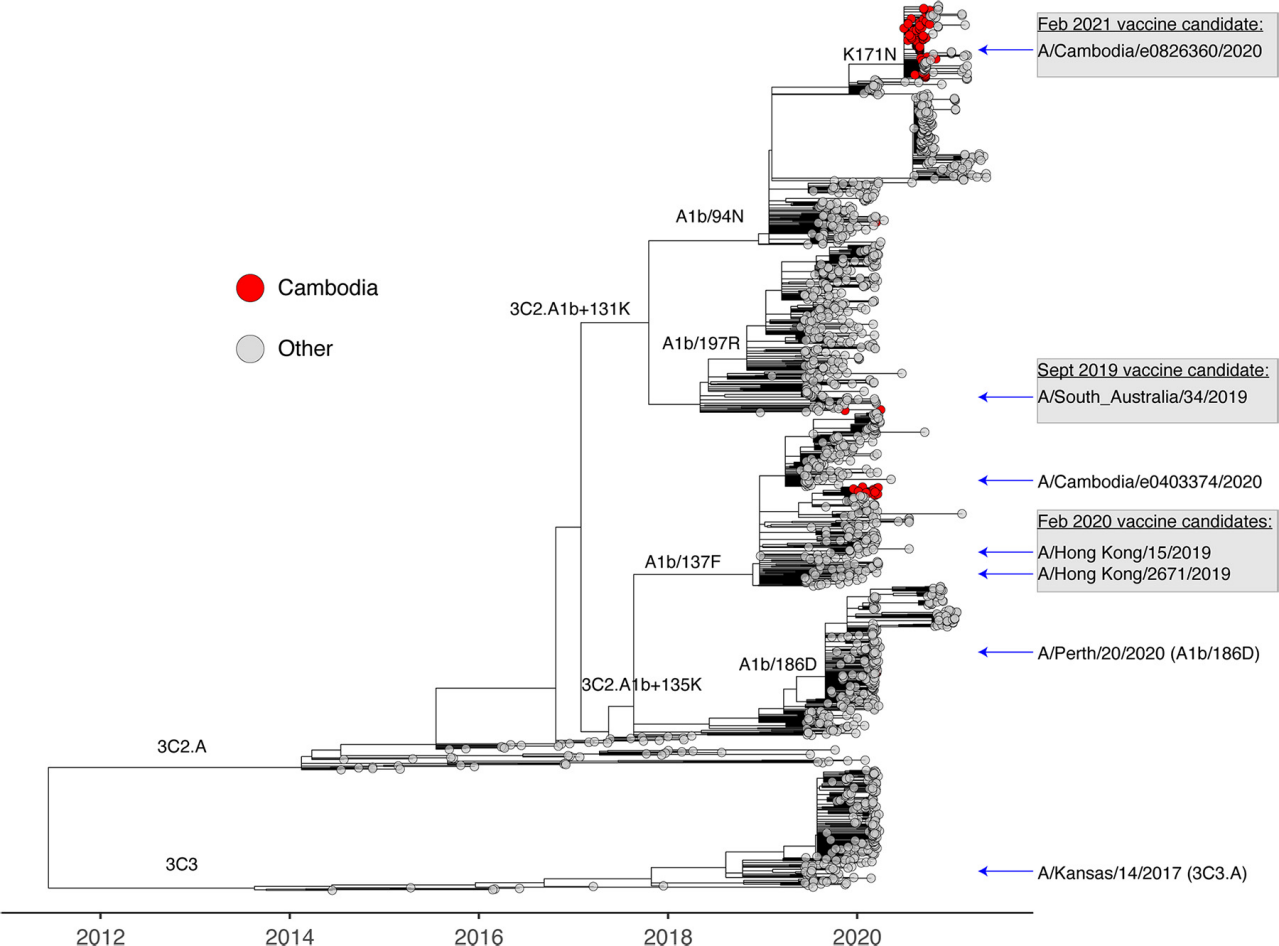
Dated phylogeny of HA gene of A(H3N2) virus showing an apparent global bottleneck in 2020. The analysis was based on 1,387 globally representative samples using the best-fit nucleotide substitution model (GTR+F3+R4). Cambodia viruses are colored in red, and reference strains and recent vaccine strains are highlighted along the tips.

**FIG 3:**
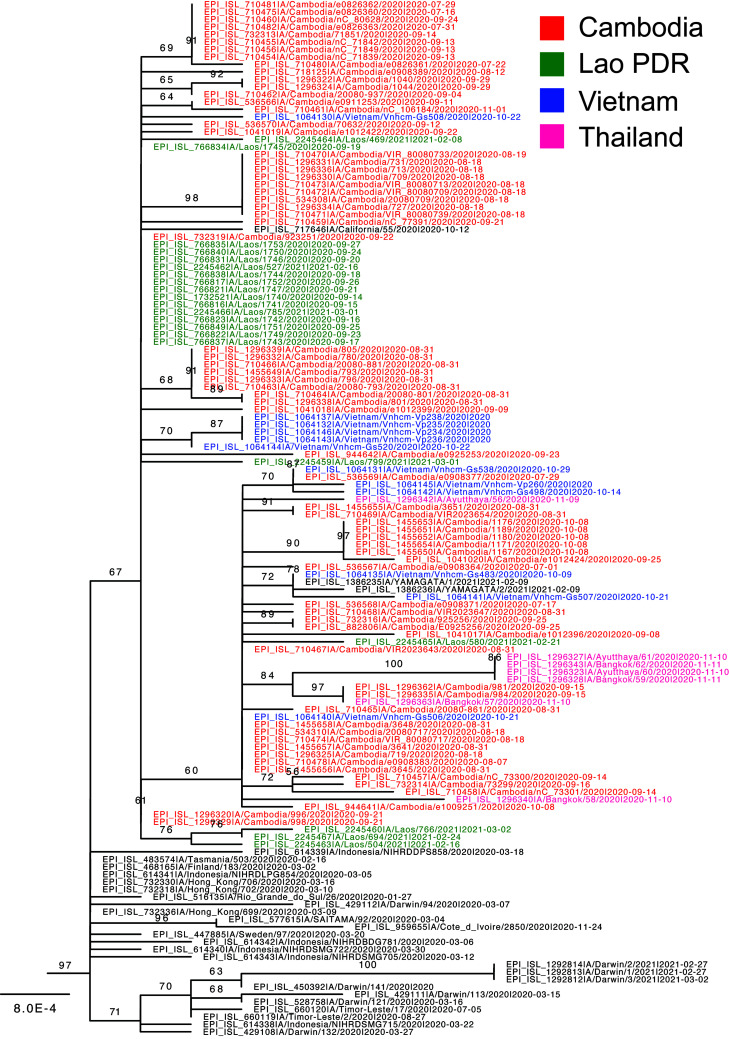
Evolutionary relationships of the A(H3N2) viruses in Southeast Asia, late 2020–2021. Maximum likelihood phylogeny with branch lengths in substitutions per site (scale bar) generated using the best-fit nucleotide substitution model (K3Pu+F). Viruses collected from Southeast Asia from Jun 2020 are colored by country.

Phylogenetic analysis of the HA genes showed that A(H3N2) viruses collected in Cambodia during 2019 until March 2020 were derived from both major A(H3N2) clades circulating prior to COVID-19 emergence, including subclades 3C2.A1b + 135K and 3C2.A1b + 131K, with the majority forming a cluster belonging to 3C2.A1b + 135K (reference strain: A/Cambodia/e0403374/2020) (15, 23). In contrast, all Cambodian A(H3N2) viruses collected during the outbreaks from July 2020 to November 2020 (such as A/Cambodia/e0826360/2020) belonged to 3C2.A1b + 131K clade and were highly similar to each other (Fig. 2). They formed a distinct cluster with viruses collected in neighboring Laos PDR, Vietnam, and Thailand (Fig. 3). This predominantly Southeast Asian A(H3N2) cluster formed a sister group containing samples from South Asia, collected from Bangladesh and India from August 2020, and appear to be circulating in southeast Asia and the Australia during early 2020 (reference: A/Tasmania/503/2020) (15, 23).

Maximum likelihood phylogenetic analysis showed that viruses similar to those detected in Cambodia were also detected in a similar time period in all three bordering countries including Laos PDR, Vietnam, and Thailand, and that cross-border transmission may have occurred on multiple occasions. Samples collected from Vietnam and Thailand formed five and three distinct clusters, respectively, that were derived from viruses collected in Cambodia, indicating transmission has occurred on multiple events along these borders; however, the direction of migration between Cambodia and Laos PDR could not be confirmed as their HA genes were highly identical (Fig. 3). Detection of viruses in Vietnam and Laos PDR occurred rapidly after detection in Cambodia from August to November with additional analysis of the WHO Flunet system (Fig. 1). Furthermore, while no additional sequences were reported from Southeast Asia since December 2020, cases reported from Laos PDR during February to March in 2021 were similar to the Cambodian outbreak based on their HA sequences (Fig. 3), showing that some lineages emerging within the Cambodian outbreak continued to circulate in Laos PDR until early 2021.

**FIG 4:**
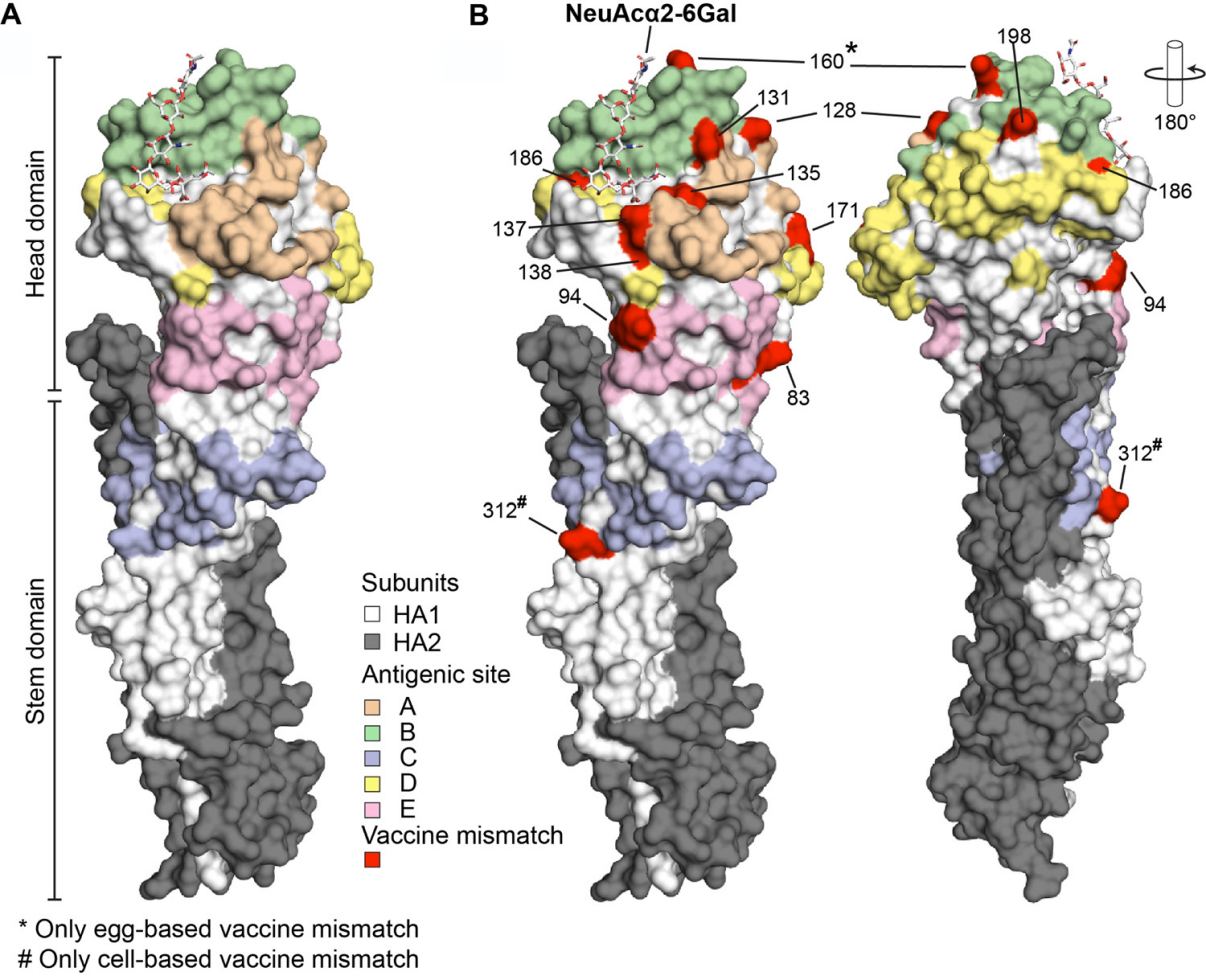
Location of antigenic mismatches between the 2020/2021 H3N2 vaccine virus and recent Cambodian viruses. (A) Influenza HA subunits and antigen sites A–E mapped on the crystal structure of the A/Brisbane/10/2007 (H3N2) influenza virus HA in complex with NeuAcα2-6Gal (PDB: 6AOV). (B) Antigenic mismatches between the 2020/2021 H3N2 vaccine seed strains and recent Cambodian viruses. The 2020/2021 vaccine seed strains included both the egg-based (A/Hong Kong/2671/2019) and cell-based (A/Hong Kong/45/2019) vaccine. Egg -and cell-based specific antigenic mismatches are indicated by * and #, respectively.

In comparison to previously circulating viruses, both the South and Southeast Asian A(H3N2) lineages (3C2.A1b + 131K) contained signature HA amino acid substitutions at K83E, Y94N, F193S, and Y195F, while the Southeast Asian lineage contained additional substitutions at G186S, S198P, and K171N (Fig. 2 and 4). In recognition of these independent outbreaks in South and Southeast Asia with additional HA substitutions, the A/(H3N2) influenza vaccine prototype was updated in February 2021 to include a Cambodian outbreak virus strain (A/Cambodia/e0826360/2020) representative of recent 3C2.A1b + 131K viruses (15).

### Antigenic analysis of 2020 Cambodian influenza A(H3N2) viruses.

Influenza A(H3N2) isolates were successfully generated from Cambodian clinical samples using MDCK-SIAT-1 cells, and all virus isolates obtained agglutinated guinea pig RBC well, with samples collected early in 2020 generating isolates with hemagglutination titers of generally 16–64 and late collection 2020 isolates generally having titers of 8–32 (measured in the absence of oseltamivir carboxylate). Antigenic analysis by HI was performed on these early and late 2020 Cambodian A(H3N2) virus isolates against a panel of reference viruses and ferret postinfection antisera and a pool of postvaccination human sera. Table 1 shows the results with the recent Cambodian viruses giving similar reactivity to A/Tasmania/503/2020-like viruses and viruses collected from Timor-L’este in July–August 2020, (which also grouped in the same genetic HA clade; see Fig. 3) and were distinct from earlier 2020 Cambodian viruses (such as A/Cambodia/e0403374/2020 collected on 8 March 2020; Fig. 2). Reduced reactivity was seen to these Cambodian viruses with other ferret antisera generated to an A/Hong Kong/2671/2019-like virus (A/Darwin/726/2019), A/South Australia/34/2019 (the 2020 Southern hemisphere recommended A(H3N2) vaccine virus), and the previous Northern hemisphere 2019–2020 recommended vaccine virus A/Kansas/14/2017, which showed 2–8-fold reductions with ferret antisera raised to this virus (data not shown). Reduced reactivity was also seen with the 2020 postvaccination Australian human serum pool. Together this indicates that the late 2020 Cambodian viruses were antigenically distinct from the previous two A(H3N2) vaccine viruses and therefore may escape prior immunity generated by infection or vaccination with these previous A(H3N2) viruses.

**TABLE 1:** Antigenic analysis of 2020 Cambodian influenza A(H3N2) viruses by hemagglutination inhibition assay^*a*^

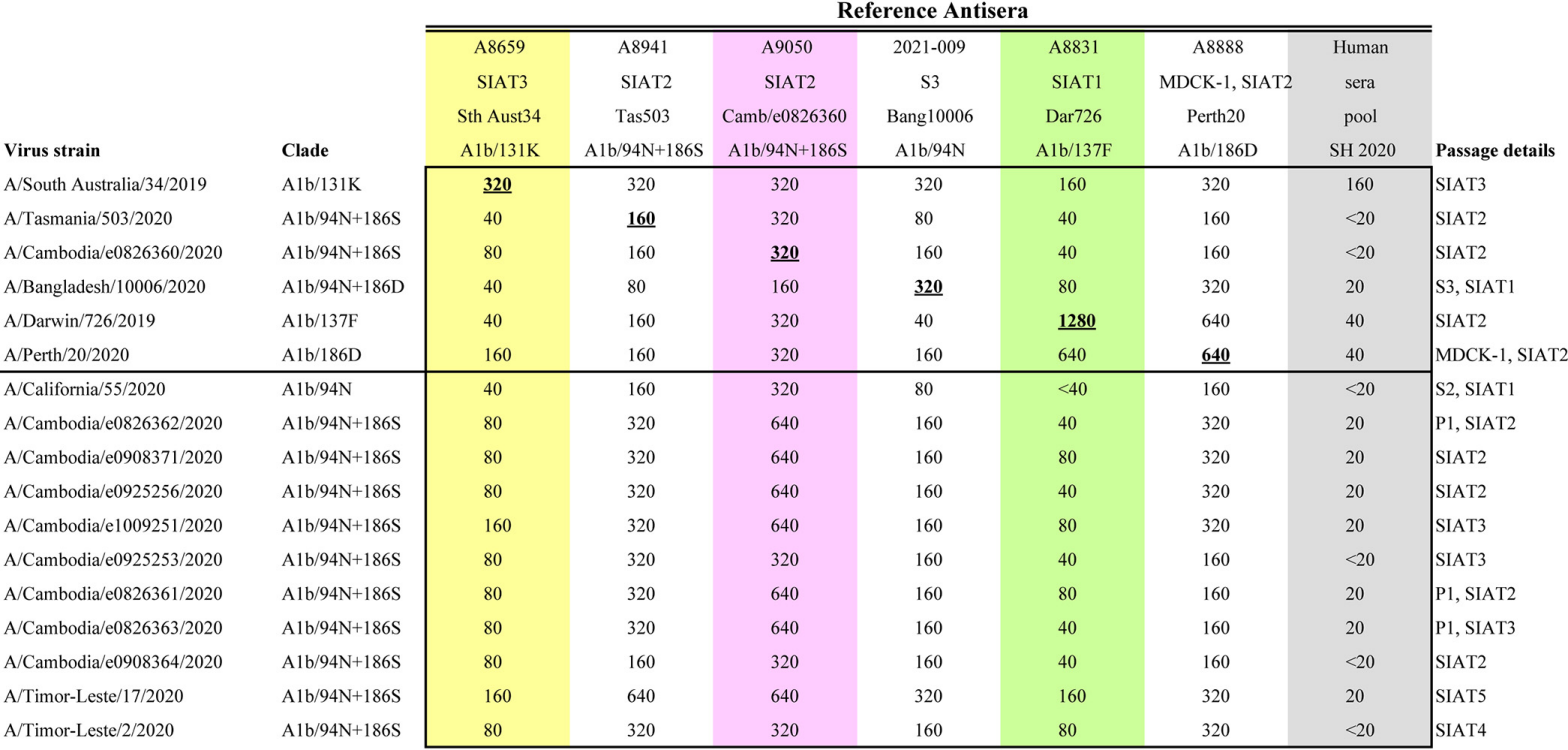

a Yellow highlight indicates sera raised against the 2019 vaccine recommended strain. The purple indicates sera raised against the 2021 vaccine candidate. Green indicates sera raised against A/Darwin/726/2019, which is the cell-based equivalent of the 2021 WHO recommended Southern Hemisphere vaccine strain, A/Hong Kong/2671/2019. Gray indicates the human sera pool of individuals vaccinated with a quadrivalent vaccine containing a A/South Australia/34/2019-like A(H3N2) virus.

### Visualization of antigenic mismatches on the H3 structure.

Neutralizing antibodies predominantly recognize antigenic sites on the globular head of the HA (epitopes A–E, Fig. 4A) (33), and most major antigenic changes of H3N2 viruses between 1968 and 2013 are accredited to mutations in antigenic site B (34, 35). However, mutations in other sites can also contribute to antigenic escape/evolution of the virus. To complement the antigenic distance measures by the HI assay, we visualized the antigenic mismatches in dominant epitopes between the vaccine strains and Cambodian 2020 viruses on the crystal structure of the A/Brisbane/10/2007 A(H3N2) (Fig. 4). With the 2020/2021 Northern and Southern hemisphere egg-based vaccine strain A/Hong Kong/45/2019 (H3N2), a total of 11 mismatches were observed. A total of 4, 3, 1, 1, and 2 mismatches were observed in antigenic sites A, B, C, D, and E, respectively (Fig. 4B; Table 2). With the 2020/2021 Northern and Southern hemisphere cell-based vaccine strain A/Hong Kong/2671/2019 (H3N2), a total of 11 mismatches were observed. A total of 4, 4, 0, 1, and 2 mismatches were observed in antigenic sites A, B, C, D, and E, respectively (Fig. 4B; Table 2). Collectively, this supports the antigenic data that vaccines based on A/Hong Kong/2671/2019-like viruses may have reduced efficacy against currently circulating Cambodian H3N2 viruses and is consistent with the need to update the annual vaccine to account for these changes.

**TABLE 2:** Comparison of amino acid differences in major antigenic sites between Cambodian A(H3N2) strains and recommended vaccine composition strains for 2020/2021.

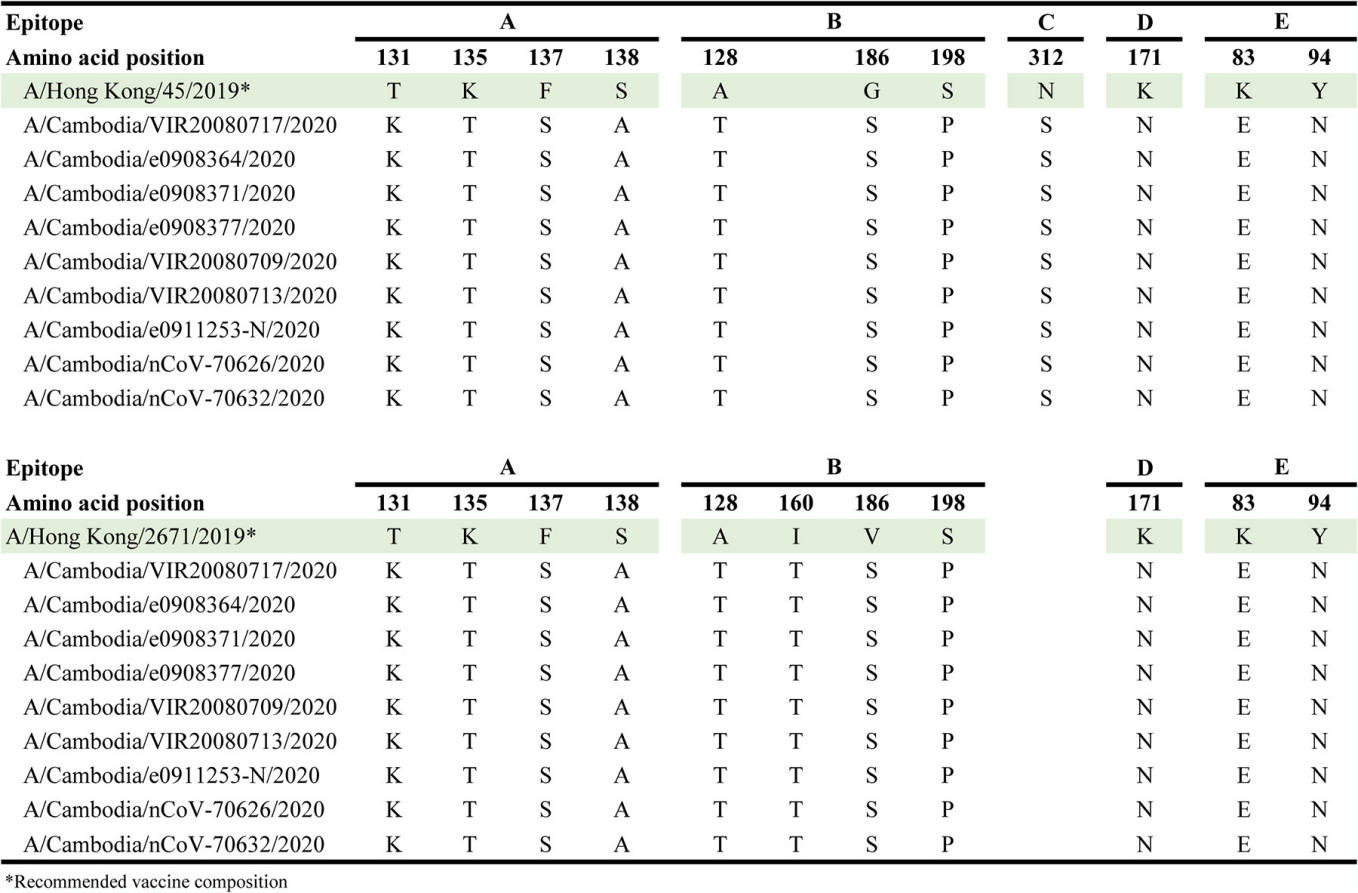

## DISCUSSION

Following the WHO declaration of COVID-19 as a Public Health Emergency of International Concern (36), and COVID-19 as an international pandemic (37), numerous non-pharmaceutical intervention (NPI) strategies were implemented globally to prevent, detect and contain viral spread (38), including closing borders or suspending international flights, entry quarantine for travelers, physical distancing, hand washing, mask wearing, and mass testing. It is apparent that these swift and more stringent measures taken to mitigate the spread of SARS-CoV-2 have also affected the global circulation of influenza viruses (11), with implementation of these prevention measures directly coinciding with a global reduction of influenza in most countries. However, while global detection was low, influenza was present in a small number of countries during the normal winter periods in the Southern hemisphere, mainly in the tropical South/Southeast Asia (14) and West Africa transmission zones (15). In Cambodia, a number of NPIs were introduced by the Cambodian government to break COVID-19 transmission chains. However, as stringency in policies decreased (19), some influenza was detected in sentinel systems in late June 2020. It is unknown whether the virus may have been circulating at low levels for some time before detection or when the virus was introduced into the country. Interestingly, outbreaks of A(H3N2) were also detected in other places sharing borders with Cambodia, namely, Vietnam and Laos PDR, but only a few sporadic detections were seen in Thailand. This may indicate the virus was circulating in the region through travel across land borders or other unknown transmission routes.

Cambodian A(H3N2) viruses from late 2020 were both genetically and antigenically distinct from viruses circulating earlier in the year in Cambodia, and different from the A(H3N2) viruses that circulated in many parts of the world in 2020. Therefore, low level immunity against these viruses may pose an increased risk of them producing outbreaks and epidemics in the Northern hemisphere in 2021–2022 and in the 2022 Southern hemisphere seasons if they continue to circulate. Indeed, previous studies have shown that A(H3N2) viruses are regularly re-seeded from East and Southeast Asia into other parts of the world (39). Given the finding that these recent Cambodian viruses appear to be antigenically distinct from the current A(H3N2) vaccine viruses, coupled with the lack of influenza virus circulation in many other countries, the number of people at risk from infection with A(H3N2) may increase when (inter)national travel and other social restrictions are eased. Due to the risk from this virus, Cambodian A(H3N2)-like viruses have been recommended by the WHO as a vaccine prototype strain for the Northern Hemisphere 2021/2022 influenza season (15).

Overall, these data highlight that despite the ongoing global COVID-19 pandemic, influenza virus outbreaks continue to occur, such as those seen in Cambodia, Vietnam, and Laos PDR. While global influenza circulation is still currently low up to mid-2021, including in Cambodia, outbreaks can and will occur, especially in closed, high-contact settings such as prisons and at religious gatherings, where enforcement and maintenance of NPIs are more challenging. It is unknown when and where influenza will return in force following the COVID-19 pandemic, especially as international travel reopens/resumes. Maintenance of global influenza surveillance is vital not only for the continued detection of influenza, but also for other respiratory diseases including COVID-19. Rapid investigation of outbreaks and differential diagnosis is critical to determine the etiology of the outbreak not only to rule out SARS-CoV-2, but to ensure the appropriate public health actions. Timely testing, sequencing, and sharing of respiratory virus data (including from influenza viruses) are critically important for all countries, especially in the context of the lack of global influenza samples that have been available since mid-2020 for vaccine development, challenging the continued need for well-matched seasonal influenza vaccines.

## MATERIALS AND METHODS

### Ethics.

The Cambodian ILI and Severe Acute Respiratory Illness (SARI) surveillance systems are part of the WHO Global Influenza Surveillance and Response System and are a public health activity managed by the Ministry of Health (MoH) and Cambodian Centers for Disease Control and Prevention that has a standing authorization from the National Ethics Committee for Human Research. Samples collected under the Cambodian COVID-19 response are permitted to be further analyzed for pathogens of public health concern in the context of influenza circulating throughout the country. Samples and patient information were anonymized for the purposes of this surveillance.

### Influenza detection and laboratory investigations.

Influenza in Cambodia was tested using RT-PCR in all sentinel surveillance samples from November 2019 to Dec 2020 that were suspected of respiratory disease outbreak (COVID-19 and ILI), and those referred from symptomatic suspect COVID-19 cases (16, 17), according to recommendations for testing under the Global Influenza Surveillance and Response System guidelines (18). Total influenza data reported from Cambodia, Laos PDR, Vietnam, and Thailand from January to December, 2020 were downloaded from FluNET (https://www.who.int/tools/flunet).

### COVID-19: stringency index.

Weekly calculated stringency indices of COVID-19 response for each country were obtained from the Oxford COVID-19 Government Response Tracker (19).

### Sequencing.

Cambodian influenza A whole genomes were amplified using Uni12/Inf-1 and Uni13/Inf-1 (20) with SSIII One-step RT-PCR with Platinum *Taq* High Fidelity kit (Thermo Fisher, Australia), and 14 μl of RNA from original specimens or 8 μl of RNA from cultured virus were used for the reaction, following manufacturer’s instructions. After amplification, amplicons were quantified on a TapeStation 4200 using D5000 ScreenTape (Agilent, Australia). Amplicons were normalized with nuclease free water to contain 200 ng of target DNA in 30 μl volume and used for NGS library construction using Nextera DNA Flex Library Prep kit and IDT for Illumina Nextera DNA Unique Dual Indexes Sets A-D (Illumina, Australia), followed by library clean up and pooling according to the manufacturer’s instructions. The pooled library was quantified on the TapeStation 4200 with Tapescreen HSD1000 and Qubit 1X dsDNA HS assay kit on the Qubit 4 (Thermo Fisher, Australia). The pooled library was then diluted to 150pM in elution buffer supplied in the library prep kit and was loaded into the iSeq100 i1 v2 (300-cycle) cartridge containing the flow cell. The sequencing parameters for the iSeq100 system were set at pair end reads, 2 × 151 bp read length with adaptor trimming on. After the NGS run, fastq data were retrieved and analyzed using the IRMA pipeline (21) to generate consensus sequences for the whole genome sequences.

### Phylogenetic analysis.

The hemagglutinin (HA) sequences of Cambodian A(H3N2) viruses were analyzed with sequences from the Global Initiative on Sharing All Influenza Data (GISAID) (22). The GISAID data were selected based on criteria used by Nextstrain (23), in which a greater frequency of recent samples were sampled along with key reference sequences. We included all available HA sequences available since March 2020. Following multiple sequence alignment using Muscle v.3.8 (24), phylogenetic relationships were inferred using the maximum-likelihood (ML) method in IQTREE v.2.0 (25) using the best-fit nucleotide substitution model and dated using Least Squares Dating (LSD) method (26). Data quality was investigated using a root-to-tip regression of the ML tree and collection dates in TempEst v.1.5 (27), and branch supports were estimated using the ultrafast bootstrap method in IQTREE v.2.0 (28).

### Hemagglutination inhibition assay.

Antigenic properties were determined using the hemagglutination inhibition (HI) assay (29). Influenza virus isolates were generated by inoculation of influenza positive respiratory samples into MDCK-SIAT-1 cells (30) and stored at −80°C until assayed. Reference ferret antisera were obtained from ferrets 14 days after they had been infected with a range of human A(H3N2) influenza viruses. A pool of human sera obtained from post-vaccinated (4 weeks after vaccination) recipients of the 2020 Australian quadrivalent influenza (egg based) vaccine (that contained an A/South Australia/34/2019-like A(H3N2) virus) was also included in the HI. All of these antisera were pre-treated with receptor-destroying enzyme (Denka Seiken, Tokyo, Japan) to remove any nonspecific binding. HI assays were performed in the presence of 20 nM oseltamivir carboxylate (OC) (kindly provided by Roche AG (Basel, Switzerland)) to reduce nonspecific binding of the NA protein (31). Two-fold serial dilutions were made on the treated serum in phosphate-buffered saline in 96-well U-bottom microtiter plates. All viruses were adjusted to 4 hemagglutination units per 25 μl, and this virus suspension was added to each of the 96 wells before incubating for 30 min at room temperature. After this, 25 μl of freshly prepared 1.0% (vol/vol) guinea pig red blood cells (RBCs) were added to each well and the plates were incubated for a further 45 min at room temperature. Plates were read and HI titers were obtained from the reciprocal of the highest serum dilution that contained non-agglutinated RBCs. All animal work was approved by the University of Melbourne Animal Ethics Committee.

### Structural modeling.

The X-ray crystal structure of the influenza A virus hemagglutinin of A/Brisbane/10/2007 (H3N2) in complex with NeuAcα2-6Gal (PDB: 6AOV) (32) was used to map the locations of the vaccine strain mismatches using PyMOL Molecular Graphics System, Version 2.3 Schrödinger, LLC.

### Data availability.

The newly determined sequences are available in GISAID (https://www.gisaid.org/) under accession numbers EPI_ISL_1041017-20, 410161-62, 410164, 514677-78, 514680-83, 514689-90, 514701-4, 514708, 514712-3, 710454-482, 718125, and 944641-2.

## References

[1] Iuliano AD, Roguski KM, Chang HH, Muscatello DJ, Palekar R, Tempia S, Cohen C, Gran JM, Schanzer D, Cowling BJ, Wu P, Kyncl J, Ang LW, Park M, Redlberger-Fritz M, Yu H, Espenhain L, Krishnan A, Emukule G, van Asten L, Pereira da Silva S, Aungkulanon S, Buchholz U, Widdowson M-A, Bresee JS, Azziz-Baumgartner E, Cheng P-Y, Dawood F, Foppa I, Olsen S, Haber M, Jeffers C, MacIntyre CR, Newall AT, Wood JG, Kundi M, Popow-Kraupp T, Ahmed M, Rahman M, Marinho F, Sotomayor Proschle CV, Vergara Mallegas N, Luzhao F, Sa L, Barbosa-Ramírez J, Sanchez DM, Gomez LA, Vargas XB, Acosta Herrera a, Llanés MJ, et al. 2018. Estimates of global seasonal influenza-associated respiratory mortality: a modelling study. Lancet 391:1285–1300. 10.1016/S0140-6736(17)33293-2. 29248255 PMC5935243

[2] Tamerius JD, Shaman J, Alonso WJ, Alonso WJ, Bloom-Feshbach K, Uejio CK, Comrie A, Viboud C. 2013. Environmental predictors of seasonal influenza epidemics across temperate and tropical climates. PLoS Pathog 9:e1003194. 10.1371/journal.ppat.1003194. 23505366 PMC3591336

[3] Viboud C, Alonso WJ, Simonsen L. 2006. Influenza in tropical regions. PLoS Med 3:e89. 10.1371/journal.pmed.0030089. 16509764 PMC1391975

[4] Yamayoshi S, Kawaoka Y. 2019. Current and future influenza vaccines. Nat Med 25:212–220. 10.1038/s41591-018-0340-z. 30692696 PMC12973209

[5] Duwe S. 2017. Influenza viruses—antiviral therapy and resistance. GMS Infect Dis 5:Doc04. 30671326 10.3205/id000030 PMC6301739

[6] Ryu S, Ali ST, Cowling BJ, Lau EHY. 2020. Effects of school holidays on seasonal influenza in South Korea, 2014–2016. J Infect Dis 222:832–835. 10.1093/infdis/jiaa179. 32277239 PMC7399705

[7] Hay AJ, McCauley JW. 2018. The WHO global influenza surveillance and response system (GISRS)—a future perspective. Influenza Other Respir Viruses 12:551–557. 10.1111/irv.12565. 29722140 PMC6086842

[8] Perez-Lopez A, Hasan M, Iqbal M, Janahi M, Roscoe D, Tang P. 2020. Dramatic decrease of laboratory-confirmed influenza A after school closure in response to COVID-19. Pediatr Pulmonol 55:2233–2234. 10.1002/ppul.24933. 32598576 PMC7361779

[9] Sullivan SG, Carlson S, Cheng AC, Chilver MBN, Dwyer DE, Irwin M, Kok J, Macartney K, MacLachlan J, Minney-Smith C, Smith D, Stocks N, Taylor J, Barr IG. 2020. Where has all the influenza gone? The impact of COVID-19 on the circulation of influenza and other respiratory viruses, Australia, March to September 2020. Euro Surveill 25(47):2001847. 10.2807/1560-7917.ES.2020.25.47.2001847. PMC7693168 33243355

[10] Yeoh DK, Foley DA, Minney-Smith CA, Martin AC, Mace AO, Sikazwe CT, Le H, Levy A, Blyth CC, Moore HC. 2020. The impact of COVID-19 public health measures on detections of influenza and respiratory syncytial virus in children during the 2020 Australian winter. Clin Infect Dis 72(12):2199–2202. 10.1093/cid/ciaa1475. PMC7543326 32986804

[11] Olsen SJ, Azziz-Baumgartner E, Budd AP, Brammer L, Sullivan S, Pineda RF, Cohen C, Fry AM. 2020. Decreased influenza activity during the COVID-19 pandemic—United States, Australia, Chile, and South Africa, 2020. MMWR Morb Mortal Wkly Rep 69:1305–1309. 10.15585/mmwr.mm6937a6. 32941415 PMC7498167

[12] Huang QS, Wood T, Jelley L, Jennings T, Jefferies S, Daniells K, et al. 2020. Impact of the COVID-19 nonpharmaceutical interventions on influenza and other respiratory viral infections in New Zealand. medRxiv 10.1101/2020.11.11.20228692. PMC7881137 33579926

[13] Sovann LY, Sar B, Kab V, Yann S, Kinzer M, Raftery P, Albalak R, Patel S, Hay PL, Seng H, Um S, Chin S, Chau D, Khalakdina A, Karlsson E, Olsen SJ, Mott JA. 2021. An influenza A (H3N2) virus outbreak in the Kingdom of Cambodia during the COVID-19 pandemic of 2020. Int J Infect Dis 103:352–357. 10.1016/j.ijid.2020.11.178. 33249287 PMC10290288

[14] Mott JA, Fry AM, Kondor R, Wentworth DE, Olsen SJ. 2021. Re-emergence of influenza virus circulation during 2020 in parts of tropical Asia: Implications for other countries. Influenza Other Respir Viruses 15:415–418. 10.1111/irv.12844. 33566441 PMC8051733

[15] World Health Organization. 2021. Recommended composition of influenza virus vaccines for use in the 2021–2022 northern hemisphere influenza season. https://www.who.int/influenza/vaccines/virus/recommendations/202102_recommendation.pdf. Accessed April 2001.

[16] Horwood PF, Karlsson EA, Horm SV, Ly S, Heng S, Chin S, Darapheak C, Saunders D, Chanthap L, Rith S, Y P, Chea KL, Sar B, Parry A, Ieng V, Tsuyouka R, Deng Y-M, Hurt AC, Barr IG, Komadina N, Buchy P, Dussart P. 2019. Circulation and characterization of seasonal influenza viruses in Cambodia, 2012–2015. Influenza Other Respir Viruses 13:465–476. 10.1111/irv.12647. 31251478 PMC6692578

[17] Corman VM, Landt O, Kaiser M, Molenkamp R, Meijer A, Chu DK, Bleicker T, Brünink S, Schneider J, Schmidt ML, Mulders DG, Haagmans BL, van der Veer B, van den Brink S, Wijsman L, Goderski G, Romette J-L, Ellis J, Zambon M, Peiris M, Goossens H, Reusken C, Koopmans MP, Drosten C. 2020. Detection of 2019 novel coronavirus (2019-nCoV) by real-time RT-PCR. Eurosurveillance 25:2000045. 10.2807/1560-7917.ES.2020.25.3.2000045. PMC6988269 31992387

[18] World Health Organization. Maintaining surveillance of influenza and monitoring SARS-CoV-2 – adapting Global Influenza surveillance and Response System (GISRS) and sentinel systems during the COVID-19 pandemic. 2020. https://www.who.int/publications/i/item/maintaining-surveillance-of-influenza-and-monitoring-sars-cov-2-adapting-global-influenza-surveillance-and-response-system-(gisrs)-and-sentinel-systems-during-the-covid-19-pandemic. Accessed April 2001.

[19] Hale T, Angrist N, Goldszmidt R, Kira B, Petherick A, Phillips T, Webster S, Cameron-Blake E, Hallas L, Majumdar S, Tatlow H. 2021. A global panel database of pandemic policies (Oxford COVID-19 Government Response Tracker). Nat Hum Behav 5:529–538. 10.1038/s41562-021-01079-8. 33686204

[20] Zhou B, Donnelly ME, Scholes DT, St George K, Hatta M, Kawaoka Y, Wentworth DE. 2009. Single-reaction genomic amplification accelerates sequencing and vaccine production for classical and Swine origin human influenza A viruses. J Virol 83:10309–10313. 10.1128/JVI.01109-09. 19605485 PMC2748056

[21] Shepard SS, Meno S, Bahl J, Wilson MM, Barnes J, Neuhaus E. 2016. Viral deep sequencing needs an adaptive approach: IRMA, the iterative refinement meta-assembler. BMC Genomics 17:708. 10.1186/s12864-016-3138-8. 27595578 PMC5011931

[22] Shu Y, McCauley J. 2017. GISAID: global initiative on sharing all influenza data—from vision to reality. Eurosurveillance 22:30494. 10.2807/1560-7917.ES.2017.22.13.30494. 28382917 PMC5388101

[23] Hadfield J, Megill C, Bell SM, Huddleston J, Potter B, Callender C, Sagulenko P, Bedford T, Neher RA. 2018. Nextstrain: real-time tracking of pathogen evolution. Bioinformatics 34:4121–4123. 10.1093/bioinformatics/bty407. 29790939 PMC6247931

[24] Edgar RC. 2004. MUSCLE: multiple sequence alignment with high accuracy and high throughput. Nucleic Acids Res 32:1792–1797. 10.1093/nar/gkh340. 15034147 PMC390337

[25] Minh BQ, Schmidt HA, Chernomor O, Schrempf D, Woodhams MD, von Haeseler A, Lanfear R. 2020. IQ-TREE 2: new models and efficient methods for phylogenetic inference in the genomic era. Mol Biol Evol 37:1530–1534. 10.1093/molbev/msaa015. 32011700 PMC7182206

[26] To T-H, Jung M, Lycett S, Gascuel O. 2016. Fast dating using least-squares criteria and algorithms. Syst Biol 65:82–97. 10.1093/sysbio/syv068. 26424727 PMC4678253

[27] Rambaut A, Lam TT, Max Carvalho L, Pybus OG. 2016. Exploring the temporal structure of heterochronous sequences using TempEst (formerly Path-O-Gen). Virus Evol 2:vew007. 10.1093/ve/vew007. 27774300 PMC4989882

[28] Hoang DT, Chernomor O, von Haeseler A, Minh BQ, Vinh LS. 2018. UFBoot2: improving the ultrafast bootstrap approximation. Mol Biol Evol 35:518–522. 10.1093/molbev/msx281. 29077904 PMC5850222

[29] World Health Organization. 2011. Manual for the laboratory diagnosis and virological surveillance of influenza. World Health Organization, Geneva, Switzerland.

[30] Matrosovich M, Matrosovich T, Carr J, Roberts NA, Klenk H-D. 2003. Overexpression of the α-2,6-Sialyltransferase in MDCK cells increases influenza virus sensitivity to neuraminidase inhibitors. J Virol 77:8418–8425. 10.1128/jvi.77.15.8418-8425.2003. 12857911 PMC165236

[31] Lin YP, Gregory V, Collins P, Kloess J, Wharton S, Cattle N, Lackenby A, Daniels R, Hay A. 2010. Neuraminidase receptor binding variants of human influenza A(H3N2) viruses resulting from substitution of aspartic acid 151 in the catalytic site: a role in virus attachment? J Virol 84:6769–6781. 10.1128/JVI.00458-10. 20410266 PMC2903250

[32] Wu NC, Zost SJ, Thompson AJ, Oyen D, Nycholat CM, McBride R, Paulson JC, Hensley SE, Wilson IA. 2017. A structural explanation for the low effectiveness of the seasonal influenza H3N2 vaccine. PLoS Pathog 13:e1006682. 10.1371/journal.ppat.1006682. 29059230 PMC5667890

[33] Krammer F, Palese P, Steel J. 2015.Advances in universal influenza virus vaccine design and antibody mediated therapies based on conserved regions of the hemagglutinin, p. 301–321, *In* Oldstone MBA, Compans RW (ed), Influenza pathogenesis and control—volume II. Springer International Publishing, Cham, Switzerland. 10.1007/82_2014_408 25007847

[34] Koel BF, Burke DF, Bestebroer TM, van der Vliet S, Zondag GCM, Vervaet G, Skepner E, Lewis NS, Spronken MIJ, Russell CA, Eropkin MY, Hurt AC, Barr IG, de Jong JC, Rimmelzwaan GF, Osterhaus ADME, Fouchier RAM, Smith DJ. 2013. Substitutions near the receptor binding site determine major antigenic change during influenza virus evolution. Science 342:976–979. 10.1126/science.1244730. 24264991

[35] Smith DJ, Lapedes AS, de Jong JC, Bestebroer TM, Rimmelzwaan GF, Osterhaus ADME, Fouchier RAM. 2004. Mapping the antigenic and genetic evolution of influenza virus. Science 305:371–376. 10.1126/science.1097211. 15218094

[36] World Health Organization. 2020. Statement on the second meeting of the International Health Regulations (2005) Emergency Committee regarding the outbreak of novel coronavirus (2019-nCoV). https://www.who.int/news/item/30-01-2020-statement-on-the-second-meeting-of-the-international-health-regulations-(2005)-emergency-committee-regarding-the-outbreak-of-novel-coronavirus-(2019-ncov). Accessed April 2021.

[37] World Health Organization. 2020. WHO Director-General's opening remarks at the media briefing on COVID-19—11 March 2020. https://www.who.int/director-general/speeches/detail/who-director-general-s-opening-remarks-at-the-media-briefing-on-covid-19---11-march-2020. Accessed 22 April 2021.

[38] Imai N, Gaythorpe KAM, Abbott S, Bhatia S, van Elsland S, Prem K, Liu Y, Ferguson NM. 2020. Adoption and impact of non-pharmaceutical interventions for COVID-19. Wellcome Open Res 5:59. 10.12688/wellcomeopenres.15808.1. 32529040 PMC7255913

[39] Bedford T, Riley S, Barr IG, Broor S, Chadha M, Cox NJ, Daniels RS, Gunasekaran CP, Hurt AC, Kelso A, Klimov A, Lewis NS, Li X, McCauley JW, Odagiri T, Potdar V, Rambaut A, Shu Y, Skepner E, Smith DJ, Suchard MA, Tashiro M, Wang D, Xu X, Lemey P, Russell CA. 2015. Global circulation patterns of seasonal influenza viruses vary with antigenic drift. Nature 523:217–220. 10.1038/nature14460. 26053121 PMC4499780

