# Supplementary material for: Genetic and Antigenic Characterization of an Influenza A(H3N2) Outbreak in Cambodia and the Greater Mekong Subregion during the COVID-19 Pandemic, 2020
Source: J Virol. 2021 Nov 23;95(24):e01267-21. doi: 10.1128/JVI.01267-21 (PMC8610588; doi:10.1128/JVI.01267-21)
Supplement: Supplemental file 1 — Table S1 (GISAID acknowledgment table.). Download jvi.01267-21-s0001.pdf, PDF file, 1.2 MB [file jvi.01267-21-s0001.pdf]

Isolate_Id	HA Segment_Id	Location	Collection	Isolate_Name	Originating_Lab	Submitting_Lab	Authors
EPI_ISL_1015492	EPI1846786|3001288552_ZZ	Asia / Bangladesh	2020-09-	A/Bangladesh/3010/2	icddr,b International Centre for Diarrhoeal Disease Research,	Centers for Disease Control and Prevention	NA
EPI_ISL_1040050	EPI1847032|A.AbuDhabi/3901	Asia / United Arab Emirates /	2021-01-	A.AbuDhabi/390121/2	Sheikh Khalifa Medical City (SKMC)	Sheikh Khalifa Medical City (SKMC)	Younan, Mary
EPI_ISL_1041017	EPI1847041|N10041171.4	Asia / Cambodia / Phnom Penh	2020-09-	A/Cambodia/e101239	Institute Pasteur du Cambodia	WHO Collaborating Centre for Reference	Deng,Y-M; Iannello,P; Lau,H; Spirason,N;
EPI_ISL_1041018	EPI1847049|N10041172.4	Asia / Cambodia / Khett Svay	2020-09-	A/Cambodia/e101239	Institute Pasteur du Cambodia	WHO Collaborating Centre for Reference	Deng,Y-M; Iannello,P; Lau,H; Spirason,N;
EPI_ISL_1041019	EPI1847056|N10041173.4	Asia / Cambodia / Khett Svay	2020-09-	A/Cambodia/e101242	Institute Pasteur du Cambodia	WHO Collaborating Centre for Reference	Deng,Y-M; Iannello,P; Lau,H; Spirason,N;
EPI_ISL_1041020	EPI1847063|N10041174.4	Asia / Cambodia / Mondul Kiri	2020-09-	A/Cambodia/e101242	Institute Pasteur du Cambodia	WHO Collaborating Centre for Reference	Deng,Y-M; Iannello,P; Lau,H; Spirason,N;
EPI_ISL_1055767	EPI1847684|Infl_21-	Europe / Sweden / Stockholms	2021-01-	A/Stockholm/2/2021	Klinisk mikrobiologi, Karolinska Universitetslaboratoriet, Karolinska	Public Health Agency of Sweden	NA
EPI_ISL_1064117	EPI1847792|3030724507_ZZ	North America / United States /	2021-01-	A/Wisconsin/02/2021	Wisconsin State Laboratory of Hygiene	Centers for Disease Control and Prevention	NA
EPI_ISL_1064120	EPI1847816|3001288673_N8	Asia / Bahrain	2021-01-	A/Bahrain/1344/2021	Ministry of Health Bahrain	Centers for Disease Control and Prevention	NA
EPI_ISL_1064121	EPI1847824|3001288680_N8	Asia / Japan	2020-08-	A/Kitakyusyu/1/2020	National Institute of Infectious Diseases (NIID)	Centers for Disease Control and Prevention	NA
EPI_ISL_1064122	EPI1847832|3001288675_N8	Asia / Bahrain	2021-01-	A/Bahrain/3524/2021	Ministry of Health Bahrain	Centers for Disease Control and Prevention	NA
EPI_ISL_1064123	EPI1847840|3001288676_N8	Asia / Bahrain	2021-01-	A/Bahrain/9211/2021	Ministry of Health Bahrain	Centers for Disease Control and Prevention	NA
EPI_ISL_1064125	EPI1847854|3001288588_N8	Asia / Bangladesh	2020-09-	A/Bangladesh/101007	Institute of Epidemiology Disease Control and Research (IEDCR) &	Centers for Disease Control and Prevention	NA
EPI_ISL_1064127	EPI1847870|3001288674_N8	Asia / Bahrain	2021-01-	A/Bahrain/4981/2021	Ministry of Health Bahrain	Centers for Disease Control and Prevention	NA
EPI_ISL_1064128	EPI1847873|3001288587_N8	Asia / Bangladesh	2020-09-	A/Bangladesh/1006/2	Institute of Epidemiology Disease Control and Research (IEDCR) &	Centers for Disease Control and Prevention	NA
EPI_ISL_1064130	EPI1847888|3001288669_N8	Asia / Vietnam	2020-10-	A/Vietnam/Vnhcm-	Pasteur Institute, Influenza Laboratory	Centers for Disease Control and Prevention	NA
EPI_ISL_1064131	EPI1847896|3001288671_N8	Asia / Vietnam	2020-10-	A/Vietnam/Vnhcm-	Pasteur Institute, Influenza Laboratory	Centers for Disease Control and Prevention	NA
EPI_ISL_1064132	EPI1847904|3001288664_N8	Asia / Vietnam	2020	A/Vietnam/Vnhcm-	Pasteur Institute, Influenza Laboratory	Centers for Disease Control and Prevention	NA
EPI_ISL_1064135	EPI1847928|3001288661_N8	Asia / Vietnam	2020-10-	A/Vietnam/Vnhcm-	Pasteur Institute, Influenza Laboratory	Centers for Disease Control and Prevention	NA
EPI_ISL_1064136	EPI1847936|3001288692_N8	Asia / United Arab Emirates	2021-01-	A/Abu	Shaikh Khalifa Medical City	Centers for Disease Control and Prevention	NA
EPI_ISL_1064137	EPI1847944|3001288666_N8	Asia / Vietnam	2020	A/Vietnam/Vnhcm-	Pasteur Institute, Influenza Laboratory	Centers for Disease Control and Prevention	NA
EPI_ISL_1064140	EPI1847968|3001288667_N8	Asia / Vietnam	2020-10-	A/Vietnam/Vnhcm-	Pasteur Institute, Influenza Laboratory	Centers for Disease Control and Prevention	NA
EPI_ISL_1064141	EPI1847976|3001288668_N8	Asia / Vietnam	2020-10-	A/Vietnam/Vnhcm-	Pasteur Institute, Influenza Laboratory	Centers for Disease Control and Prevention	NA
EPI_ISL_1064142	EPI1847984|3001288662_N8	Asia / Vietnam	2020-10-	A/Vietnam/Vnhcm-	Pasteur Institute, Influenza Laboratory	Centers for Disease Control and Prevention	NA
EPI_ISL_1064143	EPI1847992|3001288665_N8	Asia / Vietnam	2020	A/Vietnam/Vnhcm-	Pasteur Institute, Influenza Laboratory	Centers for Disease Control and Prevention	NA
EPI_ISL_1064144	EPI1848000|3001288670_N8	Asia / Vietnam	2020-10-	A/Vietnam/Vnhcm-	Pasteur Institute, Influenza Laboratory	Centers for Disease Control and Prevention	NA
EPI_ISL_1064145	EPI1848008|3001288672_N8	Asia / Vietnam	2020	A/Vietnam/Vnhcm-	Pasteur Institute, Influenza Laboratory	Centers for Disease Control and Prevention	NA
EPI_ISL_1064146	EPI1848016|3001288663_N8	Asia / Vietnam	2020	A/Vietnam/Vnhcm-	Pasteur Institute, Influenza Laboratory	Centers for Disease Control and Prevention	NA
EPI_ISL_1242033	EPI1850509|A/India/Pun-	Asia / India / Maharashtra /	2020-10-	A/India/Pun-	ICMR-National Institute of Virology	ICMR-National Institute of Virology	Potdar,V;Saha,U
EPI_ISL_1242039	EPI1850510|A/India/Pun-	Asia / India / Maharashtra /	2020-11-	A/India/Pun-	ICMR-National Institute of Virology	ICMR-National Institute of Virology	Potdar,V;Saha,U
EPI_ISL_1246959	EPI1850511|A/India/Pun-	Asia / India / Maharashtra /	2021-01-	A/India/Pun-	ICMR-National Institute of Virology	ICMR-National Institute of Virology	Potdar,V;Saha,U
EPI_ISL_1246963	EPI1850512|A/India/Pun-	Asia / India / Maharashtra /	2021-01-	A/India/Pun-	ICMR-National Institute of Virology	ICMR-National Institute of Virology	Potdar,V;Saha,U
EPI_ISL_1292812	EPI1851803|N10042117.4	Oceania / Australia / Northern	2021-03-	A/Darwin/3/2021	Influenza Surveillance Centre for Disease Control	WHO Collaborating Centre for Reference	Deng,Y-M; Iannello,P; Lau,H; Spirason,N;
EPI_ISL_1292813	EPI1851811|N10042103.4	Oceania / Australia / Northern	2021-02-	A/Darwin/1/2021	Influenza Surveillance Centre for Disease Control	WHO Collaborating Centre for Reference	Deng,Y-M; Iannello,P; Lau,H; Spirason,N;
EPI_ISL_1292814	EPI1851819|N10042104.4	Oceania / Australia / Northern	2021-02-	A/Darwin/2/2021	Influenza Surveillance Centre for Disease Control	WHO Collaborating Centre for Reference	Deng,Y-M; Iannello,P; Lau,H; Spirason,N;
EPI_ISL_1296297	EPI1851875|3001288738_N8	Africa / Nigeria	2020-11-	A/Nigeria/4970/2020	NCDC Public Health Reference Laboratory	Centers for Disease Control and Prevention	NA
EPI_ISL_1296298	EPI1851883|3001288739_N8	Africa / Nigeria	2020-11-	A/Nigeria/4976/2020	NCDC Public Health Reference Laboratory	Centers for Disease Control and Prevention	NA
EPI_ISL_1296301	EPI1851907|3001288743_N8	Africa / Nigeria	2020-11-	A/Nigeria/5059/2020	NCDC Public Health Reference Laboratory	Centers for Disease Control and Prevention	NA
EPI_ISL_1296304	EPI1851931|3001288740_N8	Africa / Nigeria	2020-11-	A/Nigeria/4995/2020	NCDC Public Health Reference Laboratory	Centers for Disease Control and Prevention	NA
EPI_ISL_1296305	EPI1851939|3001288736_N8	Africa / Nigeria	2020-11-	A/Nigeria/4959/2020	NCDC Public Health Reference Laboratory	Centers for Disease Control and Prevention	NA
EPI_ISL_1296308	EPI1851963|3001288767_N8	Africa / Congo, the Democatic	2020-12-	A/Congo/559/2020	INRB Service de Virologie	Centers for Disease Control and Prevention	NA
EPI_ISL_1296309	EPI1851971|3001288772_N8	Africa / Congo, the Democatic	2021-01-	A/Congo/19/2021	INRB Service de Virologie	Centers for Disease Control and Prevention	NA
EPI_ISL_1296310	EPI1851979|3001288768_N8	Africa / Congo, the Democatic	2020-12-	A/Congo/560/2020	INRB Service de Virologie	Centers for Disease Control and Prevention	NA
EPI_ISL_1296311	EPI1851987|3001288773_N8	Africa / Congo, the Democatic	2021-01-	A/Congo/23/2021	INRB Service de Virologie	Centers for Disease Control and Prevention	NA
EPI_ISL_1296312	EPI1851992|3001288766_N8	Africa / Congo, the Democatic	2020-11-	A/Congo/543/2020	INRB Service de Virologie	Centers for Disease Control and Prevention	NA
EPI_ISL_1296314	EPI1852006|3001288770_N8	Africa / Congo, the Democatic	2021-01-	A/Congo/07/2021	INRB Service de Virologie	Centers for Disease Control and Prevention	NA
EPI_ISL_1296315	EPI1852014|3001288771_N8	Africa / Congo, the Democatic	2021-01-	A/Congo/15/2021	INRB Service de Virologie	Centers for Disease Control and Prevention	NA
EPI_ISL_1296317	EPI1852022|3001288775_N8	Africa / Congo, the Democatic	2021-01-	A/Congo/44/2021	INRB Service de Virologie	Centers for Disease Control and Prevention	NA
EPI_ISL_1296318	EPI1852030|3001288704_N8	Asia / Thailand	2020-03-	A/Ayutthaya/67/2020	WHO National Influenza Centre, National Institute of Medical Research	Centers for Disease Control and Prevention	NA
EPI_ISL_1296320	EPI1852046|3001288829_N8	Asia / Cambodia	2020-09-	A/Cambodia/996/2020	National Public Health Laboratory	Centers for Disease Control and Prevention	NA
EPI_ISL_1296322	EPI1852062|3001288833_N8	Asia / Cambodia	2020-09-	A/Cambodia/1040/202	National Public Health Laboratory	Centers for Disease Control and Prevention	NA
EPI_ISL_1296323	EPI1852070|3001288697_N8	Asia / Thailand	2020-11-	A/Ayutthaya/60/2020	WHO National Influenza Centre, National Institute of Medical Research	Centers for Disease Control and Prevention	NA
EPI_ISL_1296324	EPI1852078|3001288834_N8	Asia / Cambodia	2020-09-	A/Cambodia/1044/202	National Public Health Laboratory	Centers for Disease Control and Prevention	NA
EPI_ISL_1296325	EPI1852086|3001288821_N8	Asia / Cambodia	2020-08-	A/Cambodia/719/2020	National Public Health Laboratory	Centers for Disease Control and Prevention	NA
EPI_ISL_1296327	EPI1852102|3001288698_N8	Asia / Thailand	2020-11-	A/Ayutthaya/61/2020	WHO National Influenza Centre, National Institute of Medical Research	Centers for Disease Control and Prevention	NA
EPI_ISL_1296328	EPI1852110|3001288696_N8	Asia / Thailand	2020-11-	A/Bangkok/59/2020	WHO National Influenza Centre, National Institute of Medical Research	Centers for Disease Control and Prevention	NA
EPI_ISL_1296329	EPI1852118|3001288830_N8	Asia / Cambodia	2020-09-	A/Cambodia/998/2020	National Public Health Laboratory	Centers for Disease Control and Prevention	NA
EPI_ISL_1296330	EPI1852126|3001288819_N8	Asia / Cambodia	2020-08-	A/Cambodia/709/2020	National Public Health Laboratory	Centers for Disease Control and Prevention	NA
EPI_ISL_1296331	EPI1852134|3001288823_N8	Asia / Cambodia	2020-08-	A/Cambodia/731/2020	National Public Health Laboratory	Centers for Disease Control and Prevention	NA
EPI_ISL_1296332	EPI1852142|3001288824_N8	Asia / Cambodia	2020-08-	A/Cambodia/780/2020	National Public Health Laboratory	Centers for Disease Control and Prevention	NA
EPI_ISL_1296333	EPI1852150|3001288826_N8	Asia / Cambodia	2020-08-	A/Cambodia/796/2020	National Public Health Laboratory	Centers for Disease Control and Prevention	NA
EPI_ISL_1296334	EPI1852158|3001288822_N8	Asia / Cambodia	2020-08-	A/Cambodia/727/2020	National Public Health Laboratory	Centers for Disease Control and Prevention	NA
EPI_ISL_1296335	EPI1852166|3001288832_N8	Asia / Cambodia	2020-09-	A/Cambodia/984/2020	National Public Health Laboratory	Centers for Disease Control and Prevention	NA
EPI_ISL_1296336	EPI1852174|3001288820_N8	Asia / Cambodia	2020-08-	A/Cambodia/713/2020	National Public Health Laboratory	Centers for Disease Control and Prevention	NA
EPI_ISL_1296337	EPI1852181|3001288700_N8	Asia / Thailand	2020-03-	A/Nonthaburi/63/2020	WHO National Influenza Centre, National Institute of Medical Research	Centers for Disease Control and Prevention	NA

**EPI_ISL_1296338**	EPI1852189|3001288827_N8	Asia / Cambodia	2020-08-	A/Cambodia/801/2020	National Public Health Laboratory	Centers for Disease Control and Prevention	NA
**EPI_ISL_1296339**	EPI1852197|3001288828_N8	Asia / Cambodia	2020-08-	A/Cambodia/805/2020	National Public Health Laboratory	Centers for Disease Control and Prevention	NA
**EPI_ISL_1296340**	EPI1852205|3001288695_N8	Asia / Thailand	2020-11-	A/Bangkok/58/2020	WHO National Influenza Centre, National Institute of Medical Research	Centers for Disease Control and Prevention	NA
**EPI_ISL_1296342**	EPI1852221|3001288693_N8	Asia / Thailand	2020-11-	A/Ayutthaya/56/2020	WHO National Influenza Centre, National Institute of Medical Research	Centers for Disease Control and Prevention	NA
**EPI_ISL_1296343**	EPI1852229|3001288699_N8	Asia / Thailand	2020-11-	A/Bangkok/62/2020	WHO National Influenza Centre, National Institute of Medical Research	Centers for Disease Control and Prevention	NA
**EPI_ISL_1296344**	EPI1852237|3001288709_N8	Asia / Thailand	2020-03-	A/Bangkok/72/2020	WHO National Influenza Centre, National Institute of Medical Research	Centers for Disease Control and Prevention	NA
**EPI_ISL_1296362**	EPI1852380|3001288831_N8	Asia / Cambodia	2020-09-	A/Cambodia/981/2020	National Public Health Laboratory	Centers for Disease Control and Prevention	NA
**EPI_ISL_1296363**	EPI1852388|3001288694_N8	Asia / Thailand	2020-11-	A/Bangkok/57/2020	WHO National Influenza Centre, National Institute of Medical Research	Centers for Disease Control and Prevention	NA
**EPI_ISL_1367567**	EPI1853354|3001288806_ZZ	Africa / Niger	2021-01-	A/Niger/8749/2021	Centre de Recherche Medicale et Sanitaire (CERMES)	Centers for Disease Control and Prevention	NA
**EPI_ISL_1367571**	EPI1853386|3001288785_ZZ	Africa / Niger	2020-03-	A/Niger/7852/2020	Centre de Recherche Medicale et Sanitaire (CERMES)	Centers for Disease Control and Prevention	NA
**EPI_ISL_1367578**	EPI1853441|3001288787_ZZ	Africa / Niger	2020-03-	A/Niger/7870/2020	Centre de Recherche Medicale et Sanitaire (CERMES)	Centers for Disease Control and Prevention	NA
**EPI_ISL_1367584**	EPI1853486|3001288810_N8	Africa / Niger	2021-01-	A/Niger/8776/2021	Centre de Recherche Medicale et Sanitaire (CERMES)	Centers for Disease Control and Prevention	NA
**EPI_ISL_1367585**	EPI1853494|3001288786_N8	Africa / Niger	2020-03-	A/Niger/7868/2020	Centre de Recherche Medicale et Sanitaire (CERMES)	Centers for Disease Control and Prevention	NA
**EPI_ISL_1367587**	EPI1853510|3001288811_N8	Africa / Niger	2020-12-	A/Niger/8635/2020	Centre de Recherche Medicale et Sanitaire (CERMES)	Centers for Disease Control and Prevention	NA
**EPI_ISL_1367589**	EPI1853525|3001288816_N8	Africa / Niger	2020-12-	A/Niger/8590/2020	Centre de Recherche Medicale et Sanitaire (CERMES)	Centers for Disease Control and Prevention	NA
**EPI_ISL_1367590**	EPI1853533|3001288814_N8	Africa / Niger	2021-01-	A/Niger/8773/2021	Centre de Recherche Medicale et Sanitaire (CERMES)	Centers for Disease Control and Prevention	NA
**EPI_ISL_1367591**	EPI1853540|3001288804_N8	Africa / Niger	2020-12-	A/Niger/8609/2020	Centre de Recherche Medicale et Sanitaire (CERMES)	Centers for Disease Control and Prevention	NA
**EPI_ISL_1367592**	EPI1853548|3001288784_N8	Africa / Niger	2020-03-	A/Niger/7845/2020	Centre de Recherche Medicale et Sanitaire (CERMES)	Centers for Disease Control and Prevention	NA
**EPI_ISL_1367593**	EPI1853556|3001288812_N8	Africa / Niger	2020-12-	A/Niger/8581/2020	Centre de Recherche Medicale et Sanitaire (CERMES)	Centers for Disease Control and Prevention	NA
**EPI_ISL_1386235**	EPI1853975|A/YAMAGATA/1/	Asia / Japan	2021-02-	A/YAMAGATA/1/2021	Yamagata Prefectural Institute of Public Health	National Institute of Infectious Diseases	Takashita,Emi;Fujisaki,Seiichiro;Shirakura,Masa
**EPI_ISL_1386236**	EPI1853983|A/YAMAGATA/2/	Asia / Japan	2021-02-	A/YAMAGATA/2/2021	Yamagata Prefectural Institute of Public Health	National Institute of Infectious Diseases	Takashita,Emi;Fujisaki,Seiichiro;Shirakura,Masa
**EPI_ISL_1455649**	EPI1856003|3001288825_N8	Asia / Cambodia	2020-08-	A/Cambodia/793/2020	National Public Health Laboratory	Centers for Disease Control and Prevention	NA
**EPI_ISL_1455650**	EPI1856011|3001288839_N8	Asia / Cambodia	2020-10-	A/Cambodia/1167/202	National Public Health Laboratory	Centers for Disease Control and Prevention	NA
**EPI_ISL_1455651**	EPI1856019|3001288843_N8	Asia / Cambodia	2020-10-	A/Cambodia/1189/202	National Public Health Laboratory	Centers for Disease Control and Prevention	NA
**EPI_ISL_1455652**	EPI1856027|3001288842_N8	Asia / Cambodia	2020-10-	A/Cambodia/1180/202	National Public Health Laboratory	Centers for Disease Control and Prevention	NA
**EPI_ISL_1455653**	EPI1856035|3001288841_N8	Asia / Cambodia	2020-10-	A/Cambodia/1176/202	National Public Health Laboratory	Centers for Disease Control and Prevention	NA
**EPI_ISL_1455654**	EPI1856043|3001288840_N8	Asia / Cambodia	2020-10-	A/Cambodia/1171/202	National Public Health Laboratory	Centers for Disease Control and Prevention	NA
**EPI_ISL_1455655**	EPI1856051|3001288847_N8	Asia / Cambodia	2020-08-	A/Cambodia/3651/202	National Public Health Laboratory	Centers for Disease Control and Prevention	NA
**EPI_ISL_1455656**	EPI1856059|3001288845_N8	Asia / Cambodia	2020-08-	A/Cambodia/3645/202	National Public Health Laboratory	Centers for Disease Control and Prevention	NA
**EPI_ISL_1455657**	EPI1856067|3001288844_N8	Asia / Cambodia	2020-08-	A/Cambodia/3641/202	National Public Health Laboratory	Centers for Disease Control and Prevention	NA
**EPI_ISL_1455658**	EPI1856075|3001288846_N8	Asia / Cambodia	2020-08-	A/Cambodia/3648/202	National Public Health Laboratory	Centers for Disease Control and Prevention	NA
**EPI_ISL_1519296**	EPI1857102|74-	Africa / Cameroon	2020-02-	A/Cameroon/1292/20	Centre Pasteur du Cameroun	Crick Worldwide Influenza Centre	NA
**EPI_ISL_1519328**	EPI1857105|68-	Africa / Cameroon	2020-10-	A/Cameroon/16950/2	Centre Pasteur du Cameroun	Crick Worldwide Influenza Centre	NA
**EPI_ISL_1519345**	EPI1857107|66-	Africa / Cameroon	2020-11-	A/Cameroon/16967/2	Centre Pasteur du Cameroun	Crick Worldwide Influenza Centre	NA
**EPI_ISL_1519352**	EPI1857109|62-	Africa / Cameroon	2020-11-	A/Cameroon/16984/2	Centre Pasteur du Cameroun	Crick Worldwide Influenza Centre	NA
**EPI_ISL_1519353**	EPI1857111|59-	Africa / Cameroon	2020-12-	A/Cameroon/16995/2	Centre Pasteur du Cameroun	Crick Worldwide Influenza Centre	NA
**EPI_ISL_1519354**	EPI1857113|60-	Africa / Cameroon	2020-12-	A/Cameroon/16996/2	Centre Pasteur du Cameroun	Crick Worldwide Influenza Centre	NA
**EPI_ISL_1563628**	EPI1857216|S10042175.4,	Oceania / Australia / Northern	2021-03-	A/Darwin/6/2021	Influenza Surveillance Centre for Disease Control	WHO Collaborating Centre for Reference	Deng,Y-M; Iannello,P; Lau,H; Spirason,N;
**EPI_ISL_1563629**	EPI1857219|S10042316.4,	Oceania / Australia / Northern	2021-03-	A/Darwin/7/2021	Influenza Surveillance Centre for Disease Control	WHO Collaborating Centre for Reference	Deng,Y-M; Iannello,P; Lau,H; Spirason,N;
**EPI_ISL_1563630**	EPI1857393|S10042315.4,	Oceania / Australia / Northern	2021-03-	A/Darwin/6a/2021	Influenza Surveillance Centre for Disease Control	WHO Collaborating Centre for Reference	Deng,Y-M; Iannello,P; Lau,H; Spirason,N;
**EPI_ISL_1625835**	EPI1857517|3000824258_N8	Europe / Ukraine	2020-03-	A/Dnipro/414/2020	Institute of Epidemiology and Infectious Diseases AMS of Ukraine	Centers for Disease Control and Prevention	NA
**EPI_ISL_165592**	EPI539694|14S1720	Oceania / Australia / South	2014-06-	A/SOUTH	Institute of Medical and Veterinary Science (IMVS)	WHO Collaborating Centre for Reference	Deng,Y-M.; Iannello,P.; Spirason,N.; Jelley,L.;
**EPI_ISL_166994**	EPI544500|3000092267_2590	North America / United States /	2014-09-	A/Michigan/15/2014	Michigan Department of Community Health	Centers for Disease Control and Prevention	NA
**EPI_ISL_168126**	EPI549053|2014768580_2600	North America / United States /	2014-07-	A/Hawaii/47/2014	State of Hawaii Department of Health	Centers for Disease Control and Prevention	NA
**EPI_ISL_168972**	EPI551870|a-hk-7127-	Asia / Hong Kong (SAR)	2014-07-	A/Hong	Government Virus Unit	National Institute for Medical Research	NA
**EPI_ISL_169307**	EPI552913|3000093155_2605	North America / United States /	2014-11-	A/New Jersey/26/2014	New Jersey Department of Health & Senior Services	Centers for Disease Control and Prevention	NA
**EPI_ISL_170086**	EPI556469|A/SAITAMA/103/2	Asia / Japan	2014-11-	A/SAITAMA/103/2014	Saitama Institute of Public Health	National Institute of Infectious Diseases	Takashita,Emi; Fujisaki,Seiichiro;
**EPI_ISL_1718306**	EPI1858564|A/India/Pun-	Asia / India / Maharashtra /	2021-02-	A/India/Pun-	ICMR-National Institute of Virology	ICMR-National Institute of Virology	Dr.Varsha Potdar , Ujjayni Saha
**EPI_ISL_1718307**	EPI1858565|A/India/Pun-	Asia / India / Maharashtra /	2021-03-	A/India/Pun-	ICMR-National Institute of Virology	ICMR-National Institute of Virology	Potdar,V;Saha,U
**EPI_ISL_1718310**	EPI1858566|A/India/Pun-	Asia / India / Maharashtra /	2021-03-	A/India/Pun-	ICMR-National Institute of Virology	ICMR-National Institute of Virology	Potdar,V;Saha,U
**EPI_ISL_1732519**	EPI1858630|3001288377_N8	Africa / Mali	2020-02-	A/Mali/20048/2020	NIC Lab CVD-MALI	Centers for Disease Control and Prevention	NA
**EPI_ISL_1732520**	EPI1858638|3001288386_N8	Africa / Mali	2020-03-	A/Mali/20027/2020	NIC Lab CVD-MALI	Centers for Disease Control and Prevention	NA
**EPI_ISL_1732521**	EPI1858646|3001288416_N8	Asia / Lao, People's	2020-09-	A/Laos/1740/2020	National Center for Laboratory and Epidemiology	Centers for Disease Control and Prevention	NA
**EPI_ISL_1732522**	EPI1858654|3030724513_ZZ	North America / United States /	2021-03-	A/Pennsylvania/01/20	Pennsylvania Department of Health	Centers for Disease Control and Prevention	NA
**EPI_ISL_1760813**	EPI1858894|Infl_21-	Europe / Sweden / Stockholms	2021-02-	A/Stockholm/3/2021	Klinisk mikrobiologi, Karolinska Universitetslaboratoriet, Karolinska	Public Health Agency of Sweden	NA
**EPI_ISL_1760817**	EPI1858926|Infl_21-	Europe / Sweden / Stockholms	2021-03-	A/Stockholm/4/2021	Klinisk mikrobiologi, Karolinska Universitetslaboratoriet, Karolinska	Public Health Agency of Sweden	NA
**EPI_ISL_176732**	EPI579796|3000383562_2674	North America / United States /	2015-02-	A/Wisconsin/20/2015	Wisconsin State Laboratory of Hygiene	Centers for Disease Control and Prevention	NA
**EPI_ISL_1913253**	EPI1859160|A/Netherlands/10	Europe / Netherlands	2021-02-	A/Netherlands/10000/	Academic Medical Center, University of Amsterdam	National Institute for Public Health and the	Meijer, A.;van den Brink, S.;Vennema,
**EPI_ISL_191709**	EPI624328|3000383983_2710	North America / United States /	2015-04-	A/Wisconsin/66/2015	Wisconsin State Laboratory of Hygiene	Centers for Disease Control and Prevention	NA
**EPI_ISL_192696**	EPI629582|3000411777_2725	South America / Peru	2015-04-	A/Peru/27/2015	Laboratorio de Referencia Nacional Virus Respiratorios, Instituto	Centers for Disease Control and Prevention	NA
**EPI_ISL_193166**	EPI630727|a-fiji-2-2015h3M	Oceania / Australia	2015-03-	A/Fiji/2/2015	WHO Collaborating Centre for Reference and Research on Influenza	Crick Worldwide Influenza Centre	NA
**EPI_ISL_200769**	EPI668343|3000413769_2745	North America / United States /	2015-09-	A/Montana/28/2015	Montana Laboratory Services Bureau	Centers for Disease Control and Prevention	NA
**EPI_ISL_202884**	EPI677158|3000413733_2745	North America / United States /	2015-09-	A/Alaska/232/2015	Alaska State Virology Lab	Centers for Disease Control and Prevention	NA
**EPI_ISL_202892**	EPI677218|3000413741_2745	North America / United States /	2015-09-	A/Alaska/240/2015	Alaska State Virology Lab	Centers for Disease Control and Prevention	NA
**EPI_ISL_202916**	EPI677406|3000413918_2810	North America / United States /	2015-10-	A/Nebraska/19/2015	Nebraska Public Health Lab	Centers for Disease Control and Prevention	NA
**EPI_ISL_208627**	EPI702064|3000411453_2827	North America / Canada	2015-11-	A/Ontario/RV2414/201	National Microbiology Laboratory, Public Health Agency of Canada	Centers for Disease Control and Prevention	NA
**EPI_ISL_210374**	EPI706697|A/SAPPORO/71/2	Asia / Japan	2015-12-	A/SAPPORO/71/2015	Sapporo City Institute of Public Health	National Institute of Infectious Diseases	Takashita,Emi; Fujisaki,Seiichiro;
**EPI_ISL_2178976**	EPI1859848|A/Moscow/CRIE/	Europe / Russian Federation /	2021-05-	A/Moscow/CRIE/2/202	Center of Hygiene and Epidemiology in Moscow	Central Research Institute of Epidemiology	NA

**EPI_ISL_219238**	EPI747840|3025625713_ZZY	North America / United States /	2016-03-	A/Nevada/22/2016	Nevada State Health Laboratory	Centers for Disease Control and Prevention	NA
**EPI_ISL_220299**	EPI752312|3000477690_2884	North America / United States /	2016-02-	A/Texas/88/2016	Texas Childrens Hospital	Centers for Disease Control and Prevention	NA
**EPI_ISL_2233234**	EPI1859978|N10042503.4	Oceania / Australia / Northern	2021-02-	A/Darwin/12/2021	Influenza Surveillance Centre for Disease Control	WHO Collaborating Centre for Reference	Deng,Y-M; Iannello,P; Lau,H; Spirason,N;
**EPI_ISL_2233235**	EPI1859986|N10042505.4	Oceania / Australia / Northern	2021-02-	A/Darwin/11/2021	Influenza Surveillance Centre for Disease Control	WHO Collaborating Centre for Reference	Deng,Y-M; Iannello,P; Lau,H; Spirason,N;
**EPI_ISL_2233240**	EPI1859996|S10042636.4	Oceania / Australia / Northern	2021-04-	A/Darwin/9/2021	Influenza Surveillance Centre for Disease Control	WHO Collaborating Centre for Reference	Deng,Y-M; Iannello,P; Lau,H; Spirason,N;
**EPI_ISL_2233242**	EPI1860001|A/Nepal/20FL167	Asia / Nepal	2020-09-	A/Nepal/20FL1671/20	National Public Health Laboratory	National Institute of Infectious Diseases	Takashita,Emi;Fujisaki,Seiichiro;Watanabe,Shinji
**EPI_ISL_2233243**	EPI1860004|A/Nepal/20FL231	Asia / Nepal	2020-12-	A/Nepal/20FL2312/20	National Public Health Laboratory	National Institute of Infectious Diseases	Takashita,Emi;Fujisaki,Seiichiro;Watanabe,Shinji
**EPI_ISL_2233244**	EPI1860007|A/Nepal/21FL020	Asia / Nepal	2021-02-	A/Nepal/21FL0201/20	National Public Health Laboratory	National Institute of Infectious Diseases	Takashita,Emi;Fujisaki,Seiichiro;Watanabe,Shinji
**EPI_ISL_2234357**	EPI1860015|Infl_21-	Europe / Sweden / Stockholms	2021-04-	A/Stockholm/5/2021	Klinisk mikrobiologi, Karolinska Universitetslaboratoriet, Karolinska	Public Health Agency of Sweden	NA
**EPI_ISL_2234358**	EPI1860023|Infl_21-	Europe / Sweden / Stockholms	2021-04-	A/Stockholm/6/2021	Klinisk mikrobiologi, Karolinska Universitetslaboratoriet, Karolinska	Public Health Agency of Sweden	NA
**EPI_ISL_2245215**	EPI1861016|3030638114_ZZ	North America / United States /	2020-03-	A/Pennsylvania/253/2	Children's Hospital of Pittsburgh UPMC	Centers for Disease Control and Prevention	NA
**EPI_ISL_2245216**	EPI1861023|3030638445_ZZ	North America / United States /	2020-03-	A/Pennsylvania/231/2	Children's Hospital of Pittsburgh UPMC	Centers for Disease Control and Prevention	NA
**EPI_ISL_2245459**	EPI1861031|3001288904_N8	Asia / Lao, People's	2021-03-	A/Laos/799/2021	National Center for Laboratory and Epidemiology	Centers for Disease Control and Prevention	NA
**EPI_ISL_2245460**	EPI1861039|3001288901_N8	Asia / Lao, People's	2021-03-	A/Laos/766/2021	National Center for Laboratory and Epidemiology	Centers for Disease Control and Prevention	NA
**EPI_ISL_2245461**	EPI1861047|3001288903_N8	Asia / Lao, People's	2021-02-	A/Laos/418/2021	National Center for Laboratory and Epidemiology	Centers for Disease Control and Prevention	NA
**EPI_ISL_2245462**	EPI1861055|3001288900_N8	Asia / Lao, People's	2021-02-	A/Laos/527/2021	National Center for Laboratory and Epidemiology	Centers for Disease Control and Prevention	NA
**EPI_ISL_2245463**	EPI1861063|3001288897_N8	Asia / Lao, People's	2021-02-	A/Laos/504/2021	National Center for Laboratory and Epidemiology	Centers for Disease Control and Prevention	NA
**EPI_ISL_2245464**	EPI1861071|3001288896_N8	Asia / Lao, People's	2021-02-	A/Laos/469/2021	National Center for Laboratory and Epidemiology	Centers for Disease Control and Prevention	NA
**EPI_ISL_2245465**	EPI1861079|3001288899_N8	Asia / Lao, People's	2021-02-	A/Laos/580/2021	National Center for Laboratory and Epidemiology	Centers for Disease Control and Prevention	NA
**EPI_ISL_2245466**	EPI1861087|3001288905_N8	Asia / Lao, People's	2021-03-	A/Laos/785/2021	National Center for Laboratory and Epidemiology	Centers for Disease Control and Prevention	NA
**EPI_ISL_2245467**	EPI1861095|3001288902_N8	Asia / Lao, People's	2021-02-	A/Laos/694/2021	National Center for Laboratory and Epidemiology	Centers for Disease Control and Prevention	NA
**EPI_ISL_2246006**	EPI1861522|3030638120_ZZ	North America / United States /	2020-03-	A/Pennsylvania/255/2	Children's Hospital of Pittsburgh UPMC	Centers for Disease Control and Prevention	NA
**EPI_ISL_2258201**	EPI1863794|3001876382_N8	North America / United States /	2019-12-	A/New York/64/2019	Vanderbilt University Medical Center	Centers for Disease Control and Prevention	NA
**EPI_ISL_2258202**	EPI1863802|3001872754_N8	North America / United States /	2020-01-	A/Colorado/12/2020	Vanderbilt University Medical Center	Centers for Disease Control and Prevention	NA
**EPI_ISL_2258204**	EPI1863818|3001870324_N8	North America / United States /	2019-12-	A/Massachusetts/47/2	Vanderbilt University Medical Center	Centers for Disease Control and Prevention	NA
**EPI_ISL_225834**	EPI780183|A/Singapore/INFIM	Asia / Singapore	2016-06-	A/Singapore/INFIMH-	Ministry of Health, Singapore	Ministry of Health, Singapore	Chen,B.; Phuah,S.P.; Mak,T.M.; Chen,S.L.;
**EPI_ISL_227637**	EPI793162|16S1067	Oceania / Australia / New	2016-03-	A/Newcastle/30/2016	John Hunter Hospital	WHO Collaborating Centre for Reference	Deng,Y-M.; Iannello,P.; Sprirason,N.; Lau,H.;
**EPI_ISL_227639**	EPI793168|16S1070	Oceania / Australia / Australian	2016-04-	A/Canberra/7/2016	Canberra Hospital	WHO Collaborating Centre for Reference	Deng,Y-M.; Iannello,P.; Sprirason,N.; Lau,H.;
**EPI_ISL_230303**	EPI813945|3000480154_N8K	Africa / Madagascar	2016-04-	A/Antananarivo/1067/	Institut Pasteur de Madagascar	Centers for Disease Control and Prevention	NA
**EPI_ISL_230320**	EPI814081|3000477609_2952	North America / United States /	2016-06-	A/Idaho/33/2016	State of Idaho Bureau of Laboratories	Centers for Disease Control and Prevention	NA
**EPI_ISL_233441**	EPI832213|3000480485_N8K	North America / United States /	2016-05-	A/Arizona/67/2016	Arizona Department of Health Services	Centers for Disease Control and Prevention	NA
**EPI_ISL_2365356**	EPI1868356|A/India/Pun-	Asia / India / Maharashtra /	2021-01-	A/India/Pun-	ICMR-National Institute of Virology	ICMR-National Institute of Virology	Potdar,V;Saha,U
**EPI_ISL_2365358**	EPI1868357|A/India/Pun-	Asia / India / Maharashtra /	2021-02-	A/India/Pun-	ICMR-National Institute of Virology	ICMR-National Institute of Virology	Potdar,V;Saha,U
**EPI_ISL_2365359**	EPI1868358|A/India/Pun-	Asia / India / Maharashtra /	2021-02-	A/India/Pun-	ICMR-National Institute of Virology	ICMR-National Institute of Virology	Potdar,V;Saha,U
**EPI_ISL_2365360**	EPI1868359|A/India/Pun-	Asia / India / Maharashtra /	2021-02-	A/India/Pun-	ICMR-National Institute of Virology	ICMR-National Institute of Virology	Potdar,V;Saha,U
**EPI_ISL_2365361**	EPI1868360|A/Inida/Pun-	Asia / India / Maharashtra /	2021-02-	A/Inida/Pun-	ICMR-National Institute of Virology	ICMR-National Institute of Virology	Potdar,V;Saha,U
**EPI_ISL_2365362**	EPI1868361|A/India/Pun-	Asia / India / Maharashtra /	2021-04-	A/India/Pun-	ICMR-National Institute of Virology	ICMR-National Institute of Virology	Potdar,V;Saha,U
**EPI_ISL_239648**	EPI868010|3025627742_ZZY	North America / United States /	2016-11-	A/Delaware/32/2016	Delaware Public Health Lab	Centers for Disease Control and Prevention	NA
**EPI_ISL_239701**	EPI868424|3025627845_ZZY	North America / United States /	2016-11-	A/Maryland/23/2016	Maryland Department of Health and Mental Hygiene	Centers for Disease Control and Prevention	NA
**EPI_ISL_239828**	EPI868931|16S2822	Oceania / Australia / South	2016-11-	A/South	Institute of Medical and Veterinary Science (IMVS)	WHO Collaborating Centre for Reference	Deng,Y-M.; Iannello,P.; Spirason,N.; Lau,H.;
**EPI_ISL_240790**	EPI874032|A/Singapore/TT13	Asia / Singapore	2016-12-	A/Singapore/TT1374/2	NA	Ministry of Health, Singapore	NA
**EPI_ISL_240836**	EPI874394|3025627947_ZZY	North America / United States /	2016-12-	A/California/179/2016	California Department of Health Services	Centers for Disease Control and Prevention	NA
**EPI_ISL_241590**	EPI879722|3025628092_ZZY	North America / United States /	2016-12-	A/Washington/106/20	Washington State Public Health Laboratory	Centers for Disease Control and Prevention	NA
**EPI_ISL_2484031**	EPI1868855|A/India/Pun-	Asia / India / Maharashtra /	2021-05-	A/India/Pun-	ICMR-National Institute of Virology	ICMR-National Institute of Virology	V,Potdar;U,Saha;M,Das
**EPI_ISL_2484033**	EPI1868861|A/India/Pun-	Asia / India / Maharashtra /	2021-05-	A/India/Pun-	ICMR-National Institute of Virology	ICMR-National Institute of Virology	V,Potdar;U,Saha;M,Das
**EPI_ISL_249201**	EPI919282|17S0455.4	Oceania / Australia /	2016-12-	A/Brisbane/321/2016	Queensland Health Forensic and Scientific Services	WHO Collaborating Centre for Reference	Deng,Y-M.; Iannello,P.; Spirason,N.; Lau,H.;
**EPI_ISL_249207**	EPI919300|17S0461.4	Oceania / Australia /	2016-12-	A/Brisbane/318/2016	Queensland Health Forensic and Scientific Services	WHO Collaborating Centre for Reference	Deng,Y-M.; Iannello,P.; Spirason,N.; Lau,H.;
**EPI_ISL_2517229**	EPI1869526|3002056826_N8	North America / United States /	2020-03-	A/Michigan/170/2020	University of Michigan SPH EPID	Centers for Disease Control and Prevention	NA
**EPI_ISL_2518731**	EPI1869534|3030724516_ZZ	North America / United States /	2021-04-	A/Delaware/01/2021	Delaware Public Health Lab	Centers for Disease Control and Prevention	NA
**EPI_ISL_252740**	EPI941682|3025628793_ZZY	North America / United States /	2017-01-	A/Washington/16/201	Washington State Public Health Laboratory	Centers for Disease Control and Prevention	NA
**EPI_ISL_258358**	EPI972987|3025629373_ZZY	North America / United States /	2017-03-	A/Texas/68/2017	Dallas County Health and Human Services	Centers for Disease Control and Prevention	NA
**EPI_ISL_258392**	EPI973255|3025629489_ZZY	North America / United States /	2017-03-	A/Texas/71/2017	San Antonio Metropolitan Health	Centers for Disease Control and Prevention	NA
**EPI_ISL_259723**	EPI979325|3025629553_ZZY	North America / United States /	2017-03-	A/Florida/23/2017	Florida Department of Health-Jacksonville	Centers for Disease Control and Prevention	NA
**EPI_ISL_269024**	EPI1021210|MS-a-hong_kong-	Asia / Hong Kong (SAR)	2017-05-	A/Hong	Government Virus Unit	Crick Worldwide Influenza Centre	NA
**EPI_ISL_269771**	EPI1023828|A/Singapore/TT0	Asia / Singapore	2017-05-	A/Singapore/TT0555/2	Ministry of Health, Singapore	Ministry of Health, Singapore	Chen,B.; Phuah,S.P.; Mak,T.M.; Chen,S.L.;
**EPI_ISL_277940**	EPI1060375|17S3661.4	Oceania / Australia / Victoria /	2017-08-	A/Victoria/653/2017	Monash Medical Centre	WHO Collaborating Centre for Reference	Deng,Y-M; Iannello,P; Lau,H; Kaye,M; Todd,A;
**EPI_ISL_282838**	EPI1085812|3025631094_ZZ	North America / United States /	2017-09-	A/Wisconsin/327/2017	Wisconsin State Laboratory of Hygiene	Centers for Disease Control and Prevention	NA
**EPI_ISL_283128**	EPI1087624|A/Bretagne/1413/	Europe / France / Bretagne	2017-10-	A/Bretagne/1413/2017	NA	Institut Pasteur	NA
**EPI_ISL_288989**	EPI1126242|A/YOKOHAMA/1	Asia / Japan	2017-09-	A/YOKOHAMA/145/20	Yokohama City Institute of Public Health.	National Institute of Infectious Diseases	Kuwahara,Tomoko;Takashita,Emi;Fujisaki,Seiic
**EPI_ISL_292575**	EPI1146345|3026015402_ZZ	North America / United States /	2017-12-	A/Kansas/14/2017	Kansas Department of Health and Environment	Centers for Disease Control and Prevention	NA
**EPI_ISL_294252**	EPI1154878|MS-a-switzerland-	Europe / Switzerland	2017-12-	A/Switzerland/8060/20	Hopital Cantonal Universitaire de Geneves	Crick Worldwide Influenza Centre	NA
**EPI_ISL_298379**	EPI1174591|3000686682_N8	South America / Peru	2017-12-	A/Peru/3817/2017	Laboratorio de Referencia Nacional Virus Respiratorios, Instituto	Centers for Disease Control and Prevention	NA
**EPI_ISL_305018**	EPI1205544|3026016780_ZZ	North America / United States /	2018-02-	A/New	New Mexico Department of Health	Centers for Disease Control and Prevention	NA
**EPI_ISL_306954**	EPI1215421|A/NAGANO/2731	Asia / Japan	2017-11-	A/NAGANO/2731/201	Nagano Environmental Conservation Research Institute	National Institute of Infectious Diseases	Kuwahara,Tomoko;Takashita,Emi;Fujisaki,Seiic
**EPI_ISL_307872**	EPI1221483|3000828283_N8	Asia / United Arab Emirates	2018-01-	A/Abu Dhabi/240/2018	Shaikh Khalifa Medical City	Centers for Disease Control and Prevention	NA
**EPI_ISL_312267**	EPI1243195|MS-a-hong_kong-	Asia / Hong Kong (SAR)	2018-04-	A/Hong	Government Virus Unit	Crick Worldwide Influenza Centre	NA
**EPI_ISL_314014**	EPI1251865|18S0886.4	Oceania / Australia / New	2018-03-	A/Sydney/22/2018	Clinical Virology Unit, CDIM	WHO Collaborating Centre for Reference	Deng, Y-M; Iannello,P; Lau,H; Kaye,M; Todd,A;
**EPI_ISL_314925**	EPI1256086|MS-a-la_rioja-	Europe / Spain	2018-04-	A/LaRioja/2202/2018	Instituto de Salud Carlos III	Crick Worldwide Influenza Centre	NA

**EPI_ISL_322718**	EPI1281499|N10006107.4	Oceania / Australia /	2018-07-	A/Brisbane/117/2018	Queensland Health Forensic and Scientific Services	WHO Collaborating Centre for Reference	NA
**EPI_ISL_322728**	EPI1281529|N10006117.4	Oceania / New Caledonia /	2018-02-	A/New	Institut Pasteur New Caledonia	WHO Collaborating Centre for Reference	NA
**EPI_ISL_335436**	EPI1341950|S10011362.4	Oceania / Australia / New	2018-12-	A/Newcastle/82/2018	John Hunter Hospital	WHO Collaborating Centre for Reference	Deng,Y-M; Iannello,P; Lau,H; Kaye,M; Todd,A;
**EPI_ISL_346283**	EPI1387331|N10014601.4	Oceania / Australia / South	2019-02-	A/South	Institute of Medical and Veterinary Science (IMVS)	WHO Collaborating Centre for Reference	Deng,Y-M; Iannello,P; Lau,H; Todd,A;
**EPI_ISL_347938**	EPI1397376|3001584404_N8	Asia / Hong Kong (SAR)	2018-12-	A/Hong Kong/45/2019	Government Virus Unit	Centers for Disease Control and Prevention	NA
**EPI_ISL_363864**	EPI1486401|3026052349_ZZ	North America / United States /	2019-05-	A/Hawaii/42/2019	State of Hawaii Department of Health	Centers for Disease Control and Prevention	NA
**EPI_ISL_363971**	EPI1487239|3026052274_ZZ	North America / United States /	2019-05-	A/Delaware/39/2019	Delaware Public Health Lab	Centers for Disease Control and Prevention	NA
**EPI_ISL_365480**	EPI1493861|A/Kirov/CRIE/416/	Europe / Russian Federation /	2019-06-	A/Kirov/CRIE/416/201	Center of Hygiene and Epidemiology in Kirov oblast	Central Research Institute of Epidemiology	NA
**EPI_ISL_365746**	EPI1495629|3026052627_ZZ	North America / United States /	2019-06-	A/Minnesota/44/2019	Minnesota Department of Health	Centers for Disease Control and Prevention	NA
**EPI_ISL_368196**	EPI1508126|3026052694_ZZ	North America / United States /	2019-06-	A/Arizona/31/2019	Arizona Department of Health Services	Centers for Disease Control and Prevention	NA
**EPI_ISL_368199**	EPI1508150|3026052700_ZZ	North America / United States /	2019-06-	A/Florida/80/2019	Florida Department of Health-Tampa	Centers for Disease Control and Prevention	NA
**EPI_ISL_368202**	EPI1508174|3026052708_ZZ	North America / United States /	2019-07-	A/Wisconsin/556/2019	Wisconsin State Laboratory of Hygiene	Centers for Disease Control and Prevention	NA
**EPI_ISL_368205**	EPI1508198|3026052713_ZZ	North America / United States /	2019-06-	A/Hawaii/48/2019	State of Hawaii Department of Health	Centers for Disease Control and Prevention	NA
**EPI_ISL_377300**	EPI1543094|MS-a-hk-2669-	Asia / Hong Kong (SAR)	2019-06-	A/Hong	Government Virus Unit	Crick Worldwide Influenza Centre	NA
**EPI_ISL_377302**	EPI1543098|MS-a-hk-2671-	Asia / Hong Kong (SAR)	2019-06-	A/Hong	Government Virus Unit	Crick Worldwide Influenza Centre	NA
**EPI_ISL_377303**	EPI1543100|MS-a-hk-2672-	Asia / Hong Kong (SAR)	2019-06-	A/Hong	Government Virus Unit	Crick Worldwide Influenza Centre	NA
**EPI_ISL_377304**	EPI1543102|MS-a-hk-2673-	Asia / Hong Kong (SAR)	2019-06-	A/Hong	Government Virus Unit	Crick Worldwide Influenza Centre	NA
**EPI_ISL_377305**	EPI1543104|MS-a-hk-2674-	Asia / Hong Kong (SAR)	2019-06-	A/Hong	Government Virus Unit	Crick Worldwide Influenza Centre	NA
**EPI_ISL_377307**	EPI1543108|MS-a-hk-2676-	Asia / Hong Kong (SAR)	2019-06-	A/Hong	Government Virus Unit	Crick Worldwide Influenza Centre	NA
**EPI_ISL_377832**	EPI1546521|3001293206_N8	North America / Jamaica	2019-06-	A/Jamaica/2063/2019	University of the West Indies	Centers for Disease Control and Prevention	NA
**EPI_ISL_378034**	EPI1548131|3026052727_ZZ	North America / United States /	2019-07-	A/Nebraska/11/2019	Nebraska Public Health Lab	Centers for Disease Control and Prevention	NA
**EPI_ISL_378084**	EPI1548511|3026052743_ZZ	North America / United States /	2019-07-	A/Wisconsin/558/2019	Wisconsin State Laboratory of Hygiene	Centers for Disease Control and Prevention	NA
**EPI_ISL_378086**	EPI1548527|3026052746_ZZ	North America / United States /	2019-06-	A/Michigan/387/2019	Michigan Department of Community Health	Centers for Disease Control and Prevention	NA
**EPI_ISL_380083**	EPI1557659|3001293370_N8	North America / Guatemala	2019-06-	A/Guatemala/10/2019	Laboratorio Nacional De Salud Guatemala	Centers for Disease Control and Prevention	NA
**EPI_ISL_381721**	EPI1566708|A/TOKYO/19121/	Asia / Japan	2019-06-	A/TOKYO/19121/2019	Tokyo Metropolitan Institute of Public Health	National Institute of Infectious Diseases	Kuwahara,Tomoko;Takashita,Emi;Fujisaki,Seiic
**EPI_ISL_382014**	EPI1568581|A/Santiago/61056	South America / Chile / Region	2019-06-	A/Santiago/61056/201	Instituto de Salud Publica de Chile	Instituto de Salud Publica de Chile	Tognarelli, J.; Lagos, J.; Arata, L.; Fasce, R.;
**EPI_ISL_382025**	EPI1568662|A/Santiago/62358	South America / Chile / Region	2019-07-	A/Santiago/62358/201	Instituto de Salud Publica de Chile	Instituto de Salud Publica de Chile	Tognarelli, J.; Lagos, J.; Arata, L.; Fasce, R.;
**EPI_ISL_386834**	EPI1573613|3026052773_ZZ	North America / United States /	2019-07-	A/Texas/357/2019	Texas Department of State Health Services-Laboratory Services	Centers for Disease Control and Prevention	NA
**EPI_ISL_386844**	EPI1573692|3026052774_ZZ	North America / United States /	2019-06-	A/Indiana/34/2019	Indiana State Department of Health Laboratories	Centers for Disease Control and Prevention	NA
**EPI_ISL_386845**	EPI1573700|3026052772_ZZ	North America / United States /	2019-07-	A/Texas/358/2019	Texas Department of State Health Services-Laboratory Services	Centers for Disease Control and Prevention	NA
**EPI_ISL_386847**	EPI1573718|3026052767_ZZ	North America / United States /	2019-07-	A/North	North Dakota Department of Health	Centers for Disease Control and Prevention	NA
**EPI_ISL_386865**	EPI1573862|3001293300_N8	Asia / Thailand	2019-06-	A/Nakhonphanom/498	WHO National Influenza Centre, National Institute of Medical Research	Centers for Disease Control and Prevention	NA
**EPI_ISL_386964**	EPI1574632|A/Iquique/63421/	South America / Chile / Iquique	2019-06-	A/Iquique/63421/2019	Instituto de Salud Publica de Chile	Instituto de Salud Publica de Chile	Tognarelli, J.; Lagos, J.; Arata, L.; Fasce, R.;
**EPI_ISL_386972**	EPI1574694|A/Santiago/53729	South America / Chile / Region	2019-06-	A/Santiago/53729/201	Instituto de Salud Publica de Chile	Instituto de Salud Publica de Chile	Tognarelli, J.; Lagos, J.; Arata, L.; Fasce, R.;
**EPI_ISL_386973**	EPI1574701|A/Santiago/54567	South America / Chile / Region	2019-06-	A/Santiago/54567/201	Instituto de Salud Publica de Chile	Instituto de Salud Publica de Chile	Tognarelli, J.; Lagos, J.; Arata, L.; Fasce, R.;
**EPI_ISL_386974**	EPI1574709|A/Santiago/60419	South America / Chile / Region	2019-06-	A/Santiago/60419/201	Instituto de Salud Publica de Chile	Instituto de Salud Publica de Chile	Tognarelli, J.; Lagos, J.; Arata, L.; Fasce, R.;
**EPI_ISL_387041**	EPI1574973|MS-a-iceland-	Europe / Iceland	2019-06-	A/Iceland/12621/2019	Landspitali - University Hospital	Crick Worldwide Influenza Centre	NA
**EPI_ISL_389068**	EPI1583457|S10025025.4	Oceania / Australia / Victoria /	2019-07-	A/Victoria/169/2019	Victorian Infectious Diseases Reference Laboratory	WHO Collaborating Centre for Reference	Deng,Y-M; Iannello,P; Lau,H; Todd,A;
**EPI_ISL_389223**	EPI1583856|74-	Asia / Oman	2019-07-	A/Oman/4471/2019	Central Public Health Laboratory, Ministry of Health	Crick Worldwide Influenza Centre	NA
**EPI_ISL_389224**	EPI1583858|73-	Asia / Oman	2019-07-	A/Oman/4518/2019	Central Public Health Laboratory, Ministry of Health	Crick Worldwide Influenza Centre	NA
**EPI_ISL_389256**	EPI1583922|75-	Asia / Oman	2019-06-	A/Oman/4262/2019	Central Public Health Laboratory, Ministry of Health	Crick Worldwide Influenza Centre	NA
**EPI_ISL_389283**	EPI1583994|S10027760.4	Oceania / Australia / Victoria /	2019-07-	A/Victoria/177/2019	Victorian Infectious Diseases Reference Laboratory	WHO Collaborating Centre for Reference	Deng,Y-M; Iannello,P; Lau,H; Todd,A;
**EPI_ISL_389325**	EPI1584107|A/Cardiff/9746/20	Europe / United Kingdom / City	2019-06-	A/Cardiff/9746/2019	Public Health Wales Microbiology Cardiff	Public Health Wales Microbiology Cardiff	NA
**EPI_ISL_389327**	EPI1584122|A/Port	Europe / United Kingdom /	2019-07-	A/Port	Public Health Wales Microbiology Cardiff	Public Health Wales Microbiology Cardiff	NA
**EPI_ISL_389329**	EPI1584138|A/Wrexham/3245	Europe / United Kingdom /	2019-07-	A/Wrexham/3245/201	Public Health Wales Microbiology Cardiff	Public Health Wales Microbiology Cardiff	NA
**EPI_ISL_389491**	EPI1584420|A/Espirito_Santo/	South America / Brazil / Estado	2019-06-	A/Espirito	LACEN/ES - Laboratório Central de Saúde Pública do Espírito Santo	Instituto Oswaldo Cruz FIOCRUZ -	NIC, FIOCRUZ
**EPI_ISL_389563**	EPI1584570|S10027748.4	Oceania / Australia / Victoria /	2019-08-	A/Victoria/223/2019	Victorian Infectious Diseases Reference Laboratory	WHO Collaborating Centre for Reference	Deng,Y-M; Iannello,P; Lau,H; Todd,A;
**EPI_ISL_390012**	EPI1586160|3001293478_N8	Asia / Hong Kong (SAR)	2019-07-	A/Hong	Government Virus Unit	Centers for Disease Control and Prevention	NA
**EPI_ISL_390014**	EPI1586176|3001293477_N8	Asia / Hong Kong (SAR)	2019-07-	A/Hong	Government Virus Unit	Centers for Disease Control and Prevention	NA
**EPI_ISL_390016**	EPI1586192|3001293475_N8	Asia / Hong Kong (SAR)	2019-07-	A/Hong	Government Virus Unit	Centers for Disease Control and Prevention	NA
**EPI_ISL_390036**	EPI1586333|3026052781_ZZ	North America / United States /	2019-07-	A/Florida/85/2019	Florida Department of Health-Tampa	Centers for Disease Control and Prevention	NA
**EPI_ISL_390064**	EPI1586550|3001293598_N8	Asia / Bangladesh	2019-06-	A/Bangladesh/319060	icddr,b International Centre for Diarrhoeal Disease Research,	Centers for Disease Control and Prevention	NA
**EPI_ISL_390088**	EPI1586730|3026052811_ZZ	North America / United States /	2019-08-	A/Minnesota/50/2019	Minnesota Department of Health	Centers for Disease Control and Prevention	NA
**EPI_ISL_390090**	EPI1586746|3001293459_N8	South America / Argentina	2019-06-	A/Argentina/1532/201	Instituto Nacional de Enfermedades Infecciosas	Centers for Disease Control and Prevention	NA
**EPI_ISL_390097**	EPI1586801|3001293452_N8	South America / Argentina	2019-07-	A/Argentina/1422/201	Instituto Nacional de Enfermedades Infecciosas	Centers for Disease Control and Prevention	NA
**EPI_ISL_390113**	EPI1586939|3001293482_N8	Asia / Hong Kong (SAR)	2019-07-	A/Hong	Government Virus Unit	Centers for Disease Control and Prevention	NA
**EPI_ISL_390124**	EPI1587022|3001293856_N8	Africa / Kenya	2019-07-	A/Kenya/6516/2019	CDC-Kenya	Centers for Disease Control and Prevention	NA
**EPI_ISL_390137**	EPI1587127|3001293798_N8	South America / Argentina	2019-07-	A/Argentina/14373/20	Intituto Nacional de Epidemiologia	Centers for Disease Control and Prevention	NA
**EPI_ISL_390200**	EPI1587617|3001293813_N8	South America / Chile	2019-07-	A/Santiago/62822/201	Instituto de Salud Publica de Chile	Centers for Disease Control and Prevention	NA
**EPI_ISL_390201**	EPI1587625|3001293812_N8	South America / Chile	2019-07-	A/Santiago/62164/201	Instituto de Salud Publica de Chile	Centers for Disease Control and Prevention	NA
**EPI_ISL_390203**	EPI1587641|3001293810_N8	South America / Chile	2019-07-	A/Talca/63219/2019	Instituto de Salud Publica de Chile	Centers for Disease Control and Prevention	NA
**EPI_ISL_390582**	EPI1589586|S10028337.4	Oceania / Australia / New	2019-07-	A/Sydney/1109/2019	Institute of Medical and Veterinary Science (IMVS)	WHO Collaborating Centre for Reference	Deng,Y-M; Iannello,P; Lau,H; Todd,A;
**EPI_ISL_390583**	EPI1589588|S10028343.4	Oceania / Australia / Western	2019-07-	A/Perth/1127/2019	Institute of Medical and Veterinary Science (IMVS)	WHO Collaborating Centre for Reference	Deng,Y-M; Iannello,P; Lau,H; Todd,A;
**EPI_ISL_390590**	EPI1589952|S10028906.4,	Oceania / Australia / Victoria /	2019-07-	A/Victoria/205/2019	Victorian Infectious Diseases Reference Laboratory	WHO Collaborating Centre for Reference	Deng,Y-M; Iannello,P; Lau,H; Todd,A;
**EPI_ISL_390596**	EPI1589614|S10028913.4,	Oceania / Australia / Victoria /	2019-08-	A/Victoria/213/2019	Victorian Infectious Diseases Reference Laboratory	WHO Collaborating Centre for Reference	Deng,Y-M; Iannello,P; Lau,H; Todd,A;
**EPI_ISL_390623**	EPI1589660|2019-CX2411.4	Asia / China / Guangxi	2019-08-	A/Guangxi-	WHO Chinese National Influenza Center	WHO Chinese National Influenza Center	Zeng Xiaoxu, Li Xiyan, Dayan Wang
**EPI_ISL_390624**	EPI1589663|2019-CX2410.4	Asia / China / Hunan	2019-08-	A/Hunan-	WHO Chinese National Influenza Center	WHO Chinese National Influenza Center	Zeng Xiaoxu, Li Xiyan, Dayan Wang
**EPI_ISL_390625**	EPI1589667|2019-CX2409.4	Asia / China / Sichuan	2019-08-	A/Sichuan-	WHO Chinese National Influenza Center	WHO Chinese National Influenza Center	Zeng Xiaoxu, Li Xiyan, Dayan Wang

EPI_ISL_390626	EPI1589670|2019-CX2408.4	Asia / China / Guangdong	2019-08-	A/Guangdong-	WHO Chinese National Influenza Center	WHO Chinese National Influenza Center	Zeng Xiaoxu, Li Xiyan, Dayan Wang
EPI_ISL_390627	EPI1589673|2019-CX2407.4	Asia / China / Shanghai	2019-08-	A/Shanghai-	WHO Chinese National Influenza Center	WHO Chinese National Influenza Center	Zeng Xiaoxu, Li Xiyan, Dayan Wang
EPI_ISL_390632	EPI1589688|2019-CX2377.4	Asia / China / Guangxi	2019-07-	A/Guangxi-	WHO Chinese National Influenza Center	WHO Chinese National Influenza Center	Zeng Xiaoxu, Li Xiyan, Dayan Wang
EPI_ISL_390633	EPI1589691|2019-CX2374.4	Asia / China / Guangxi	2019-07-	A/Guangxi-	WHO Chinese National Influenza Center	WHO Chinese National Influenza Center	Zeng Xiaoxu, Li Xiyan, Dayan Wang
EPI_ISL_390658	EPI1589766|2019-CX2375.4	Asia / China / Guangxi	2019-06-	A/Guangxi-	WHO Chinese National Influenza Center	WHO Chinese National Influenza Center	Zeng Xiaoxu, Li Xiyan, Dayan Wang
EPI_ISL_390663	EPI1589781|2019-CX2369.4	Asia / China / Guangdong	2019-07-	A/Guangdong-	WHO Chinese National Influenza Center	WHO Chinese National Influenza Center	Zeng Xiaoxu, Li Xiyan, Dayan Wang
EPI_ISL_390679	EPI1589829|2019-CX2353.4	Asia / China / Guangxi	2019-07-	A/Guangxi-	WHO Chinese National Influenza Center	WHO Chinese National Influenza Center	Zeng Xiaoxu, Li Xiyan, Dayan Wang
EPI_ISL_390693	EPI1589871|2019-CX2329.4	Asia / China / Guangdong	2019-06-	A/Guangdong-	WHO Chinese National Influenza Center	WHO Chinese National Influenza Center	Zeng Xiaoxu, Li Xiyan, Dayan Wang
EPI_ISL_390694	EPI1589874|2019-CX2328.4	Asia / China / Guangdong	2019-07-	A/Guangdong-	WHO Chinese National Influenza Center	WHO Chinese National Influenza Center	Zeng Xiaoxu, Li Xiyan, Dayan Wang
EPI_ISL_390695	EPI1589877|2019-CX2327.4	Asia / China / Guangdong	2019-07-	A/Guangdong-	WHO Chinese National Influenza Center	WHO Chinese National Influenza Center	Zeng Xiaoxu, Li Xiyan, Dayan Wang
EPI_ISL_390701	EPI1589895|2019-CX2321.4	Asia / China / Zhejiang	2019-07-	A/Zhejiang-	WHO Chinese National Influenza Center	WHO Chinese National Influenza Center	Zeng Xiaoxu, Li Xiyan, Dayan Wang
EPI_ISL_390702	EPI1589898|2019-CX2320.4	Asia / China / Zhejiang	2019-07-	A/Zhejiang-	WHO Chinese National Influenza Center	WHO Chinese National Influenza Center	Zeng Xiaoxu, Li Xiyan, Dayan Wang
EPI_ISL_391067	EPI1591142|Infl_19-	Europe / Sweden /	2019-07-	A/Umea/1/2019	Klinisk mikrobiologi, Laboratoriemedicin, Norrlands Universitetssjukhus	Public Health Agency of Sweden	NA
EPI_ISL_391071	EPI1591171|Infl_19-	Europe / Sweden / Stockholms	2019-08-	A/Stockholm/22/2019	Unilabs Laboratoriemedicin Stockholm Solna	Public Health Agency of Sweden	NA
EPI_ISL_391072	EPI1591179|Infl_19-	Europe / Sweden / Stockholms	2019-08-	A/Stockholm/23/2019	Klinisk mikrobiologi, Karolinska Universitetslaboratoriet, Karolinska	Public Health Agency of Sweden	NA
EPI_ISL_391075	EPI1591203|Infl_19-	Europe / Sweden / Vastra	2019-08-	A/Gothenburg/3/2019	Klinisk mikrobiologi, Sahlgrenska Universitetssjukhuset Goteborg	Public Health Agency of Sweden	NA
EPI_ISL_391076	EPI1591211|Infl_19-	Europe / Sweden / Vastra	2019-08-	A/Gothenburg/4/2019	Klinisk mikrobiologi, Sahlgrenska Universitetssjukhuset Goteborg	Public Health Agency of Sweden	NA
EPI_ISL_391079	EPI1591229|Infl_19-	Europe / Sweden / Hallands	2019-09-	A/Halmstad/5/2019	Klinisk mikrobiologi, Hallands sjukhus Halmstad	Public Health Agency of Sweden	NA
EPI_ISL_391081	EPI1591245|Infl_19-	Europe / Sweden / Stockholms	2019-08-	A/Stockholm/27/2019	Public Health Agency of Sweden	Public Health Agency of Sweden	NA
EPI_ISL_391084	EPI1591269|Infl_19-	Europe / Sweden /	2019-08-	A/Norrkoping/1/2019	Public Health Agency of Sweden	Public Health Agency of Sweden	NA
EPI_ISL_391141	EPI1591656|3001293731_N8	Asia / Bangladesh	2019-06-	A/Bangladesh/319061	icddr,b International Centre for Diarrhoeal Disease Research,	Centers for Disease Control and Prevention	NA
EPI_ISL_391143	EPI1591672|3001293736_N8	Asia / Bangladesh	2019-06-	A/Bangladesh/4025/2	icddr,b International Centre for Diarrhoeal Disease Research,	Centers for Disease Control and Prevention	NA
EPI_ISL_391146	EPI1591695|3001293700_N8	Asia / Bangladesh	2019-06-	A/Bangladesh/1012/2	icddr,b International Centre for Diarrhoeal Disease Research,	Centers for Disease Control and Prevention	NA
EPI_ISL_391157	EPI1591759|3001293684_N8	Asia / Bangladesh	2019-06-	A/Bangladesh/719060	icddr,b International Centre for Diarrhoeal Disease Research,	Centers for Disease Control and Prevention	NA
EPI_ISL_391175	EPI1591872|3001293771_N8	Asia / Bangladesh	2019-06-	A/Bangladesh/119061	icddr,b International Centre for Diarrhoeal Disease Research,	Centers for Disease Control and Prevention	NA
EPI_ISL_391188	EPI1591947|3026052897_ZZ	North America / United States /	2019-08-	A/Florida/90/2019	Florida Department of Health-Tampa	Centers for Disease Control and Prevention	NA
EPI_ISL_391191	EPI1591971|3026052826_ZZ	North America / United States /	2019-08-	A/Alaska/38/2019	Alaska State Virology Lab	Centers for Disease Control and Prevention	NA
EPI_ISL_391196	EPI1591997|3026052829_ZZ	North America / United States /	2019-08-	A/Wisconsin/560/2019	Wisconsin State Laboratory of Hygiene	Centers for Disease Control and Prevention	NA
EPI_ISL_391359	EPI1593292|3001293750_N8	Asia / Bangladesh	2019-06-	A/Bangladesh/4015/2	icddr,b International Centre for Diarrhoeal Disease Research,	Centers for Disease Control and Prevention	NA
EPI_ISL_391445	EPI1593948|N10024117.4	Oceania / Australia / Victoria /	2019-06-	A/Victoria/2615/2019	Royal Chidrens Hospital	WHO Collaborating Centre for Reference	Deng,Y-M; Iannello,P; Lau,H; Todd,A;
EPI_ISL_392480	EPI1596890|A/MIE/21/2019_M	Asia / Japan	2019-09-	A/MIE/21/2019	Mie Prefecture Health and Environment Research Institute	National Institute of Infectious Diseases	Kuwahara,Tomoko;Takashita,Emi;Fujisaki,Seiic
EPI_ISL_392482	EPI1596906|A/MIE/23/2019_M	Asia / Japan	2019-09-	A/MIE/23/2019	Mie Prefecture Health and Environment Research Institute	National Institute of Infectious Diseases	Kuwahara,Tomoko;Takashita,Emi;Fujisaki,Seiic
EPI_ISL_392485	EPI1596914|A/TOKYO/19139/	Asia / Japan	2019-06-	A/TOKYO/19139/2019	Tokyo Metropolitan Institute of Public Health	National Institute of Infectious Diseases	Kuwahara,Tomoko;Takashita,Emi;Fujisaki,Seiic
EPI_ISL_392527	EPI1597055|3001289261_N8	South America / Brazil	2019-06-	A/Sao Paulo/1198850-	Instituto Adolfo Lutz	Centers for Disease Control and Prevention	NA
EPI_ISL_392533	EPI1597103|3001289259_N8	South America / Brazil	2019-06-	A/Pariquera-	Instituto Adolfo Lutz	Centers for Disease Control and Prevention	NA
EPI_ISL_392535	EPI1597119|3001289257_N8	South America / Brazil	2019-07-	A/Barueri/1216473-	Instituto Adolfo Lutz	Centers for Disease Control and Prevention	NA
EPI_ISL_392536	EPI1597127|3001289256_N8	South America / Brazil	2019-07-	A/Sao Paulo/1218374-	Instituto Adolfo Lutz	Centers for Disease Control and Prevention	NA
EPI_ISL_392542	EPI1597172|3001289111_N8	South America / Brazil	2019-06-	A/Boituva/863604-	Instituto Adolfo Lutz	Centers for Disease Control and Prevention	NA
EPI_ISL_392569	EPI1597383|3026052958_ZZ	North America / United States /	2019-08-	A/North	North Carolina State Laboratory of Public Health	Centers for Disease Control and Prevention	NA
EPI_ISL_392571	EPI1597399|3026052965_ZZ	North America / United States /	2019-08-	A/Arizona/34/2019	Arizona Department of Health Services	Centers for Disease Control and Prevention	NA
EPI_ISL_392573	EPI1597415|3026052973_ZZ	North America / United States /	2019-08-	A/California/166/2019	California Department of Health Services	Centers for Disease Control and Prevention	NA
EPI_ISL_392582	EPI1597487|3026052985_ZZ	North America / United States /	2019-08-	A/Alaska/39/2019	Alaska State Virology Lab	Centers for Disease Control and Prevention	NA
EPI_ISL_393605	EPI1602341|3001289263_N8	South America / Brazil	2019-08-	A/Sao Paulo/1140710-	Instituto Adolfo Lutz	Centers for Disease Control and Prevention	NA
EPI_ISL_393761	EPI1603533|3001289351_N8	Africa / Kenya	2019-06-	A/Kenya/131/2019	CDC-Kenya	Centers for Disease Control and Prevention	NA
EPI_ISL_393780	EPI1603679|3001289320_N8	Africa / Togo	2019-06-	A/Togo/717/2019	Institute National D'Hygiene	Centers for Disease Control and Prevention	NA
EPI_ISL_394019	EPI1604640|40-	Asia / Lebanon	2019-06-	A/Lebanon/471/2019	Rafic Hariri University Hospital 4th floor DNA/Research Lab	Crick Worldwide Influenza Centre	NA
EPI_ISL_394044	EPI1604690|72-	Africa / Seychelles	2019-06-	A/Seychelles/542/201	Seychelles Public Health Laboratory	Crick Worldwide Influenza Centre	NA
EPI_ISL_394191	EPI1605020|A/MIE/20/2019_M	Asia / Japan	2019-08-	A/MIE/20/2019	Mie Prefecture Health and Environment Research Institute	National Institute of Infectious Diseases	Kuwahara,Tomoko;Takashita,Emi;Fujisaki,Seiic
EPI_ISL_394199	EPI1605042|A/OKINAWA/74/2	Asia / Japan	2019-07-	A/OKINAWA/74/2019	Okinawa Prefectural Institute of Health and Environment	National Institute of Infectious Diseases	Kuwahara,Tomoko;Takashita,Emi;Fujisaki,Seiic
EPI_ISL_394880	EPI1605966|3026053039_ZZ	North America / United States /	2019-09-	A/North	North Dakota Department of Health	Centers for Disease Control and Prevention	NA
EPI_ISL_394886	EPI1606012|3026053050_ZZ	North America / United States /	2019-08-	A/Michigan/436/2019	Michigan Department of Community Health	Centers for Disease Control and Prevention	NA
EPI_ISL_394893	EPI1606067|3026053058_ZZ	North America / United States /	2019-09-	A/Maryland/34/2019	Maryland Department of Health and Mental Hygiene	Centers for Disease Control and Prevention	NA
EPI_ISL_394904	EPI1606153|3001289326_N8	Africa / Togo	2019-07-	A/Togo/856/2019	Institute National D'Hygiene	Centers for Disease Control and Prevention	NA
EPI_ISL_394932	EPI1606376|3001289461_N8	North America / Mexico	2019-07-	A/Mexico/2353/2019	Laboratorio de Virus Respiratorio	Centers for Disease Control and Prevention	NA
EPI_ISL_394944	EPI1606467|3001289460_N8	North America / Mexico	2019-07-	A/Mexico/2350/2019	Laboratorio de Virus Respiratorio	Centers for Disease Control and Prevention	NA
EPI_ISL_394949	EPI1606507|3001289354_N8	Africa / Kenya	2019-07-	A/Kenya/135/2019	CDC-Kenya	Centers for Disease Control and Prevention	NA
EPI_ISL_395038	EPI1607165|3026053083_ZZ	North America / United States /	2019-09-	A/Arizona/39/2019	Arizona Department of Health Services	Centers for Disease Control and Prevention	NA
EPI_ISL_395048	EPI1607239|A/Cannes/1846/2	Europe / France	2019-09-	A/Cannes/1846/2019	CNR Virus des Infections Respiratoires - France SUD	CNR Virus des Infections Respiratoires -	NA
EPI_ISL_395049	EPI1607241|A/St_Laurent_du	Europe / France	2019-09-	A/St_Laurent_du_Var/	CNR Virus des Infections Respiratoires - France SUD	CNR Virus des Infections Respiratoires -	NA
EPI_ISL_395050	EPI1607243|A/St_Laurent_du	Europe / France	2019-09-	A/St_Laurent_du_Var/	CNR Virus des Infections Respiratoires - France SUD	CNR Virus des Infections Respiratoires -	NA
EPI_ISL_395114	EPI1607710|3001289430_N8	South America / Bolivia,	2019-06-	A/Bolivia/1232/2019	Instituto Nacional de Laboratoriosde Salud (INLASA)	Centers for Disease Control and Prevention	NA
EPI_ISL_395144	EPI1607896|3001289534_N8	Asia / Bangladesh	2019-07-	A/Bangladesh/4013/2	icddr,b International Centre for Diarrhoeal Disease Research,	Centers for Disease Control and Prevention	NA
EPI_ISL_395196	EPI1608295|A/YOKOHAMA/1	Asia / Japan	2019-09-	A/YOKOHAMA/166/20	Yokohama City Institute of Public Health.	National Institute of Infectious Diseases	Kuwahara,Tomoko;Takashita,Emi;Fujisaki,Seiic
EPI_ISL_395317	EPI1608968|Infl_19-	Europe / Sweden / Stockholms	2019-09-	A/Stockholm/29/2019	Klinisk mikrobiologi, Karolinska Universitetslaboratoriet, Karolinska	Public Health Agency of Sweden	NA
EPI_ISL_395379	EPI1609095|3026053183_ZZ	North America / United States /	2019-09-	A/Hawaii/67/2019	State of Hawaii Department of Health	Centers for Disease Control and Prevention	NA
EPI_ISL_395416	EPI1609383|3001289625_N8	Asia / Bangladesh	2019-07-	A/Bangladesh/8029/2	icddr,b International Centre for Diarrhoeal Disease Research,	Centers for Disease Control and Prevention	NA
EPI_ISL_395417	EPI1609391|3001289624_N8	Asia / Bangladesh	2019-07-	A/Bangladesh/319070	icddr,b International Centre for Diarrhoeal Disease Research,	Centers for Disease Control and Prevention	NA
EPI_ISL_395532	EPI1610273|A/YOKOHAMA/1	Asia / Japan	2019-09-	A/YOKOHAMA/168/20	Yokohama City Institute of Public Health.	National Institute of Infectious Diseases	Kuwahara,Tomoko;Takashita,Emi;Fujisaki,Seiic

**EPI_ISL_396179**	EPI1611351|A/Brisbane/1/201	Oceania / Australia	2018-01-	A/Brisbane/1/2018	NA	NA	Tan,G.; Fedorova,N.; Amedeo,P.; Isom,R.;
**EPI_ISL_396988**	EPI1617668|3026053313_ZZ	North America / United States /	2019-10-	A/Idaho/28/2019	State of Idaho Bureau of Laboratories	Centers for Disease Control and Prevention	NA
**EPI_ISL_397169**	EPI1618730|3001289499_N8	Asia / Bangladesh	2019-06-	A/Bangladesh/340/20	Institute of Epidemiology Disease Control and Research (IEDCR) &	Centers for Disease Control and Prevention	NA
**EPI_ISL_397198**	EPI1618957|3001289828_N8	North America / Nicaragua	2019-09-	A/Nicaragua/1341/201	Laboratorio de Virologia, Direccion de Microbiologia	Centers for Disease Control and Prevention	NA
**EPI_ISL_397226**	EPI1619175|3026053414_ZZ	North America / United States /	2019-10-	A/Iowa/53/2019	Iowa State Hygienic Laboratory	Centers for Disease Control and Prevention	NA
**EPI_ISL_397227**	EPI1619183|3026053109_ZZ	North America / United States /	2019-09-	A/Florida/103/2019	Florida Department of Health-Tampa	Centers for Disease Control and Prevention	NA
**EPI_ISL_397228**	EPI1619191|3026053416_ZZ	North America / United States /	2019-10-	A/Iowa/54/2019	Iowa State Hygienic Laboratory	Centers for Disease Control and Prevention	NA
**EPI_ISL_397276**	EPI1619468|55-	Europe / Norway	2019-09-	A/Norway/2282/2019	WHO National Influenza Centre	Crick Worldwide Influenza Centre	NA
**EPI_ISL_397279**	EPI1619473|51-	Europe / Norway	2019-10-	A/Norway/2294/2019	WHO National Influenza Centre	Crick Worldwide Influenza Centre	NA
**EPI_ISL_397283**	EPI1619481|58-	Europe / Norway	2019-10-	A/Norway/2298/2019	WHO National Influenza Centre	Crick Worldwide Influenza Centre	NA
**EPI_ISL_397286**	EPI1619487|95-A_Qatar_10-	Asia / Qatar	2019-08-	A/Qatar/10-VI-19-	Supreme Health Council	Crick Worldwide Influenza Centre	NA
**EPI_ISL_397290**	EPI1619494|86-A_Qatar_10-	Asia / Qatar	2019-08-	A/Qatar/10-VI-19-	Supreme Health Council	Crick Worldwide Influenza Centre	NA
**EPI_ISL_397291**	EPI1619496|85-A_Qatar_10-	Asia / Qatar	2019-08-	A/Qatar/10-VI-19-	Supreme Health Council	Crick Worldwide Influenza Centre	NA
**EPI_ISL_397294**	EPI1619502|88-A_Qatar_13-	Asia / Qatar	2019-08-	A/Qatar/13-VI-19-	Supreme Health Council	Crick Worldwide Influenza Centre	NA
**EPI_ISL_397296**	EPI1619506|92-A_Qatar_16-	Asia / Qatar	2019-08-	A/Qatar/16-VI-19-	Supreme Health Council	Crick Worldwide Influenza Centre	NA
**EPI_ISL_397301**	EPI1619516|83-A_Qatar_16-	Asia / Qatar	2019-08-	A/Qatar/16-VI-19-	Supreme Health Council	Crick Worldwide Influenza Centre	NA
**EPI_ISL_398207**	EPI1626810|A/Singapore/MO	Asia / Singapore	2019-06-	A/Singapore/MOH012	Ministry of Health, Singapore	Ministry of Health, Singapore	Chen,B.;Zhou, Z.; Mak,T.M.; Cui,L; Lin,R.T.P.
**EPI_ISL_398208**	EPI1626818|A/Singapore/GP0	Asia / Singapore	2019-06-	A/Singapore/GP0985/	Ministry of Health, Singapore	Ministry of Health, Singapore	Chen,B.;Zhou, Z.; Mak,T.M.; Cui,L; Lin,R.T.P.
**EPI_ISL_398209**	EPI1626826|A/Singapore/GP1	Asia / Singapore	2019-06-	A/Singapore/GP1116/	Ministry of Health, Singapore	Ministry of Health, Singapore	Chen,B.;Zhou, Z.; Mak,T.M.; Cui,L; Lin,R.T.P.
**EPI_ISL_398211**	EPI1626842|A/Singapore/GP1	Asia / Singapore	2019-07-	A/Singapore/GP1502/	Ministry of Health, Singapore	Ministry of Health, Singapore	Chen,B.;Zhou, Z.; Mak,T.M.; Cui,L; Lin,R.T.P.
**EPI_ISL_398217**	EPI1626890|A/Singapore/TT0	Asia / Singapore	2019-06-	A/Singapore/TT0476/2	Ministry of Health, Singapore	Ministry of Health, Singapore	Chen,B.;Zhou, Z.; Mak,T.M.; Cui,L; Lin,R.T.P.
**EPI_ISL_398220**	EPI1626914|A/Singapore/TT0	Asia / Singapore	2019-07-	A/Singapore/TT0543/2	Ministry of Health, Singapore	Ministry of Health, Singapore	Chen,B.;Zhou, Z.; Mak,T.M.; Cui,L; Lin,R.T.P.
**EPI_ISL_398221**	EPI1626922|A/Singapore/TT0	Asia / Singapore	2019-07-	A/Singapore/TT0558/2	Ministry of Health, Singapore	Ministry of Health, Singapore	Chen,B.;Zhou, Z.; Mak,T.M.; Cui,L; Lin,R.T.P.
**EPI_ISL_398225**	EPI1626954|A/Singapore/NTF	Asia / Singapore	2019-06-	A/Singapore/NTF0040	Ministry of Health, Singapore	Ministry of Health, Singapore	Chen,B.;Zhou, Z.; Mak,T.M.; Cui,L; Lin,R.T.P.
**EPI_ISL_398228**	EPI1626978|A/Singapore/KK1	Asia / Singapore	2019-06-	A/Singapore/KK1356/	Ministry of Health, Singapore	Ministry of Health, Singapore	Chen,B.;Zhou, Z.; Mak,T.M.; Cui,L; Lin,R.T.P.
**EPI_ISL_398231**	EPI1627002|A/Singapore/KK1	Asia / Singapore	2019-06-	A/Singapore/KK1314/	Ministry of Health, Singapore	Ministry of Health, Singapore	Chen,B.;Zhou, Z.; Mak,T.M.; Cui,L; Lin,R.T.P.
**EPI_ISL_398232**	EPI1627010|A/Singapore/KK1	Asia / Singapore	2019-07-	A/Singapore/KK1617/	Ministry of Health, Singapore	Ministry of Health, Singapore	Chen,B.;Zhou, Z.; Mak,T.M.; Cui,L; Lin,R.T.P.
**EPI_ISL_398302**	EPI1627368|A/NIIGATA/805/2	Asia / Japan	2019-08-	A/NIIGATA/805/2019	Niigata Prefectural Institute of Public Health and Environmental Sciences	National Institute of Infectious Diseases	Kuwahara,Tomoko;Takashita,Emi;Fujisaki,Seiic
**EPI_ISL_398304**	EPI1627372|A/OSAKA-	Asia / Japan	2019-08-	A/OSAKA-	Osaka City Institute of Public Health and Environmental Sciences	National Institute of Infectious Diseases	Kuwahara,Tomoko;Takashita,Emi;Fujisaki,Seiic
**EPI_ISL_398305**	EPI1627374|A/EHIME/84/2019	Asia / Japan	2019-07-	A/EHIME/84/2019	Ehime Prefecture Institute of Public Health and Environmental Science	National Institute of Infectious Diseases	Kuwahara,Tomoko;Takashita,Emi;Fujisaki,Seiic
**EPI_ISL_398337**	EPI1627604|3001289739_N8	South America / Brazil	2019-08-	A/Parana/908/2019	Instituto Oswaldo Cruz FIOCRUZ - Laboratory of Respiratory Viruses	Centers for Disease Control and Prevention	NA
**EPI_ISL_398340**	EPI1627628|3001289515_N8	Asia / Bangladesh	2019-06-	A/Bangladesh/1054/2	Institute of Epidemiology Disease Control and Research (IEDCR) &	Centers for Disease Control and Prevention	NA
**EPI_ISL_398347**	EPI1627683|3001289666_N8	Asia / Bangladesh	2019-08-	A/Bangladesh/119081	icddr,b International Centre for Diarrhoeal Disease Research,	Centers for Disease Control and Prevention	NA
**EPI_ISL_398348**	EPI1627691|3001289665_N8	Asia / Bangladesh	2019-08-	A/Bangladesh/119081	icddr,b International Centre for Diarrhoeal Disease Research,	Centers for Disease Control and Prevention	NA
**EPI_ISL_398352**	EPI1627723|3001289661_N8	Asia / Bangladesh	2019-08-	A/Bangladesh/319080	icddr,b International Centre for Diarrhoeal Disease Research,	Centers for Disease Control and Prevention	NA
**EPI_ISL_398353**	EPI1627731|3001289660_N8	Asia / Bangladesh	2019-08-	A/Bangladesh/319081	icddr,b International Centre for Diarrhoeal Disease Research,	Centers for Disease Control and Prevention	NA
**EPI_ISL_398356**	EPI1627755|3001289656_N8	Asia / Bangladesh	2019-08-	A/Bangladesh/319081	icddr,b International Centre for Diarrhoeal Disease Research,	Centers for Disease Control and Prevention	NA
**EPI_ISL_398358**	EPI1627771|3001289653_N8	Asia / Bangladesh	2019-08-	A/Bangladesh/119080	icddr,b International Centre for Diarrhoeal Disease Research,	Centers for Disease Control and Prevention	NA
**EPI_ISL_398361**	EPI1627794|3001289649_N8	Asia / Bangladesh	2019-08-	A/Bangladesh/119081	icddr,b International Centre for Diarrhoeal Disease Research,	Centers for Disease Control and Prevention	NA
**EPI_ISL_398369**	EPI1627858|3001289640_N8	Asia / Bangladesh	2019-08-	A/Bangladesh/119080	icddr,b International Centre for Diarrhoeal Disease Research,	Centers for Disease Control and Prevention	NA
**EPI_ISL_398394**	EPI1628057|3001289594_N8	Asia / Bangladesh	2019-07-	A/Bangladesh/4037/2	icddr,b International Centre for Diarrhoeal Disease Research,	Centers for Disease Control and Prevention	NA
**EPI_ISL_398396**	EPI1628073|3001289662_N8	Asia / Bangladesh	2019-08-	A/Bangladesh/8023/2	icddr,b International Centre for Diarrhoeal Disease Research,	Centers for Disease Control and Prevention	NA
**EPI_ISL_398530**	EPI1629116|3001289504_N8	Asia / Bangladesh	2019-07-	A/Bangladesh/1709/2	Institute of Epidemiology Disease Control and Research (IEDCR) &	Centers for Disease Control and Prevention	NA
**EPI_ISL_398557**	EPI1629332|3026053584_ZZ	North America / United States /	2019-11-	A/Illinois/35/2019	Illinois Department of Public Health-Carbondale	Centers for Disease Control and Prevention	NA
**EPI_ISL_398727**	EPI1630280|A/KANAGAWA/1	Asia / Japan	2019-09-	A/KANAGAWA/159/20	Kanagawa Prefectural Institute of Public Health	National Institute of Infectious Diseases	Kuwahara,Tomoko;Takashita,Emi;Fujisaki,Seiic
**EPI_ISL_398745**	EPI1630383|A/Singapore/MO	Asia / Singapore	2019-08-	A/Singapore/MOH013	Ministry of Health, Singapore	Ministry of Health, Singapore	Chen,B.;Zhou, Z.; Mak,T.M.; Cui,L; Lin,R.T.P.
**EPI_ISL_398810**	EPI1630900|3001289851_N8	North America / Nicaragua	2019-10-	A/Nicaragua/1406/201	Laboratorio de Virologia, Direccion de Microbiologia	Centers for Disease Control and Prevention	NA
**EPI_ISL_398812**	EPI1630915|3001289849_N8	North America / Nicaragua	2019-09-	A/Nicaragua/1398/201	Laboratorio de Virologia, Direccion de Microbiologia	Centers for Disease Control and Prevention	NA
**EPI_ISL_398827**	EPI1631035|3001289918_N8	North America / Canada	2019-09-	A/Ontario/RV2450/201	National Microbiology Laboratory, Public Health Agency of Canada	Centers for Disease Control and Prevention	NA
**EPI_ISL_398851**	EPI1631218|3001289599_N8	Asia / Bangladesh	2019-07-	A/Bangladesh/719070	icddr,b International Centre for Diarrhoeal Disease Research,	Centers for Disease Control and Prevention	NA
**EPI_ISL_399021**	EPI1632531|A/Singapore/GP2	Asia / Singapore	2019-10-	A/Singapore/GP2015/	Ministry of Health, Singapore	Ministry of Health, Singapore	Chen,B.;Zhou, Z.; Mak,T.M.; Cui,L; Lin,R.T.P.
**EPI_ISL_399769**	EPI1635187|A_ENG_9262063	Europe / United Kingdom /	2019-06-	A/England/92620633/	Microbiology Services Colindale, Public Health England	Microbiology Services Colindale, Public	Thompson,C.
**EPI_ISL_399770**	EPI1635189|A_ENG_9304053	Europe / United Kingdom /	2019-07-	A/England/93040538/	Microbiology Services Colindale, Public Health England	Microbiology Services Colindale, Public	Thompson,C.
**EPI_ISL_399777**	EPI1635201|A_ENG_319_201	Europe / United Kingdom /	2019-09-	A/England/319/2019	Microbiology Services Colindale, Public Health England	Microbiology Services Colindale, Public	Thompson,C.
**EPI_ISL_399788**	EPI1635277|A_ENG_9262063	Europe / United Kingdom /	2019-06-	A/England/92620632/	Microbiology Services Colindale, Public Health England	Microbiology Services Colindale, Public	Thompson,C.
**EPI_ISL_399789**	EPI1635285|A_ENG_9270043	Europe / United Kingdom /	2019-06-	A/England/92700431/	Microbiology Services Colindale, Public Health England	Microbiology Services Colindale, Public	Thompson,C.
**EPI_ISL_399804**	EPI1635405|A_ENG_318_201	Europe / United Kingdom /	2019-09-	A/England/318/2019	Microbiology Services Colindale, Public Health England	Microbiology Services Colindale, Public	Thompson,C.
**EPI_ISL_399813**	EPI1635477|A_ENG_1942205	Europe / United Kingdom /	2019-10-	A/England/194220533	Microbiology Services Colindale, Public Health England	Microbiology Services Colindale, Public	Thompson,C.
**EPI_ISL_399821**	EPI1635541|A_ENG_322_201	Europe / United Kingdom /	2019-10-	A/England/322/2019	Microbiology Services Colindale, Public Health England	Microbiology Services Colindale, Public	Thompson,C.
**EPI_ISL_399938**	EPI1635970|A/Czech	Europe / Czech Republic /	2019-09-	A/Czech	National Institute of Public Health	National Institute of Public Health (Czech	A,Nagy; M,Havlíčková; H,Jiřincová
**EPI_ISL_400014**	EPI1636430|3026053755_ZZ	North America / United States /	2019-11-	A/Mississippi/32/2019	Mississippi Public Health Laboratory	Centers for Disease Control and Prevention	NA
**EPI_ISL_400033**	EPI1636575|3001292056_N8	Asia / Bhutan	2019-07-	A/Bhutan/885/2019	Royal Centre for Disease Control	Centers for Disease Control and Prevention	NA
**EPI_ISL_400036**	EPI1636599|3001292067_N8	Asia / Bhutan	2019-09-	A/Bhutan/1095/2019	Royal Centre for Disease Control	Centers for Disease Control and Prevention	NA
**EPI_ISL_400291**	EPI1638598|A/Singapore/GP1	Asia / Singapore	2019-08-	A/Singapore/GP1559/	Ministry of Health, Singapore	Ministry of Health, Singapore	Chen,B.; Zhou, Z.; Mak,T.M.; Cui,L; Lin,R.T.P.
**EPI_ISL_400293**	EPI1638614|A/Singapore/GP1	Asia / Singapore	2019-09-	A/Singapore/GP1731/	Ministry of Health, Singapore	Ministry of Health, Singapore	Chen,B.; Zhou, Z.; Mak,T.M.; Cui,L; Lin,R.T.P.
**EPI_ISL_400294**	EPI1638622|A/Singapore/GP1	Asia / Singapore	2019-09-	A/Singapore/GP1793/	Ministry of Health, Singapore	Ministry of Health, Singapore	Chen,B.; Zhou, Z.; Mak,T.M.; Cui,L; Lin,R.T.P.
**EPI_ISL_400295**	EPI1638630|A/Singapore/TT0	Asia / Singapore	2019-08-	A/Singapore/TT0662/2	Ministry of Health, Singapore	Ministry of Health, Singapore	Chen,B.; Zhou, Z.; Mak,T.M.; Cui,L; Lin,R.T.P.
**EPI_ISL_400296**	EPI1638638|A/Singapore/TT0	Asia / Singapore	2019-08-	A/Singapore/TT0710/2	Ministry of Health, Singapore	Ministry of Health, Singapore	Chen,B.; Zhou, Z.; Mak,T.M.; Cui,L; Lin,R.T.P.

**EPI_ISL_400298**	EPI1638654|A/Singapore/NUH	Asia / Singapore	2019-08-	A/Singapore/NUH002	Ministry of Health, Singapore	Ministry of Health, Singapore	Chen,B.; Zhou, Z.; Mak,T.M.; Cui,L; Lin,R.T.P.
**EPI_ISL_400301**	EPI1638678|A/Singapore/NTF	Asia / Singapore	2019-07-	A/Singapore/NTF0053	Ministry of Health, Singapore	Ministry of Health, Singapore	Chen,B.; Zhou, Z.; Mak,T.M.; Cui,L; Lin,R.T.P.
**EPI_ISL_400302**	EPI1638685|A/Singapore/TT0	Asia / Singapore	2019-08-	A/Singapore/TT0665/2	Ministry of Health, Singapore	Ministry of Health, Singapore	Chen,B.; Zhou, Z.; Mak,T.M.; Cui,L; Lin,R.T.P.
**EPI_ISL_400303**	EPI1638693|A/Singapore/TT0	Asia / Singapore	2019-08-	A/Singapore/TT0651/2	Ministry of Health, Singapore	Ministry of Health, Singapore	Chen,B.; Zhou, Z.; Mak,T.M.; Cui,L; Lin,R.T.P.
**EPI_ISL_400308**	EPI1638733|A/Singapore/TT0	Asia / Singapore	2019-08-	A/Singapore/TT0679/2	Ministry of Health, Singapore	Ministry of Health, Singapore	Chen,B.; Zhou, Z.; Mak,T.M.; Cui,L; Lin,R.T.P.
**EPI_ISL_400313**	EPI1638773|A/Singapore/TT0	Asia / Singapore	2019-09-	A/Singapore/TT0767/2	Ministry of Health, Singapore	Ministry of Health, Singapore	Chen,B.; Zhou, Z.; Mak,T.M.; Cui,L; Lin,R.T.P.
**EPI_ISL_400343**	EPI1638901|A/Centre/2559/20	Europe / France / Region	2019-11-	A/Centre/2559/2019	Institut Pasteur	Institut Pasteur	NA
**EPI_ISL_400525**	EPI1639623|A/Cardiff/9403/20	Europe / United Kingdom / City	2019-12-	A/Cardiff/9403/2019	Public Health Wales Microbiology Cardiff	Public Health Wales Microbiology Cardiff	NA
**EPI_ISL_400553**	EPI1639993|A/KANAGAWA/Z	Asia / Japan	2019-11-	A/KANAGAWA/ZC190	National Institute of Infectious Diseases (NIID)	National Institute of Infectious Diseases	Kuwahara,Tomoko;Takashita,Emi;Fujisaki,Seiic
**EPI_ISL_400565**	EPI1640029|A/KANAGAWA/1	Asia / Japan	2019-10-	A/KANAGAWA/162/20	Kanagawa Prefectural Institute of Public Health	National Institute of Infectious Diseases	Kuwahara,Tomoko;Takashita,Emi;Fujisaki,Seiic
**EPI_ISL_400577**	EPI1640077|A/SAPPORO/58/	Asia / Japan	2019-10-	A/SAPPORO/58/2019	Sapporo City Institute of Public Health	National Institute of Infectious Diseases	Kuwahara,Tomoko;Takashita,Emi;Fujisaki,Seiic
**EPI_ISL_400582**	EPI1640087|A/YOKOHAMA/2	Asia / Japan	2019-11-	A/YOKOHAMA/210/20	Yokohama City Institute of Public Health.	National Institute of Infectious Diseases	Kuwahara,Tomoko;Takashita,Emi;Fujisaki,Seiic
**EPI_ISL_400583**	EPI1640095|A/NAGANO/2599	Asia / Japan	2019-09-	A/NAGANO/2599/201	Nagano Environmental Conservation Research Institute	National Institute of Infectious Diseases	Kuwahara,Tomoko;Takashita,Emi;Fujisaki,Seiic
**EPI_ISL_400618**	EPI1640359|A/HYOGO/1023/2	Asia / Japan	2019-09-	A/HYOGO/1023/2019	Hyogo Prefectural Institute of Public Health and Consumer Sciences	National Institute of Infectious Diseases	Kuwahara,Tomoko;Takashita,Emi;Fujisaki,Seiic
**EPI_ISL_400634**	EPI1640404|A/SAITAMA/176/	Asia / Japan	2019-08-	A/SAITAMA/176/2019	Saitama Institute of Public Health	National Institute of Infectious Diseases	Kuwahara,Tomoko;Takashita,Emi;Fujisaki,Seiic
**EPI_ISL_400745**	EPI1641074|A/Denmark/3255/	Europe / Denmark	2019-10-	A/Denmark/3255/2019	Statens Serum Institute	Statens Serum Institute	Trebbien, R.
**EPI_ISL_400751**	EPI1641092|A/Denmark/3260/	Europe / Denmark	2019-10-	A/Denmark/3260/2019	Statens Serum Institute	Statens Serum Institute	Trebbien, R.
**EPI_ISL_400773**	EPI1641257|3001289973_N8	North America / Trinidad and	2019-08-	A/Trinidad/4378/2019	Caribbean Epidemiology Center	Centers for Disease Control and Prevention	NA
**EPI_ISL_400775**	EPI1641273|3001292267_N8	South America / Peru	2019-09-	A/Peru/3519/2019	Laboratorio de Referencia Nacional Virus Respiratorios, Instituto	Centers for Disease Control and Prevention	NA
**EPI_ISL_400790**	EPI1641381|3001289951_N8	Africa / Togo	2019-09-	A/Togo/1306/2019	Institute National D'Hygiene	Centers for Disease Control and Prevention	NA
**EPI_ISL_400799**	EPI1641446|3001292351_N8	Asia / Bangladesh	2019-10-	A/Bangladesh/328/20	Institute of Epidemiology Disease Control and Research (IEDCR) &	Centers for Disease Control and Prevention	NA
**EPI_ISL_400801**	EPI1641462|3001292352_N8	Asia / Bangladesh	2019-10-	A/Bangladesh/638/20	Institute of Epidemiology Disease Control and Research (IEDCR) &	Centers for Disease Control and Prevention	NA
**EPI_ISL_400802**	EPI1641470|3001292348_N8	Asia / Bangladesh	2019-10-	A/Bangladesh/1123/2	Institute of Epidemiology Disease Control and Research (IEDCR) &	Centers for Disease Control and Prevention	NA
**EPI_ISL_400804**	EPI1641482|3001292465_N8	North America / Jamaica	2019-10-	A/Jamaica/70447/201	University of the West Indies	Centers for Disease Control and Prevention	NA
**EPI_ISL_400819**	EPI1641573|3001292354_N8	Asia / Bangladesh	2019-10-	A/Bangladesh/1126/2	Institute of Epidemiology Disease Control and Research (IEDCR) &	Centers for Disease Control and Prevention	NA
**EPI_ISL_400834**	EPI1641679|3001292456_N8	North America / Jamaica	2019-10-	A/Jamaica/60381/201	University of the West Indies	Centers for Disease Control and Prevention	NA
**EPI_ISL_400841**	EPI1641721|3001292441_N8	North America / Jamaica	2019-10-	A/Jamaica/60904/201	University of the West Indies	Centers for Disease Control and Prevention	NA
**EPI_ISL_400864**	EPI1642786|3001292432_N8	North America / Jamaica	2019-11-	A/Jamaica/30235/201	University of the West Indies	Centers for Disease Control and Prevention	NA
**EPI_ISL_400869**	EPI1641889|3026053933_ZZ	North America / United States /	2019-11-	A/Idaho/40/2019	State of Idaho Bureau of Laboratories	Centers for Disease Control and Prevention	NA
**EPI_ISL_400870**	EPI1641897|3026053884_ZZ	North America / United States /	2019-11-	A/New York/54/2019	New York State Department of Health	Centers for Disease Control and Prevention	NA
**EPI_ISL_401004**	EPI1642975|3001292289_N8	South America / Peru	2019-10-	A/Peru/7019/2019	Laboratorio de Referencia Nacional Virus Respiratorios, Instituto	Centers for Disease Control and Prevention	NA
**EPI_ISL_401010**	EPI1643038|3001292270_N8	South America / Peru	2019-09-	A/Peru/6019/2019	Laboratorio de Referencia Nacional Virus Respiratorios, Instituto	Centers for Disease Control and Prevention	NA
**EPI_ISL_401015**	EPI1643154|3001292443_N8	North America / Jamaica	2019-10-	A/Jamaica/60945/201	University of the West Indies	Centers for Disease Control and Prevention	NA
**EPI_ISL_401016**	EPI1643162|3001289512_N8	Asia / Bangladesh	2019-06-	A/Bangladesh/1729/2	Institute of Epidemiology Disease Control and Research (IEDCR) &	Centers for Disease Control and Prevention	NA
**EPI_ISL_401458**	EPI1646142|A/Singapore/EN0	Asia / Singapore	2019-11-	A/Singapore/EN0858/	Ministry of Health, Singapore	Ministry of Health, Singapore	Chen,B.;Zhou, Z.; Mak,T.M.; Cui,L; Lin,R.T.P.
**EPI_ISL_401464**	EPI1646190|A/Singapore/GP2	Asia / Singapore	2019-12-	A/Singapore/GP2299/	Ministry of Health, Singapore	Ministry of Health, Singapore	Chen,B.;Zhou, Z.; Mak,T.M.; Cui,L; Lin,R.T.P.
**EPI_ISL_401466**	EPI1646206|A/Singapore/KK2	Asia / Singapore	2019-11-	A/Singapore/KK2585/	Ministry of Health, Singapore	Ministry of Health, Singapore	Chen,B.;Zhou, Z.; Mak,T.M.; Cui,L; Lin,R.T.P.
**EPI_ISL_401504**	EPI1646504|A/Singapore/SGH	Asia / Singapore	2019-12-	A/Singapore/SGH062	Ministry of Health, Singapore	Ministry of Health, Singapore	Chen,B.;Zhou, Z.; Mak,T.M.; Cui,L; Lin,R.T.P.
**EPI_ISL_401505**	EPI1646517|A/Singapore/SGH	Asia / Singapore	2019-12-	A/Singapore/SGH066	Ministry of Health, Singapore	Ministry of Health, Singapore	Chen,B.;Zhou, Z.; Mak,T.M.; Cui,L; Lin,R.T.P.
**EPI_ISL_401567**	EPI1646725|61-	Asia / Oman	2019-10-	A/Ibra/6555/2019	Central Public Health Laboratory, Ministry of Health	Crick Worldwide Influenza Centre	NA
**EPI_ISL_401573**	EPI1646731|28-	Asia / Oman	2019-10-	A/Ibri/6789/2019	Central Public Health Laboratory, Ministry of Health	Crick Worldwide Influenza Centre	NA
**EPI_ISL_401595**	EPI1646753|56-	Asia / Oman	2019-10-	A/Muscat/6830/2019	Central Public Health Laboratory, Ministry of Health	Crick Worldwide Influenza Centre	NA
**EPI_ISL_401607**	EPI1646765|75-	Asia / Oman	2019-09-	A/Salalah/5662/2019	Central Public Health Laboratory, Ministry of Health	Crick Worldwide Influenza Centre	NA
**EPI_ISL_401623**	EPI1646781|55-	Asia / Oman	2019-10-	A/Sur/6859/2019	Central Public Health Laboratory, Ministry of Health	Crick Worldwide Influenza Centre	NA
**EPI_ISL_401710**	EPI1647046|62-A_Qatar_10-	Asia / Qatar	2019-08-	A/Qatar/10-VI-19-	Supreme Health Council	Crick Worldwide Influenza Centre	NA
**EPI_ISL_401719**	EPI1647064|64-A_Qatar_13-	Asia / Qatar	2019-08-	A/Qatar/13-VI-19-	Supreme Health Council	Crick Worldwide Influenza Centre	NA
**EPI_ISL_401724**	EPI1647073|51-A_Qatar_16-	Asia / Qatar	2019-08-	A/Qatar/16-VI-19-	Supreme Health Council	Crick Worldwide Influenza Centre	NA
**EPI_ISL_401769**	EPI1647161|63-A_Qatar_13-	Asia / Qatar	2019-08-	A/Qatar/13-VI-19-	Supreme Health Council	Crick Worldwide Influenza Centre	NA
**EPI_ISL_401855**	EPI1647419|N10034086.4	Oceania / Australia / South	2019-11-	A/South	Institute of Medical and Veterinary Science (IMVS)	WHO Collaborating Centre for Reference	Deng,Y-M; Iannello,P; Lau,H; Todd,A;
**EPI_ISL_401860**	EPI1647434|N10034091.4	Oceania / Australia / South	2019-11-	A/South	Institute of Medical and Veterinary Science (IMVS)	WHO Collaborating Centre for Reference	Deng,Y-M; Iannello,P; Lau,H; Todd,A;
**EPI_ISL_401865**	EPI1647449|N10034096.4	Oceania / Australia / Victoria /	2019-11-	A/Victoria/2576/2019	Alfred Hospital	WHO Collaborating Centre for Reference	Deng,Y-M; Iannello,P; Lau,H; Todd,A;
**EPI_ISL_401869**	EPI1647461|N10034100.4	Oceania / Australia / Victoria /	2019-11-	A/Victoria/2573/2019	Alfred Hospital	WHO Collaborating Centre for Reference	Deng,Y-M; Iannello,P; Lau,H; Todd,A;
**EPI_ISL_401871**	EPI1647467|N10034102.4	Oceania / Australia / South	2019-11-	A/South	Institute of Medical and Veterinary Science (IMVS)	WHO Collaborating Centre for Reference	Deng,Y-M; Iannello,P; Lau,H; Todd,A;
**EPI_ISL_402017**	EPI1647805|N10033156.4	Asia / Malaysia	2019-10-	A/Malaysia/RP2256/20	Institut Penyelidikan Perubatan	WHO Collaborating Centre for Reference	Deng,Y-M; Iannello,P; Lau,H; Todd,A;
**EPI_ISL_402024**	EPI1647826|N10033163.4	Oceania / Australia / Victoria /	2019-10-	A/Victoria/2539/2019	Alfred Hospital	WHO Collaborating Centre for Reference	Deng,Y-M; Iannello,P; Lau,H; Todd,A;
**EPI_ISL_402057**	EPI1647924|N10032670.4	Oceania / Australia / Victoria /	2019-09-	A/Victoria/252/2019	Victorian Infectious Diseases Reference Laboratory	WHO Collaborating Centre for Reference	Deng,Y-M; Iannello,P; Lau,H; Todd,A;
**EPI_ISL_402058**	EPI1647927|N10032671.4	Oceania / Australia / Victoria /	2019-09-	A/Victoria/253/2019	Victorian Infectious Diseases Reference Laboratory	WHO Collaborating Centre for Reference	Deng,Y-M; Iannello,P; Lau,H; Todd,A;
**EPI_ISL_402063**	EPI1647939|N10032676.4	Oceania / Australia / Victoria /	2019-09-	A/Victoria/256/2019	Victorian Infectious Diseases Reference Laboratory	WHO Collaborating Centre for Reference	Deng,Y-M; Iannello,P; Lau,H; Todd,A;
**EPI_ISL_402156**	EPI1648334|2020-CX0051.4	Asia / China / Shandong	2019-12-	A/Shandong-	WHO Chinese National Influenza Center	WHO Chinese National Influenza Center	Xiaoxu,Zeng;Xiyan,Li;Weijuan,Huang;Lei,Yang;D
**EPI_ISL_402159**	EPI1648343|2020-CX0048.4	Asia / China / Jilin	2019-12-	A/Jilin-	WHO Chinese National Influenza Center	WHO Chinese National Influenza Center	Xiaoxu,Zeng;Xiyan,Li;Weijuan,Huang;Lei,Yang;D
**EPI_ISL_402161**	EPI1648349|2020-CX0046.4	Asia / China / Henan	2019-12-	A/Henan-	WHO Chinese National Influenza Center	WHO Chinese National Influenza Center	Xiaoxu,Zeng;Xiyan,Li;Weijuan,Huang;Lei,Yang;D
**EPI_ISL_402168**	EPI1648370|2020-CX0038.4	Asia / China / Hebei	2019-11-	A/Hebei-	WHO Chinese National Influenza Center	WHO Chinese National Influenza Center	Xiaoxu,Zeng;Xiyan,Li;Weijuan,Huang;Lei,Yang;D
**EPI_ISL_402171**	EPI1648379|2020-CX0035.4	Asia / China / Shandong	2019-12-	A/Shandong-	WHO Chinese National Influenza Center	WHO Chinese National Influenza Center	Xiaoxu,Zeng;Xiyan,Li;Weijuan,Huang;Lei,Yang;D
**EPI_ISL_402174**	EPI1648388|2020-CX0032.4	Asia / China	2019-12-	A/Xinjiang-	WHO Chinese National Influenza Center	WHO Chinese National Influenza Center	Xiaoxu,Zeng;Xiyan,Li;Weijuan,Huang;Lei,Yang;D
**EPI_ISL_402204**	EPI1648478|2019-CX2845.4	Asia / China / Hubei	2019-11-	A/Hubei-	WHO Chinese National Influenza Center	WHO Chinese National Influenza Center	Xiaoxu,Zeng;Xiyan,Li;Weijuan,Huang;Lei,Yang;D
**EPI_ISL_402209**	EPI1648493|2019-CX2840.4	Asia / China / Zhejiang	2019-11-	A/Zhejiang-	WHO Chinese National Influenza Center	WHO Chinese National Influenza Center	Xiaoxu,Zeng;Xiyan,Li;Weijuan,Huang;Lei,Yang;D
**EPI_ISL_402257**	EPI1648637|2019-CX2792.4	Asia / China / Sichuan	2019-10-	A/Sichuan-	WHO Chinese National Influenza Center	WHO Chinese National Influenza Center	Xiaoxu,Zeng;Xiyan,Li;Weijuan,Huang;Lei,Yang;D
**EPI_ISL_402258**	EPI1648640|2019-CX2791.4	Asia / China / Hunan	2019-10-	A/Hunan-	WHO Chinese National Influenza Center	WHO Chinese National Influenza Center	Xiaoxu,Zeng;Xiyan,Li;Weijuan,Huang;Lei,Yang;D

**EPI_ISL_402259**	EPI1648643|2019-CX2790.4	Asia / China / Chongqing	2019-10-	A/Chongqing-	WHO Chinese National Influenza Center	WHO Chinese National Influenza Center	Xiaoxu,Zeng;Xiyan,Li;Weijuan,Huang;Lei,Yang;D
**EPI_ISL_402264**	EPI1648658|2019-CX2784.4	Asia / China / Xinjiang	2019-11-	A/Xinjiang-	WHO Chinese National Influenza Center	WHO Chinese National Influenza Center	Xiaoxu,Zeng;Xiyan,Li;Weijuan,Huang;Lei,Yang;D
**EPI_ISL_402291**	EPI1648739|2019-CX2734.4	Asia / China / Guangdong	2019-10-	A/Guangdong-	WHO Chinese National Influenza Center	WHO Chinese National Influenza Center	Xiaoxu,Zeng;Xiyan,Li;Weijuan,Huang;Lei,Yang;D
**EPI_ISL_402293**	EPI1648745|2019-CX2732.4	Asia / China / Guizhou	2019-10-	A/Guizhou-	WHO Chinese National Influenza Center	WHO Chinese National Influenza Center	Xiaoxu,Zeng;Xiyan,Li;Weijuan,Huang;Lei,Yang;D
**EPI_ISL_402294**	EPI1648748|2019-CX2731.4	Asia / China / Zhejiang	2019-10-	A/Zhejiang-	WHO Chinese National Influenza Center	WHO Chinese National Influenza Center	Xiaoxu,Zeng;Xiyan,Li;Weijuan,Huang;Lei,Yang;D
**EPI_ISL_402351**	EPI1648919|2019-CX2583.4	Asia / China / Jiangxi	2019-09-	A/Jiangxi-	WHO Chinese National Influenza Center	WHO Chinese National Influenza Center	Xiaoxu,Zeng;Xiyan,Li;Weijuan,Huang;Lei,Yang;D
**EPI_ISL_402357**	EPI1648937|2019-CX2576.4	Asia / China / Hunan	2019-09-	A/Hunan-	WHO Chinese National Influenza Center	WHO Chinese National Influenza Center	Xiaoxu,Zeng;Xiyan,Li;Weijuan,Huang;Lei,Yang;D
**EPI_ISL_402361**	EPI1648949|2019-CX2572.4	Asia / China	2019-09-	A/Guangdong-	WHO Chinese National Influenza Center	WHO Chinese National Influenza Center	Xiaoxu,Zeng;Xiyan,Li;Weijuan,Huang;Lei,Yang;D
**EPI_ISL_402363**	EPI1648955|2019-CX2570.4	Asia / China / Shanghai	2019-09-	A/Shanghai-	WHO Chinese National Influenza Center	WHO Chinese National Influenza Center	Xiaoxu,Zeng;Xiyan,Li;Weijuan,Huang;Lei,Yang;D
**EPI_ISL_402366**	EPI1648964|2019-CX2567.4	Asia / China / Sichuan	2019-09-	A/Sichuan-	WHO Chinese National Influenza Center	WHO Chinese National Influenza Center	Xiaoxu,Zeng;Xiyan,Li;Weijuan,Huang;Lei,Yang;D
**EPI_ISL_402374**	EPI1648988|2019-CX2559.4	Asia / China / Sichuan	2019-09-	A/Sichuan-	WHO Chinese National Influenza Center	WHO Chinese National Influenza Center	Xiaoxu,Zeng;Xiyan,Li;Weijuan,Huang;Lei,Yang;D
**EPI_ISL_402399**	EPI1649104|3001292514_N8	South America / Argentina	2019-11-	A/Argentina/2852/201	Instituto Nacional de Enfermedades Infecciosas	Centers for Disease Control and Prevention	NA
**EPI_ISL_402400**	EPI1649112|3001292510_N8	South America / Argentina	2019-11-	A/Argentina/2840/201	Instituto Nacional de Enfermedades Infecciosas	Centers for Disease Control and Prevention	NA
**EPI_ISL_402406**	EPI1649160|3026054042_ZZ	North America / United States /	2019-11-	A/Michigan/454/2019	Michigan Department of Community Health	Centers for Disease Control and Prevention	NA
**EPI_ISL_402407**	EPI1649168|3001292403_N8	Africa / Niger	2019-09-	A/Niger/7171/2019	Centre de Recherche Medicale et Sanitaire (CERMES)	Centers for Disease Control and Prevention	NA
**EPI_ISL_402415**	EPI1649218|3001292526_N8	Asia / Philippines	2019-09-	A/Philippines/1078/20	Research Institute for Tropical Medicine, Virology Department	Centers for Disease Control and Prevention	NA
**EPI_ISL_402419**	EPI1649248|3001292542_N8	Asia / Philippines	2019-09-	A/Philippines/1343/20	Research Institute for Tropical Medicine, Virology Department	Centers for Disease Control and Prevention	NA
**EPI_ISL_402426**	EPI1649304|3001292558_N8	Asia / Philippines	2019-10-	A/Philippines/1452/20	Research Institute for Tropical Medicine, Virology Department	Centers for Disease Control and Prevention	NA
**EPI_ISL_402434**	EPI1649367|3001292478_N8	South America / Brazil	2019-08-	A/Ituverava/19180886-	Instituto Adolfo Lutz	Centers for Disease Control and Prevention	NA
**EPI_ISL_402450**	EPI1649494|3001292344_N8	Asia / Bangladesh	2019-10-	A/Bangladesh/119100	icddr,b International Centre for Diarrhoeal Disease Research,	Centers for Disease Control and Prevention	NA
**EPI_ISL_402452**	EPI1649510|3001292342_N8	Asia / Bangladesh	2019-10-	A/Bangladesh/719101	icddr,b International Centre for Diarrhoeal Disease Research,	Centers for Disease Control and Prevention	NA
**EPI_ISL_402453**	EPI1649518|3001292339_N8	Asia / Bangladesh	2019-09-	A/Bangladesh/119091	icddr,b International Centre for Diarrhoeal Disease Research,	Centers for Disease Control and Prevention	NA
**EPI_ISL_402454**	EPI1649526|3001292338_N8	Asia / Bangladesh	2019-09-	A/Bangladesh/119091	icddr,b International Centre for Diarrhoeal Disease Research,	Centers for Disease Control and Prevention	NA
**EPI_ISL_402460**	EPI1649574|3001292286_N8	South America / Peru	2019-10-	A/Peru/555919/2019	Laboratorio de Referencia Nacional Virus Respiratorios, Instituto	Centers for Disease Control and Prevention	NA
**EPI_ISL_402461**	EPI1649582|3001292355_N8	Asia / Bangladesh	2019-10-	A/Bangladesh/18-	Institute of Epidemiology Disease Control and Research (IEDCR) &	Centers for Disease Control and Prevention	NA
**EPI_ISL_402462**	EPI1649590|3001292347_N8	Asia / Bangladesh	2019-10-	A/Bangladesh/119101	icddr,b International Centre for Diarrhoeal Disease Research,	Centers for Disease Control and Prevention	NA
**EPI_ISL_402464**	EPI1649606|3001292269_N8	South America / Peru	2019-09-	A/Peru/90704619/201	Laboratorio de Referencia Nacional Virus Respiratorios, Instituto	Centers for Disease Control and Prevention	NA
**EPI_ISL_402491**	EPI1649829|3001292618_N8	South America / Ecuador	2019-11-	A/Ecuador/4321/2019	INSPI	Centers for Disease Control and Prevention	NA
**EPI_ISL_402494**	EPI1649846|3026054181_ZZ	North America / United States /	2019-12-	A/Wisconsin/585/2019	Wisconsin State Laboratory of Hygiene	Centers for Disease Control and Prevention	NA
**EPI_ISL_402504**	EPI1649923|3001292412_N8	Africa / Niger	2019-10-	A/Niger/7268/2019	Centre de Recherche Medicale et Sanitaire (CERMES)	Centers for Disease Control and Prevention	NA
**EPI_ISL_402512**	EPI1649987|3026054143_ZZ	North America / United States /	2019-12-	A/Ohio/35/2019	Ohio Department of Health Laboratories	Centers for Disease Control and Prevention	NA
**EPI_ISL_402514**	EPI1650004|3001292493_N8	South America / Brazil	2019-10-	A/Marilia/1344855-	Instituto Adolfo Lutz	Centers for Disease Control and Prevention	NA
**EPI_ISL_402522**	EPI1650069|3001292642_N8	South America / Chile	2019-12-	A/Chile/3542/2019	Instituto de Salud Publica de Chile	Centers for Disease Control and Prevention	NA
**EPI_ISL_402769**	EPI1651935|3001292419_N8	Africa / Niger	2019-12-	A/Niger/7296/2019	Centre de Recherche Medicale et Sanitaire (CERMES)	Centers for Disease Control and Prevention	NA
**EPI_ISL_402775**	EPI1651981|3001292406_N8	Africa / Niger	2019-09-	A/Niger/7202/2019	Centre de Recherche Medicale et Sanitaire (CERMES)	Centers for Disease Control and Prevention	NA
**EPI_ISL_402776**	EPI1651989|3001292407_N8	Africa / Niger	2019-09-	A/Niger/7204/2019	Centre de Recherche Medicale et Sanitaire (CERMES)	Centers for Disease Control and Prevention	NA
**EPI_ISL_403003**	EPI1653681|A/LaReunion/212	Europe / France	2019-08-	A/LaReunion/2125/20	CNR Virus des Infections Respiratoires - France SUD	CNR Virus des Infections Respiratoires -	NA
**EPI_ISL_403071**	EPI1654062|A/Colorado/9861/	North America / United States /	2019-11-	A/Colorado/9861/2019	U.S. Air Force School of Aerospace Medicine	U.S. Air Force School of Aerospace	Gruner, W.E.; Fries, A.C.; Garrett, C.M.; Powell,
**EPI_ISL_403094**	EPI1654243|A/New	North America / United States /	2019-10-	A/New	U.S. Air Force School of Aerospace Medicine	U.S. Air Force School of Aerospace	Gruner, W.E.; Fries, A.C.; Garrett, C.M.; Powell,
**EPI_ISL_403279**	EPI1655541|44-	Asia / Iran, Islamic Republic of	2019-11-	A/Iran/200265/2019	Tehran University of Medical Sciences	Crick Worldwide Influenza Centre	NA
**EPI_ISL_403676**	EPI1656917|A/Concepcion/90	South America / Chile /	2019-09-	A/Concepcion/90512/	Instituto de Salud Publica de Chile	Instituto de Salud Publica de Chile	Tognarelli, J.; Lagos, J.; Arata, L.; Fasce, R.;
**EPI_ISL_403677**	EPI1656925|A/Iquique/92314/	South America / Chile / Iquique	2019-09-	A/Iquique/92314/2019	Instituto de Salud Publica de Chile	Instituto de Salud Publica de Chile	Tognarelli, J.; Lagos, J.; Arata, L.; Fasce, R.;
**EPI_ISL_404036**	EPI1658575|N10032821.4	Oceania / Australia / Victoria /	2019-10-	A/Victoria/2557/2019	Alfred Hospital	WHO Collaborating Centre for Reference	Deng,Y-M; Iannello,P; Lau,H; Todd,A;
**EPI_ISL_404039**	EPI1658584|N10032824.4	Oceania / Australia / Victoria /	2019-10-	A/Victoria/2548/2019	Alfred Hospital	WHO Collaborating Centre for Reference	Deng,Y-M; Iannello,P; Lau,H; Todd,A;
**EPI_ISL_404044**	EPI1658599|N10032829.4	Oceania / Australia / Victoria /	2019-10-	A/Victoria/2554/2019	Alfred Hospital	WHO Collaborating Centre for Reference	Deng,Y-M; Iannello,P; Lau,H; Todd,A;
**EPI_ISL_404048**	EPI1658611|N10032833.4	Oceania / Australia / Victoria /	2019-10-	A/Victoria/2562/2019	Alfred Hospital	WHO Collaborating Centre for Reference	Deng,Y-M; Iannello,P; Lau,H; Todd,A;
**EPI_ISL_404049**	EPI1658614|N10032834.4	Oceania / Australia / Victoria /	2019-10-	A/Victoria/2563/2019	Alfred Hospital	WHO Collaborating Centre for Reference	Deng,Y-M; Iannello,P; Lau,H; Todd,A;
**EPI_ISL_404051**	EPI1658620|N10032836.4	Oceania / Australia / Victoria /	2019-11-	A/Victoria/2856/2019	Royal Chidrens Hospital	WHO Collaborating Centre for Reference	Deng,Y-M; Iannello,P; Lau,H; Todd,A;
**EPI_ISL_404054**	EPI1658629|N10032839.4	Oceania / Australia / Victoria /	2019-09-	A/Victoria/2510/2019	Alfred Hospital	WHO Collaborating Centre for Reference	Deng,Y-M; Iannello,P; Lau,H; Todd,A;
**EPI_ISL_404055**	EPI1658632|N10032840.4	Oceania / Australia / Victoria /	2019-10-	A/Victoria/264/2019	Victorian Infectious Diseases Reference Laboratory	WHO Collaborating Centre for Reference	Deng,Y-M; Iannello,P; Lau,H; Todd,A;
**EPI_ISL_404076**	EPI1658695|N10032861.4	Oceania / Australia / Northern	2019-10-	A/Darwin/726/2019	Influenza Surveillance Centre for Disease Control	WHO Collaborating Centre for Reference	Deng,Y-M; Iannello,P; Lau,H; Todd,A;
**EPI_ISL_404127**	EPI1658812|S10032404.4	Oceania / Australia / Ashmore	2019-10-	A/Brisbane/141/2019	Queensland Health Forensic and Scientific Services	WHO Collaborating Centre for Reference	Deng,Y-M; Iannello,P; Lau,H; Todd,A;
**EPI_ISL_404129**	EPI1658818|S10032406.4	Oceania / Australia / Avalon	2019-10-	A/Sydney/1234/2019	Institute of Medical and Veterinary Science (IMVS)	WHO Collaborating Centre for Reference	Deng,Y-M; Iannello,P; Lau,H; Todd,A;
**EPI_ISL_404212**	EPI1659594|Infl_19-	Europe / Sweden / Vastra	2019-12-	A/Sweden/148/2019	Public Health Agency of Sweden	Public Health Agency of Sweden	NA
**EPI_ISL_404317**	EPI1660186|A/New	North America / United States /	2019-11-	A/New	U.S. Air Force School of Aerospace Medicine	U.S. Air Force School of Aerospace	Gruner, W.E.; Fries, A.C.; Garrett, C.M.; Powell,
**EPI_ISL_404534**	EPI1661809|3001292635_N8	South America / Chile	2019-11-	A/Chile/5905/2019	Instituto de Salud Publica de Chile	Centers for Disease Control and Prevention	NA
**EPI_ISL_404536**	EPI1661825|3001292633_N8	South America / Chile	2019-10-	A/Chile/2585/2019	Instituto de Salud Publica de Chile	Centers for Disease Control and Prevention	NA
**EPI_ISL_404584**	EPI1662195|3001292701_N8	Asia / United Arab Emirates	2019-11-	A/Abu Dhabi/100/2019	Shaikh Khalifa Medical City	Centers for Disease Control and Prevention	NA
**EPI_ISL_404600**	EPI1662309|3026054427_ZZ	North America / United States /	2019-12-	A/Alaska/59/2019	Alaska State Virology Lab	Centers for Disease Control and Prevention	NA
**EPI_ISL_404605**	EPI1662347|3026054292_ZZ	North America / United States /	2019-12-	A/Nevada/46/2019	Southern Nevada Public Health Lab	Centers for Disease Control and Prevention	NA
**EPI_ISL_404630**	EPI1662538|3001292640_N8	South America / Chile	2019-11-	A/Chile/1978/2019	Instituto de Salud Publica de Chile	Centers for Disease Control and Prevention	NA
**EPI_ISL_404631**	EPI1662546|3001292639_N8	South America / Chile	2019-11-	A/Chile/1699/2019	Instituto de Salud Publica de Chile	Centers for Disease Control and Prevention	NA
**EPI_ISL_404633**	EPI1662562|3001292637_N8	South America / Chile	2019-11-	A/Chile/7868/2019	Instituto de Salud Publica de Chile	Centers for Disease Control and Prevention	NA
**EPI_ISL_404634**	EPI1662570|3001292636_N8	South America / Chile	2019-11-	A/Chile/7066/2019	Instituto de Salud Publica de Chile	Centers for Disease Control and Prevention	NA
**EPI_ISL_404740**	EPI1663390|3001292772_N8	Asia / India	2019-08-	A/India/2260/2019	National Institute of Virology	Centers for Disease Control and Prevention	NA
**EPI_ISL_405102**	EPI1665594|A_ENG_334_201	Europe / United Kingdom /	2019-10-	A/England/334/2019	Microbiology Services Colindale, Public Health England	Microbiology Services Colindale, Public	Thompson,C.
**EPI_ISL_405103**	EPI1665602|A_ENG_357_201	Europe / United Kingdom /	2019-10-	A/England/357/2019	Microbiology Services Colindale, Public Health England	Microbiology Services Colindale, Public	Thompson,C.
**EPI_ISL_405259**	EPI1666850|A_ENG_488_201	Europe / United Kingdom /	2019-11-	A/England/488/2019	Microbiology Services Colindale, Public Health England	Microbiology Services Colindale, Public	Thompson,C.

**EPI_ISL_405389**	EPI1667589|A/Czech	Europe / Czech Republic /	2019-11-	A/Czech	National Institute of Public Health	State Veterinary Institute Prague	Nagy,A;Trnka,D;Jirincova,H
**EPI_ISL_405417**	EPI1667698|A/Guam/10291/2	Oceania / Guam	2019-12-	A/Guam/10291/2019	U.S. Air Force School of Aerospace Medicine	U.S. Air Force School of Aerospace Medicine	Gruner, W.E.; Fries, A.C.; Garrett, C.M.; Powell,
**EPI_ISL_405435**	EPI1667833|A/Mississippi/103	North America / United States /	2019-12-	A/Mississippi/10309/20	U.S. Air Force School of Aerospace Medicine	U.S. Air Force School of Aerospace	Gruner, W.E.; Fries, A.C.; Garrett, C.M.; Powell,
**EPI_ISL_405459**	EPI1668015|A/South	Asia / Korea, Republic of	2019-12-	A/South	U.S. Air Force School of Aerospace Medicine	U.S. Air Force School of Aerospace	Gruner, W.E.; Fries, A.C.; Garrett, C.M.; Powell,
**EPI_ISL_405726**	EPI1668984|3026054413_ZZ	North America / United States /	2019-12-	A/District Of	DC Public Health Lab	Centers for Disease Control and Prevention	NA
**EPI_ISL_405850**	EPI1669741|A/TOKYO/19301/	Asia / Japan	2019-10-	A/TOKYO/19301/2019	Tokyo Metropolitan Institute of Public Health	National Institute of Infectious Diseases	Kuwahara,Tomoko;Takashita,Emi;Fujisaki,Seiic
**EPI_ISL_405853**	EPI1669747|A/YOKOHAMA/2	Asia / Japan	2019-11-	A/YOKOHAMA/237/20	Yokohama City Institute of Public Health.	National Institute of Infectious Diseases	Kuwahara,Tomoko;Takashita,Emi;Fujisaki,Seiic
**EPI_ISL_405855**	EPI1669751|A/KANAGAWA/A	Asia / Japan	2019-12-	A/KANAGAWA/AC192	National Institute of Infectious Diseases (NIID)	National Institute of Infectious Diseases	Kuwahara,Tomoko;Takashita,Emi;Fujisaki,Seiic
**EPI_ISL_405869**	EPI1669779|A/Busan/1236/20	Asia / Korea, Republic of	2019-09-	A/Busan/1236/2019	National Institute of Health	National Institute of Infectious Diseases	Kuwahara,Tomoko;Takashita,Emi;Fujisaki,Seiic
**EPI_ISL_405870**	EPI1669781|A/Daegu/1266/20	Asia / Korea, Republic of	2019-10-	A/Daegu/1266/2019	National Institute of Health	National Institute of Infectious Diseases	Kuwahara,Tomoko;Takashita,Emi;Fujisaki,Seiic
**EPI_ISL_405871**	EPI1669783|A/Daejeon/1248/	Asia / Korea, Republic of	2019-10-	A/Daejeon/1248/2019	National Institute of Health	National Institute of Infectious Diseases	Kuwahara,Tomoko;Takashita,Emi;Fujisaki,Seiic
**EPI_ISL_405872**	EPI1669785|A/Gangwon/1256/	Asia / Korea, Republic of	2019-10-	A/Gangwon/1256/201	National Institute of Health	National Institute of Infectious Diseases	Kuwahara,Tomoko;Takashita,Emi;Fujisaki,Seiic
**EPI_ISL_405873**	EPI1669787|A/Jeonbuk/1259/	Asia / Korea, Republic of	2019-10-	A/Jeonbuk/1259/2019	National Institute of Health	National Institute of Infectious Diseases	Kuwahara,Tomoko;Takashita,Emi;Fujisaki,Seiic
**EPI_ISL_405877**	EPI1669795|A/NAGANO/2820	Asia / Japan	2019-12-	A/NAGANO/2820/201	Nagano Environmental Conservation Research Institute	National Institute of Infectious Diseases	Kuwahara,Tomoko;Takashita,Emi;Fujisaki,Seiic
**EPI_ISL_405880**	EPI1669801|A/WAKAYAMA/1	Asia / Japan	2019-12-	A/WAKAYAMA/111/20	Wakayama Prefectural Research Center of Environment and Public	National Institute of Infectious Diseases	Kuwahara,Tomoko;Takashita,Emi;Fujisaki,Seiic
**EPI_ISL_405881**	EPI1669803|A/Myanmar/1258/	Asia / Myanmar	2019-08-	A/Myanmar/1258/201	National Health Laboratory	National Institute of Infectious Diseases	Kuwahara,Tomoko;Takashita,Emi;Fujisaki,Seiic
**EPI_ISL_405924**	EPI1669929|N10025151.4	Oceania / Australia / Victoria /	2019-06-	A/Victoria/91/2019	Victorian Infectious Diseases Reference Laboratory	WHO Collaborating Centre for Reference	Deng,Y-M; Iannello,P; Lau,H; Todd,A;
**EPI_ISL_405925**	EPI1669932|N10025153.4	Oceania / Australia / Victoria /	2019-06-	A/Victoria/101/2019	Victorian Infectious Diseases Reference Laboratory	WHO Collaborating Centre for Reference	Deng,Y-M; Iannello,P; Lau,H; Todd,A;
**EPI_ISL_405929**	EPI1669944|N10025158.4	Oceania / Australia / Victoria /	2019-06-	A/Victoria/96/2019	Victorian Infectious Diseases Reference Laboratory	WHO Collaborating Centre for Reference	Deng,Y-M; Iannello,P; Lau,H; Todd,A;
**EPI_ISL_405935**	EPI1669962|N10025165.4	Oceania / Australia / Victoria /	2019-06-	A/Victoria/113/2019	Victorian Infectious Diseases Reference Laboratory	WHO Collaborating Centre for Reference	Deng,Y-M; Iannello,P; Lau,H; Todd,A;
**EPI_ISL_405936**	EPI1669965|N10025168.4	Oceania / Australia / Victoria /	2019-06-	A/Victoria/118/2019	Victorian Infectious Diseases Reference Laboratory	WHO Collaborating Centre for Reference	Deng,Y-M; Iannello,P; Lau,H; Todd,A;
**EPI_ISL_405937**	EPI1669968|N10025169.4	Oceania / Australia / Victoria /	2019-06-	A/Victoria/119/2019	Victorian Infectious Diseases Reference Laboratory	WHO Collaborating Centre for Reference	Deng,Y-M; Iannello,P; Lau,H; Todd,A;
**EPI_ISL_405939**	EPI1669974|N10025178.4	Oceania / Australia / Victoria	2019-07-	A/Victoria/145/2019	Victorian Infectious Diseases Reference Laboratory	WHO Collaborating Centre for Reference	Deng,Y-M; Iannello,P; Lau,H; Todd,A;
**EPI_ISL_405960**	EPI1670037|N10028125.4	Asia / Thailand / Chanthaburi	2019-07-	A/Chanthaburi/2377/2	WHO National Influenza Centre, National Institute of Medical Research	WHO Collaborating Centre for Reference	Deng,Y-M; Iannello,P; Lau,H; Todd,A;
**EPI_ISL_405965**	EPI1670052|N10028130.4	Asia / Sri Lanka / Colombo	2019-07-	A/Sri Lanka/26/2019	Medical Research Institute	WHO Collaborating Centre for Reference	Deng,Y-M; Iannello,P; Lau,H; Todd,A;
**EPI_ISL_405973**	EPI1670076|N10028140.4	Oceania / Australia / Victoria /	2019-08-	A/Victoria/2768/2019	Royal Chidrens Hospital	WHO Collaborating Centre for Reference	Deng,Y-M; Iannello,P; Lau,H; Todd,A;
**EPI_ISL_405993**	EPI1670135|N10025152.4	Oceania / Australia / Victoria /	2019-06-	A/Victoria/100/2019	Victorian Infectious Diseases Reference Laboratory	WHO Collaborating Centre for Reference	Deng,Y-M; Iannello,P; Lau,H; Todd,A;
**EPI_ISL_405998**	EPI1670150|N10025170.4	Oceania / Australia / Victoria /	2019-06-	A/Victoria/120/2019	Victorian Infectious Diseases Reference Laboratory	WHO Collaborating Centre for Reference	Deng,Y-M; Iannello,P; Lau,H; Todd,A;
**EPI_ISL_406015**	EPI1670201|N10028141.4	Oceania / Australia / New	2019-07-	A/Sydney/370/2019	Clinical Virology Unit, CDIM	WHO Collaborating Centre for Reference	Deng,Y-M; Iannello,P; Lau,H; Todd,A;
**EPI_ISL_406017**	EPI1670207|N10028143.4	Oceania / Australia / New	2019-08-	A/Sydney/398/2019	Clinical Virology Unit, CDIM	WHO Collaborating Centre for Reference	Deng,Y-M; Iannello,P; Lau,H; Todd,A;
**EPI_ISL_406169**	EPI1670514|34-	Africa / Senegal	2019-09-	A/Dakar/21/2019	Institut Pasteur de Dakar	Crick Worldwide Influenza Centre	NA
**EPI_ISL_406170**	EPI1670516|32-	Africa / Senegal	2019-08-	A/Dakar/22/2019	Institut Pasteur de Dakar	Crick Worldwide Influenza Centre	NA
**EPI_ISL_406196**	EPI1670568|15-A_Qatar_10-	Asia / Qatar	2019-11-	A/Qatar/10-VI-19-	Supreme Health Council	Crick Worldwide Influenza Centre	NA
**EPI_ISL_406200**	EPI1670576|18-A_Qatar_13-	Asia / Qatar	2019-10-	A/Qatar/13-VI-19-	Supreme Health Council	Crick Worldwide Influenza Centre	NA
**EPI_ISL_406204**	EPI1670584|16-A_Qatar_32-	Asia / Qatar	2019-11-	A/Qatar/32-VI-19-	Supreme Health Council	Crick Worldwide Influenza Centre	NA
**EPI_ISL_406207**	EPI1670590|26-A_Qatar_47-	Asia / Qatar	2019-10-	A/Qatar/47-VI-19-	Supreme Health Council	Crick Worldwide Influenza Centre	NA
**EPI_ISL_406209**	EPI1670594|24-A_Qatar_47-	Asia / Qatar	2019-10-	A/Qatar/47-VI-19-	Supreme Health Council	Crick Worldwide Influenza Centre	NA
**EPI_ISL_406211**	EPI1670598|20-A_Qatar_10-	Asia / Qatar	2019-10-	A/Qatar/47-VI-19-	Supreme Health Council	Crick Worldwide Influenza Centre	NA
**EPI_ISL_406272**	EPI1670717|16-	Europe / France	2019-09-	A/Cagnes sur	CNR Virus des Infections Respiratoires - France SUD	Crick Worldwide Influenza Centre	NA
**EPI_ISL_406296**	EPI1670765|35-	Asia / Hong Kong (SAR)	2019-11-	A/Hong	Government Virus Unit	Crick Worldwide Influenza Centre	NA
**EPI_ISL_406310**	EPI1670792|51-	Europe / Iceland	2019-10-	A/Iceland/81/2019	Landspitali - University Hospital	Crick Worldwide Influenza Centre	NA
**EPI_ISL_406553**	EPI1671186|LNS6218442_HA	Europe / Luxembourg	2019-12-	A/Luxembourg/LNS62	Laboratoire National de Santé	Laboratoire National de Santé	Wienecke-Baldacchino, Anke; Fournier,
**EPI_ISL_406602**	EPI1671450|S10028869.4	Oceania / Australia / New	2019-08-	A/Sydney/391/2019	Clinical Virology Unit, CDIM	WHO Collaborating Centre for Reference	Deng,Y-M; Iannello,P; Lau,H; Todd,A;
**EPI_ISL_406609**	EPI1671468|S10032412.4	Oceania / Australia / New	2019-08-	A/Sydney/538/2019	Prince of Wales Hospital	WHO Collaborating Centre for Reference	Deng,Y-M; Iannello,P; Lau,H; Todd,A;
**EPI_ISL_406617**	EPI1671484|S10032232.4	Oceania / Australia / New	2019-08-	A/Sydney/379/2019	Clinical Virology Unit, CDIM	WHO Collaborating Centre for Reference	Deng,Y-M; Iannello,P; Lau,H; Todd,A;
**EPI_ISL_406619**	EPI1671489|N10032010.4	Oceania / Australia / Western	2019-06-	A/Perth/117/2019	Pathwest QE II Medical Centre	WHO Collaborating Centre for Reference	Deng,Y-M; Iannello,P; Lau,H; Todd,A;
**EPI_ISL_406621**	EPI1671495|N10032065.4	Oceania / Australia / New	2019-07-	A/Sydney/330/2019	Clinical Virology Unit, CDIM	WHO Collaborating Centre for Reference	Deng,Y-M; Iannello,P; Lau,H; Todd,A;
**EPI_ISL_406624**	EPI1671504|N10032079.4	Oceania / New Zealand /	2019-08-	A/Wellington/52/2019	Institute of Environmental Science and Research (ESR)	WHO Collaborating Centre for Reference	Deng,Y-M; Iannello,P; Lau,H; Todd,A;
**EPI_ISL_406630**	EPI1671522|N10032246.4	Oceania / Australia /	2019-08-	A/Brisbane/103/2019	Queensland Health Forensic and Scientific Services	WHO Collaborating Centre for Reference	Deng,Y-M; Iannello,P; Lau,H; Todd,A;
**EPI_ISL_406671**	EPI1671645|N10034481.4	Oceania / Australia / New	2019-09-	A/Sydney/468/2019	Clinical Virology Unit, CDIM	WHO Collaborating Centre for Reference	Deng,Y-M; Iannello,P; Lau,H; Todd,A;
**EPI_ISL_406672**	EPI1671648|N10034482.4	Oceania / Australia / New	2019-09-	A/Sydney/471/2019	Clinical Virology Unit, CDIM	WHO Collaborating Centre for Reference	Deng,Y-M; Iannello,P; Lau,H; Todd,A;
**EPI_ISL_406682**	EPI1671678|N10034492.4	Oceania / Australia /	2019-09-	A/Brisbane/1129/2019	Institute of Medical and Veterinary Science (IMVS)	WHO Collaborating Centre for Reference	Deng,Y-M; Iannello,P; Lau,H; Todd,A;
**EPI_ISL_406691**	EPI1671705|N10034505.4	Oceania / Australia / Victoria /	2019-09-	A/Victoria/2509/2019	Alfred Hospital	WHO Collaborating Centre for Reference	Deng,Y-M; Iannello,P; Lau,H; Todd,A;
**EPI_ISL_406694**	EPI1671714|N10034508.4	Oceania / Australia / Victoria /	2019-09-	A/Victoria/2503/2019	Alfred Hospital	WHO Collaborating Centre for Reference	Deng,Y-M; Iannello,P; Lau,H; Todd,A;
**EPI_ISL_406825**	EPI1671929|N10034776.4	Asia / Philippines / Province of	2019-10-	A/Philippines/38/2019	Research Institute of Tropical Medicine	WHO Collaborating Centre for Reference	Deng,Y-M; Iannello,P; Lau,H; Todd,A;
**EPI_ISL_407460**	EPI1674518|66-	Europe / Belgium	2019-12-	A/Belgium/G0540/201	Scientific Institute of Public Health	Crick Worldwide Influenza Centre	NA
**EPI_ISL_407948**	EPI1676112|A/Denmark/217/2	Europe / Denmark	2020-01-	A/Denmark/217/2020	Statens Serum Institute	Statens Serum Institute	Trebbien, R.
**EPI_ISL_408022**	EPI1676485|A_ENG_1946007	Europe / United Kingdom /	2019-11-	A/England/194600762	Microbiology Services Colindale, Public Health England	Microbiology Services Colindale, Public	Thompson,C.
**EPI_ISL_408041**	EPI1676637|A_ENG_394_201	Europe / United Kingdom /	2019-11-	A/England/394/2019	Microbiology Services Colindale, Public Health England	Microbiology Services Colindale, Public	Thompson,C.
**EPI_ISL_408043**	EPI1676653|A_ENG_395_201	Europe / United Kingdom /	2019-11-	A/England/395/2019	Microbiology Services Colindale, Public Health England	Microbiology Services Colindale, Public	Thompson,C.
**EPI_ISL_408066**	EPI1676837|A_ENG_480_201	Europe / United Kingdom /	2019-11-	A/England/480/2019	Microbiology Services Colindale, Public Health England	Microbiology Services Colindale, Public	Thompson,C.
**EPI_ISL_408308**	EPI1678673|A_ENG_562_201	Europe / United Kingdom /	2019-11-	A/England/562/2019	Microbiology Services Colindale, Public Health England	Microbiology Services Colindale, Public	Thompson,C.
**EPI_ISL_408311**	EPI1678697|A_ENG_564_201	Europe / United Kingdom /	2019-11-	A/England/564/2019	Microbiology Services Colindale, Public Health England	Microbiology Services Colindale, Public	Thompson,C.
**EPI_ISL_408338**	EPI1678913|A_ENG_596_201	Europe / United Kingdom /	2019-12-	A/England/596/2019	Microbiology Services Colindale, Public Health England	Microbiology Services Colindale, Public	Thompson,C.
**EPI_ISL_408546**	EPI1679831|A/WAKAYAMA/9	Asia / Japan	2019-11-	A/WAKAYAMA/98/201	Wakayama Prefectural Research Center of Environment and Public	National Institute of Infectious Diseases	Kuwahara,Tomoko;Takashita,Emi;Fujisaki,Seiic
**EPI_ISL_409056**	EPI1683352|3001292797_N8	Asia / Thailand	2019-09-	A/Trang/407/2019	WHO National Influenza Centre, National Institute of Medical Research	Centers for Disease Control and Prevention	NA
**EPI_ISL_409059**	EPI1683374|3001292789_N8	Asia / Thailand	2019-08-	A/Yasothon/384/2019	WHO National Influenza Centre, National Institute of Medical Research	Centers for Disease Control and Prevention	NA
**EPI_ISL_409065**	EPI1683422|3000826390_N8	South America / Colombia	2019-12-	A/Colombia/7231/201	Instituto Nacional de Salud de Columbia	Centers for Disease Control and Prevention	NA

**EPI_ISL_409066**	EPI1683426|3000826391_N8	South America / Colombia	2019-10-	A/Colombia/7239/201	Instituto Nacional de Salud de Columbia	Centers for Disease Control and Prevention	NA
**EPI_ISL_409068**	EPI1683434|3000826400_N8	South America / Colombia	2019-12-	A/Colombia/7394/201	Instituto Nacional de Salud de Columbia	Centers for Disease Control and Prevention	NA
**EPI_ISL_409100**	EPI1686067|3000826276_N8	Africa / Burkina Faso	2019-08-	A/Burkina	IRSS	Centers for Disease Control and Prevention	NA
**EPI_ISL_409102**	EPI1683698|3026054634_ZZ	North America / United States /	2019-12-	A/California/242/2019	California Department of Health Services	Centers for Disease Control and Prevention	NA
**EPI_ISL_409341**	EPI1685511|3001292955_N8	North America / El Salvador	2019-10-	A/El	Contiguo a Hospital Rosales	Centers for Disease Control and Prevention	NA
**EPI_ISL_409343**	EPI1685527|3000826200_N8	Africa / Togo	2019-12-	A/Togo/1941/2019	Institute National D'Hygiene	Centers for Disease Control and Prevention	NA
**EPI_ISL_409344**	EPI1685535|3000826198_N8	Africa / Togo	2019-11-	A/Togo/1911/2019	Institute National D'Hygiene	Centers for Disease Control and Prevention	NA
**EPI_ISL_409358**	EPI1685642|3000826170_N8	Africa / Togo	2019-11-	A/Togo/1658/2019	Institute National D'Hygiene	Centers for Disease Control and Prevention	NA
**EPI_ISL_409363**	EPI1685678|3000826156_N8	Africa / Togo	2019-11-	A/Togo/1644/2019	Institute National D'Hygiene	Centers for Disease Control and Prevention	NA
**EPI_ISL_409379**	EPI1685797|3001292842_N8	South America / Paraguay	2019-11-	A/Paraguay/9880/201	Central Laboratory of Public Health	Centers for Disease Control and Prevention	NA
**EPI_ISL_409392**	EPI1685893|3001292878_N8	Asia / Hong Kong (SAR)	2019-12-	A/Hong	Government Virus Unit	Centers for Disease Control and Prevention	NA
**EPI_ISL_409411**	EPI1686037|3000826281_N8	Africa / Burkina Faso	2019-09-	A/Burkina	IRSS	Centers for Disease Control and Prevention	NA
**EPI_ISL_409413**	EPI1686053|3000826283_N8	Africa / Burkina Faso	2019-09-	A/Burkina	IRSS	Centers for Disease Control and Prevention	NA
**EPI_ISL_409415**	EPI1686075|3000826275_N8	Africa / Burkina Faso	2019-08-	A/Burkina	IRSS	Centers for Disease Control and Prevention	NA
**EPI_ISL_409422**	EPI1686121|3001292947_N8	South America / Peru	2019-12-	A/Peru/6219/2019	Laboratorio de Referencia Nacional Virus Respiratorios, Instituto	Centers for Disease Control and Prevention	NA
**EPI_ISL_409963**	EPI1689434|6-	Europe / Cyprus	2020-01-	A/Cyprus/11/2020	Nicosia General Hospital	Crick Worldwide Influenza Centre	NA
**EPI_ISL_409990**	EPI1689488|20-	Asia / Kyrgyzstan	2019-12-	A/Kyrgyzstan/205/201	National Virology Laboratory, Center Microbiological Investigations	Crick Worldwide Influenza Centre	NA
**EPI_ISL_409993**	EPI1689494|16-	Asia / Kyrgyzstan	2019-12-	A/Kyrgyzstan/308/201	National Virology Laboratory, Center Microbiological Investigations	Crick Worldwide Influenza Centre	NA
**EPI_ISL_410081**	EPI1690141|HA_20-	Europe / Germany	2020-01-	A/Berlin/9/2020	Robert Koch-Institute Nationales Referenzzentrum für Influenza	Robert Koch Institute Nationales	NA
**EPI_ISL_410134**	EPI1690238|A/Para/159307-	South America / Brazil / Estado	2019-10-	A/Para/159307-	Evandro Chagas Institute	Evandro Chagas Institute	NA
**EPI_ISL_410145**	EPI1690271|N10035805.4	Oceania / Australia / Victoria /	2019-12-	A/Victoria/2590/2019	Alfred Hospital	WHO Collaborating Centre for Reference	Deng,Y-M; Iannello,P; Lau,H; Todd,A;
**EPI_ISL_410147**	EPI1690277|N10035807.4	Oceania / Australia / Victoria /	2019-12-	A/Victoria/2595/2019	Alfred Hospital	WHO Collaborating Centre for Reference	Deng,Y-M; Iannello,P; Lau,H; Todd,A;
**EPI_ISL_410149**	EPI1690283|N10035809.4	Oceania / Australia / Australian	2019-12-	A/Canberra/20A/2019	Canberra Hospital	WHO Collaborating Centre for Reference	Deng,Y-M; Iannello,P; Lau,H; Todd,A;
**EPI_ISL_410150**	EPI1690286|N10035810.4	Oceania / Australia / Australian	2019-12-	A/Canberra/4A/2019	Canberra Hospital	WHO Collaborating Centre for Reference	Deng,Y-M; Iannello,P; Lau,H; Todd,A;
**EPI_ISL_410153**	EPI1690295|N10035813.4	Oceania / Australia / Tasmania	2019-11-	A/Tasmania/637/2019	Hobart Pathology	WHO Collaborating Centre for Reference	Deng,Y-M; Iannello,P; Lau,H; Todd,A;
**EPI_ISL_410154**	EPI1690298|N10035814.4	Oceania / Australia / Tasmania	2019-11-	A/Tasmania/650/2019	Hobart Pathology	WHO Collaborating Centre for Reference	Deng,Y-M; Iannello,P; Lau,H; Todd,A;
**EPI_ISL_410155**	EPI1690301|N10035815.4	Asia / Philippines /	2019-11-	A/Philippines/42/2019	Research Institute of Tropical Medicine	WHO Collaborating Centre for Reference	Deng,Y-M; Iannello,P; Lau,H; Todd,A;
**EPI_ISL_410157**	EPI1690307|N10035817.4	Oceania / Australia / Victoria /	2019-12-	A/Victoria/2867/2019	Royal Chidrens Hospital	WHO Collaborating Centre for Reference	Deng,Y-M; Iannello,P; Lau,H; Todd,A;
**EPI_ISL_410161**	EPI1690319|N10035821.4	Asia / Cambodia / Siem Reap	2019-12-	A/Cambodia/E010938	Institute Pasteur du Cambodia	WHO Collaborating Centre for Reference	Deng,Y-M; Iannello,P; Lau,H; Todd,A;
**EPI_ISL_410162**	EPI1690322|N10035822.4	Asia / Cambodia / Phnom Penh	2019-12-	A/Cambodia/E010937	Institute Pasteur du Cambodia	WHO Collaborating Centre for Reference	Deng,Y-M; Iannello,P; Lau,H; Todd,A;
**EPI_ISL_410164**	EPI1690328|N10035824.4	Asia / Cambodia / Phnom Penh	2019-11-	A/Cambodia/D121636	Institute Pasteur du Cambodia	WHO Collaborating Centre for Reference	Deng,Y-M; Iannello,P; Lau,H; Todd,A;
**EPI_ISL_410167**	EPI1690337|N10035827.4	Oceania / Australia / Victoria /	2019-12-	A/Victoria/2873/2019	Royal Chidrens Hospital	WHO Collaborating Centre for Reference	Deng,Y-M; Iannello,P; Lau,H; Todd,A;
**EPI_ISL_410591**	EPI1691936|2020-CX0201.4	Asia / China / Jiangsu	2019-12-	A/Jiangsu-	WHO Chinese National Influenza Center	WHO Chinese National Influenza Center	Xiyan,Li;Jia,Liu; Dayan,Wang
**EPI_ISL_410603**	EPI1691972|2020-CX0188.4	Asia / China / Guangxi	2019-12-	A/Guangxi-	WHO Chinese National Influenza Center	WHO Chinese National Influenza Center	Xiyan,Li;Jia,Liu; Dayan,Wang
**EPI_ISL_410606**	EPI1691981|2020-CX0185.4	Asia / China / Beijing	2020-01-	A/Beijing-	WHO Chinese National Influenza Center	WHO Chinese National Influenza Center	Xiyan,Li;Jia,Liu; Dayan,Wang
**EPI_ISL_410608**	EPI1691987|2020-CX0183.4	Asia / China / Hubei	2020-01-	A/Hubei-	WHO Chinese National Influenza Center	WHO Chinese National Influenza Center	Xiyan,Li;Jia,Liu; Dayan,Wang
**EPI_ISL_410609**	EPI1691990|2020-CX0182.4	Asia / China / Hubei	2020-01-	A/Hubei-	WHO Chinese National Influenza Center	WHO Chinese National Influenza Center	Xiyan,Li;Jia,Liu; Dayan,Wang
**EPI_ISL_410611**	EPI1691996|2020-CX0180.4	Asia / China / Qinghai	2020-01-	A/Qinghai-	WHO Chinese National Influenza Center	WHO Chinese National Influenza Center	Xiyan,Li;Jia,Liu; Dayan,Wang
**EPI_ISL_410616**	EPI1692011|2020-CX0175.4	Asia / China / Henan	2020-01-	A/Henan-	WHO Chinese National Influenza Center	WHO Chinese National Influenza Center	Xiyan,Li;Jia,Liu; Dayan,Wang
**EPI_ISL_410659**	EPI1692140|2020-CX0128.4	Asia / China / Jiangxi	2019-11-	A/Jiangxi-	WHO Chinese National Influenza Center	WHO Chinese National Influenza Center	Xiyan,Li;Jia,Liu; Dayan,Wang
**EPI_ISL_410662**	EPI1692149|2020-CX0125.4	Asia / China / Chongqing	2019-12-	A/Chongqing-	WHO Chinese National Influenza Center	WHO Chinese National Influenza Center	Xiyan,Li;Jia,Liu; Dayan,Wang
**EPI_ISL_410665**	EPI1692158|2020-CX0122.4	Asia / China / Henan	2019-11-	A/Henan-	WHO Chinese National Influenza Center	WHO Chinese National Influenza Center	Xiyan,Li;Jia,Liu; Dayan,Wang
**EPI_ISL_410670**	EPI1692173|2020-CX0117.4	Asia / China / Guizhou	2019-12-	A/Guizhou-	WHO Chinese National Influenza Center	WHO Chinese National Influenza Center	Xiyan,Li;Jia,Liu; Dayan,Wang
**EPI_ISL_410676**	EPI1692191|2020-CX0110.4	Asia / China	2019-11-	A/Guangxi-	WHO Chinese National Influenza Center	WHO Chinese National Influenza Center	Xiyan,Li;Jia,Liu; Dayan,Wang
**EPI_ISL_410677**	EPI1692194|2020-CX0109.4	Asia / China / Zhejiang	2019-12-	A/Zhejiang-	WHO Chinese National Influenza Center	WHO Chinese National Influenza Center	Xiyan,Li;Jia,Liu; Dayan,Wang
**EPI_ISL_410684**	EPI1692215|2020-CX0102.4	Asia / China	2019-11-	A/Tianjin-	WHO Chinese National Influenza Center	WHO Chinese National Influenza Center	Xiyan,Li;Jia,Liu; Dayan,Wang
**EPI_ISL_410688**	EPI1692227|2020-CX0098.4	Asia / China / Hunan	2019-11-	A/Hunan-	WHO Chinese National Influenza Center	WHO Chinese National Influenza Center	Xiyan,Li;Jia,Liu; Dayan,Wang
**EPI_ISL_410695**	EPI1692248|2020-CX0091.4	Asia / China / Shaanxi	2019-10-	A/Shaanxi-	WHO Chinese National Influenza Center	WHO Chinese National Influenza Center	Xiyan,Li;Jia,Liu; Dayan,Wang
**EPI_ISL_410697**	EPI1692254|2020-CX0089.4	Asia / China / Sichuan	2019-10-	A/Sichuan-	WHO Chinese National Influenza Center	WHO Chinese National Influenza Center	Xiyan,Li;Jia,Liu; Dayan,Wang
**EPI_ISL_410698**	EPI1692257|2020-CX0088.4	Asia / China / Chongqing	2019-09-	A/Chongqing-	WHO Chinese National Influenza Center	WHO Chinese National Influenza Center	Xiyan,Li;Jia,Liu; Dayan,Wang
**EPI_ISL_410700**	EPI1692263|2020-CX0086.4	Asia / China / Zhejiang	2019-10-	A/Zhejiang-	WHO Chinese National Influenza Center	WHO Chinese National Influenza Center	Xiyan,Li;Jia,Liu; Dayan,Wang
**EPI_ISL_410701**	EPI1692266|2020-CX0085.4	Asia / China / Jiangxi	2019-09-	A/Jiangxi-	WHO Chinese National Influenza Center	WHO Chinese National Influenza Center	Xiyan,Li;Jia,Liu; Dayan,Wang
**EPI_ISL_411131**	EPI1694558|65-A_Mauritius_I-	Africa / Mauritius	2020-01-	A/Mauritius/I-19/2020	Central Health Laboratory	Crick Worldwide Influenza Centre	NA
**EPI_ISL_411133**	EPI1694564|64-A_Mauritius_I-	Africa / Mauritius	2020-01-	A/Mauritius/I-34/2020	Central Health Laboratory	Crick Worldwide Influenza Centre	NA
**EPI_ISL_412064**	EPI1696281|A/Grenoble/1840/	Europe / France	2019-08-	A/Grenoble/1840/201	CNR Virus des Infections Respiratoires - France SUD	CNR Virus des Infections Respiratoires -	NA
**EPI_ISL_412107**	EPI1696367|A/Anantnag/AG_	Asia / India / envoi1	2019-11-	A/Anantnag/AG_159/2	Sher-i-Kashmir Institute of Medical Sciences	CNR Virus des Infections Respiratoires -	NA
**EPI_ISL_412123**	EPI1696437|3026055099_ZZ	North America / United States /	2019-12-	A/Idaho/51/2019	State of Idaho Bureau of Laboratories	Centers for Disease Control and Prevention	NA
**EPI_ISL_412129**	EPI1696485|3026055119_ZZ	North America / United States /	2020-01-	A/Rhode	Rhode Island Department of Health	Centers for Disease Control and Prevention	NA
**EPI_ISL_412137**	EPI1696549|3000826427_N8	Asia / Bahrain	2019-12-	A/Bahrain/808/2019	Ministry of Health Bahrain	Centers for Disease Control and Prevention	NA
**EPI_ISL_412153**	EPI1696676|3000826430_N8	Asia / Bahrain	2019-12-	A/Bahrain/835/2019	Ministry of Health Bahrain	Centers for Disease Control and Prevention	NA
**EPI_ISL_412154**	EPI1696684|3026055111_ZZ	North America / United States /	2019-11-	A/Idaho/50/2019	State of Idaho Bureau of Laboratories	Centers for Disease Control and Prevention	NA
**EPI_ISL_412171**	EPI1696819|3026055167_ZZ	North America / United States /	2020-01-	A/Florida/11/2020	Florida Department of Health-Tampa	Centers for Disease Control and Prevention	NA
**EPI_ISL_412543**	EPI1699409|A/South	Asia / Korea, Republic of	2019-12-	A/South	U.S. Air Force School of Aerospace Medicine	U.S. Air Force School of Aerospace	Gruner, W.E.; Fries, A.C.; Garrett, C.M.; Powell,
**EPI_ISL_413236**	EPI1702602|3001292935_N8	South America / Peru	2019-11-	A/Peru/113154619/20	Laboratorio de Referencia Nacional Virus Respiratorios, Instituto	Centers for Disease Control and Prevention	NA
**EPI_ISL_413249**	EPI1702706|3026055316_ZZ	North America / United States /	2020-02-	A/Wisconsin/39/2020	Wisconsin State Laboratory of Hygiene	Centers for Disease Control and Prevention	NA
**EPI_ISL_413715**	EPI1704843|3000826554_N8	Africa / Mali	2019-10-	A/Mali/169/2019	NIC Lab CVD-MALI	Centers for Disease Control and Prevention	NA
**EPI_ISL_413719**	EPI1704872|3000826560_N8	Africa / Mali	2019-11-	A/Mali/356/2019	NIC Lab CVD-MALI	Centers for Disease Control and Prevention	NA
**EPI_ISL_413722**	EPI1704896|3000826566_N8	Africa / Mali	2019-11-	A/Mali/365/2019	NIC Lab CVD-MALI	Centers for Disease Control and Prevention	NA

**EPI_ISL_413736**	EPI1704997|3000826542_N8	Africa / Mali	2019-10-	A/Mali/333/2019	NIC Lab CVD-MALI	Centers for Disease Control and Prevention	NA
**EPI_ISL_414057**	EPI1706223|A/England/10772/	Europe / United Kingdom	2019-12-	A/England/10772/201	U.S. Air Force School of Aerospace Medicine	U.S. Air Force School of Aerospace	Gruner, W.E.; Fries, A.C.; Garrett, C.M.; Powell,
**EPI_ISL_414382**	EPI1708172|A/WAKAYAMA/1	Asia / Japan	2019-11-	A/WAKAYAMA/100/20	Wakayama Prefectural Research Center of Environment and Public	National Institute of Infectious Diseases	Takashita,Emi;Fujisaki,Seiichiro;Shirakura,Masa
**EPI_ISL_414388**	EPI1708184|A/OSAKA/105/20	Asia / Japan	2019-12-	A/OSAKA/105/2019	Osaka Prefectural Institute of Public Health	National Institute of Infectious Diseases	Takashita,Emi;Fujisaki,Seiichiro;Shirakura,Masa
**EPI_ISL_414392**	EPI1708192|A/TOKYO/19438/	Asia / Japan	2019-12-	A/TOKYO/19438/2019	Tokyo Metropolitan Institute of Public Health	National Institute of Infectious Diseases	Takashita,Emi;Fujisaki,Seiichiro;Shirakura,Masa
**EPI_ISL_414602**	EPI1708332|HA-4312000540	Europe / Netherlands	2020-03-	A/Netherlands/10225/	National Institute for Public Health and the Environment (RIVM)	National Institute for Public Health and the	A,Meijer; P,Overduin; S, vd Brink
**EPI_ISL_414604**	EPI1708336|HA-4312000556	Europe / Netherlands	2020-03-	A/Netherlands/10234/	National Institute for Public Health and the Environment (RIVM)	National Institute for Public Health and the	A,Meijer; P,Overduin; S, vd Brink
**EPI_ISL_414607**	EPI1708342|HA-4312000576	Europe / Netherlands	2020-03-	A/Netherlands/10243/	National Institute for Public Health and the Environment (RIVM)	National Institute for Public Health and the	A,Meijer; P,Overduin; S, vd Brink
**EPI_ISL_414609**	EPI1708346|HA-4312000601	Europe / Netherlands	2020-03-	A/Netherlands/10248/	National Institute for Public Health and the Environment (RIVM)	National Institute for Public Health and the	A,Meijer; P,Overduin; S, vd Brink
**EPI_ISL_414845**	EPI1709675|A/South	Asia / Korea, Republic of	2020-01-	A/South	U.S. Air Force School of Aerospace Medicine	U.S. Air Force School of Aerospace	Gruner, W.E.; Fries, A.C.; Garrett, C.M.; Powell,
**EPI_ISL_414877**	EPI1709913|A/South	Asia / Korea, Republic of	2020-01-	A/South	U.S. Air Force School of Aerospace Medicine	U.S. Air Force School of Aerospace	Gruner, W.E.; Fries, A.C.; Garrett, C.M.; Powell,
**EPI_ISL_414881**	EPI1709941|A/South	Asia / Korea, Republic of	2020-01-	A/South	U.S. Air Force School of Aerospace Medicine	U.S. Air Force School of Aerospace	Gruner, W.E.; Fries, A.C.; Garrett, C.M.; Powell,
**EPI_ISL_414882**	EPI1709949|A/South	Asia / Korea, Republic of	2020-01-	A/South	U.S. Air Force School of Aerospace Medicine	U.S. Air Force School of Aerospace	Gruner, W.E.; Fries, A.C.; Garrett, C.M.; Powell,
**EPI_ISL_414888**	EPI1709997|A/South	Asia / Korea, Republic of	2020-01-	A/South	U.S. Air Force School of Aerospace Medicine	U.S. Air Force School of Aerospace	Gruner, W.E.; Fries, A.C.; Garrett, C.M.; Powell,
**EPI_ISL_414889**	EPI1710005|A/South	Asia / Korea, Republic of	2020-01-	A/South	U.S. Air Force School of Aerospace Medicine	U.S. Air Force School of Aerospace	Gruner, W.E.; Fries, A.C.; Garrett, C.M.; Powell,
**EPI_ISL_414890**	EPI1710013|A/South	Asia / Korea, Republic of	2020-01-	A/South	U.S. Air Force School of Aerospace Medicine	U.S. Air Force School of Aerospace	Gruner, W.E.; Fries, A.C.; Garrett, C.M.; Powell,
**EPI_ISL_415174**	EPI1711306|A/Nepal/19FL332	Asia / Nepal	2019-10-	A/Nepal/19FL3323/20	National Public Health Laboratory	National Institute of Infectious Diseases	Takashita,Emi;Fujisaki,Seiichiro;Shirakura,Masa
**EPI_ISL_415176**	EPI1711310|A/OSAKA/119/20	Asia / Japan	2019-12-	A/OSAKA/119/2019	Osaka Prefectural Institute of Public Health	National Institute of Infectious Diseases	Takashita,Emi;Fujisaki,Seiichiro;Shirakura,Masa
**EPI_ISL_415183**	EPI1711324|A/NAGANO-	Asia / Japan	2020-01-	A/NAGANO-C/6/2020	Nagano City Health Center	National Institute of Infectious Diseases	Takashita,Emi;Fujisaki,Seiichiro;Shirakura,Masa
**EPI_ISL_415759**	EPI1713958|3026055581_ZZ	North America / United States /	2020-02-	A/New York/25/2020	New York State Department of Health	Centers for Disease Control and Prevention	NA
**EPI_ISL_415768**	EPI1714029|3026055610_ZZ	North America / United States /	2020-02-	A/California/33/2020	California Department of Health Services	Centers for Disease Control and Prevention	NA
**EPI_ISL_416148**	EPI1716182|63-	Africa / Morocco	2020-01-	A/Beni	Institut National d'Hygiène	Crick Worldwide Influenza Centre	NA
**EPI_ISL_416168**	EPI1716220|12-	Africa / Cameroon	2019-11-	A/Cameroon/8409/20	Centre Pasteur du Cameroun	Crick Worldwide Influenza Centre	NA
**EPI_ISL_416180**	EPI1716244|82-	Africa / Cameroon	2019-12-	A/Cameroon/9274/20	Centre Pasteur du Cameroun	Crick Worldwide Influenza Centre	NA
**EPI_ISL_416181**	EPI1716246|83-	Africa / Cameroon	2019-12-	A/Cameroon/9285/20	Centre Pasteur du Cameroun	Crick Worldwide Influenza Centre	NA
**EPI_ISL_416182**	EPI1716248|18-	Europe / Spain	2019-08-	A/Catalonia/11896S/2	Barcelona, Facultad de Medicina	Crick Worldwide Influenza Centre	NA
**EPI_ISL_416217**	EPI1716316|58-	Africa / Morocco	2020-02-	A/Fes/1085/2020	Institut National d'Hygiène	Crick Worldwide Influenza Centre	NA
**EPI_ISL_416218**	EPI1716318|57-	Africa / Morocco	2020-02-	A/Fes/1091/2020	Institut National d'Hygiène	Crick Worldwide Influenza Centre	NA
**EPI_ISL_416219**	EPI1716320|56-	Africa / Morocco	2020-02-	A/Fes/1092/2020	Institut National d'Hygiène	Crick Worldwide Influenza Centre	NA
**EPI_ISL_416262**	EPI1716406|60-	Africa / Morocco	2020-02-	A/Marrakech/1090/20	Institut National d'Hygiène	Crick Worldwide Influenza Centre	NA
**EPI_ISL_416269**	EPI1716419|62-	Africa / Morocco	2020-02-	A/Meknes/1061/2020	Institut National d'Hygiène	Crick Worldwide Influenza Centre	NA
**EPI_ISL_416271**	EPI1716423|70-	Africa / Morocco	2020-01-	A/Meknes/955/2020	Institut National d'Hygiène	Crick Worldwide Influenza Centre	NA
**EPI_ISL_416291**	EPI1716463|72-	Africa / Morocco	2020-01-	A/Rabat/787/2020	Institut National d'Hygiène	Crick Worldwide Influenza Centre	NA
**EPI_ISL_416292**	EPI1716465|69-	Africa / Morocco	2020-01-	A/Rabat/954/2020	Institut National d'Hygiène	Crick Worldwide Influenza Centre	NA
**EPI_ISL_416548**	EPI1716589|A/Netherlands/00	Europe / Netherlands / Arnhem	2020-03-	A/Netherlands/00462/	Erasmus Medical Center	Erasmus Medical Center	NA
**EPI_ISL_416551**	EPI1716598|A/Netherlands/00	Europe / Netherlands /	2020-03-	A/Netherlands/00466/	Erasmus Medical Center	Erasmus Medical Center	NA
**EPI_ISL_416552**	EPI1716601|A/Netherlands/00	Europe / Netherlands /	2020-03-	A/Netherlands/00467/	Erasmus Medical Center	Erasmus Medical Center	NA
**EPI_ISL_416554**	EPI1716607|A/Netherlands/00	Europe / Netherlands /	2020-03-	A/Netherlands/00474/	Erasmus Medical Center	Erasmus Medical Center	NA
**EPI_ISL_416556**	EPI1716613|A/Netherlands/00	Europe / Netherlands /	2020-03-	A/Netherlands/00477/	Erasmus Medical Center	Erasmus Medical Center	NA
**EPI_ISL_416557**	EPI1716615|A/Netherlands/00	Europe / Netherlands /	2020-03-	A/Netherlands/00426/	Erasmus Medical Center	Erasmus Medical Center	NA
**EPI_ISL_416560**	EPI1716617|A/Netherlands/00	Europe / Netherlands / Arnhem	2020-03-	A/Netherlands/00461/	Erasmus Medical Center	Erasmus Medical Center	NA
**EPI_ISL_416563**	EPI1716621|A/Netherlands/00	Europe / Netherlands /	2020-03-	A/Netherlands/00473/	Erasmus Medical Center	Erasmus Medical Center	NA
**EPI_ISL_416761**	EPI1716653|3026055638_ZZ	North America / United States /	2020-01-	A/Michigan/18/2020	Michigan Department of Community Health	Centers for Disease Control and Prevention	NA
**EPI_ISL_416763**	EPI1716669|3026055641_ZZ	North America / United States /	2020-02-	A/Wisconsin/40/2020	Wisconsin State Laboratory of Hygiene	Centers for Disease Control and Prevention	NA
**EPI_ISL_416793**	EPI1716907|3000827204_N8	Asia / Kuwait	2019-11-	A/Kuwait/7340/2019	Public Health Laboratory, Virology Unit	Centers for Disease Control and Prevention	NA
**EPI_ISL_416794**	EPI1716915|3001292815_N8	Asia / Thailand	2019-11-	A/Ayutthaya/455/2019	WHO National Influenza Centre, National Institute of Medical Research	Centers for Disease Control and Prevention	NA
**EPI_ISL_416815**	EPI1717082|3000827348_N8	South America / Brazil	2019-10-	A/Rio Grande Do	Instituto Oswaldo Cruz FIOCRUZ - Laboratory of Respiratory Viruses	Centers for Disease Control and Prevention	NA
**EPI_ISL_417053**	EPI1718572|A/Taiwan/83294/	Asia / Taiwan	2019-09-	A/Taiwan/83294/2019	Center for Disease Control	National Institute of Infectious Diseases	Takashita,Emi;Fujisaki,Seiichiro;Shirakura,Masa
**EPI_ISL_417054**	EPI1718575|A/Taiwan/83330/	Asia / Taiwan	2019-10-	A/Taiwan/83330/2019	Center for Disease Control	National Institute of Infectious Diseases	Takashita,Emi;Fujisaki,Seiichiro;Shirakura,Masa
**EPI_ISL_417061**	EPI1718596|A/YOKOHAMA/6	Asia / Japan	2020-02-	A/YOKOHAMA/68/202	Yokohama City Institute of Public Health.	National Institute of Infectious Diseases	Takashita,Emi;Fujisaki,Seiichiro;Shirakura,Masa
**EPI_ISL_418521**	EPI1719204|3000827188_N8	Asia / Kuwait	2019-09-	A/Kuwait/4688/2019	Public Health Laboratory, Virology Unit	Centers for Disease Control and Prevention	NA
**EPI_ISL_418533**	EPI1719300|3026055765_ZZ	North America / United States /	2020-02-	A/Arizona/11/2020	Arizona Department of Health Services	Centers for Disease Control and Prevention	NA
**EPI_ISL_418534**	EPI1719308|3026055766_ZZ	North America / United States /	2020-03-	A/Arizona/13/2020	Arizona Department of Health Services	Centers for Disease Control and Prevention	NA
**EPI_ISL_419087**	EPI1720617|A/Saint-	Europe / Russian Federation /	2020-03-	A/Saint-Petersburg/RII-	Research Institute of Influenza, Ministry of Healthcare of the Russian	WHO National Influenza Centre Russian	NA
**EPI_ISL_419208**	EPI1721385|A/Khabarovsk/17/	Europe / Russian Federation /	2020-01-	A/Khabarovsk/17/2020	Center of Hygiene and Epidemiology in Khabarovsk Territory	WHO National Influenza Centre Russian	NA
**EPI_ISL_419267**	EPI1721436|N10036373.4	Asia / Sri Lanka / Western	2019-12-	A/Sri Lanka/55/2019	Medical Research Institute	WHO Collaborating Centre for Reference	Deng,Y-M; Iannello,P; Lau,H; Todd,A;
**EPI_ISL_419269**	EPI1721452|N10036375.4	Asia / Sri Lanka / Western	2020-01-	A/Sri Lanka/38/2020	Medical Research Institute	WHO Collaborating Centre for Reference	Deng,Y-M; Iannello,P; Lau,H; Todd,A;
**EPI_ISL_419270**	EPI1721460|N10036376.4	Oceania / Australia / South	2020-01-	A/South	Institute of Medical and Veterinary Science (IMVS)	WHO Collaborating Centre for Reference	Deng,Y-M; Iannello,P; Lau,H; Todd,A;
**EPI_ISL_419273**	EPI1721484|N10036379.4	Oceania / Australia / South	2020-01-	A/South	Institute of Medical and Veterinary Science (IMVS)	WHO Collaborating Centre for Reference	Deng,Y-M; Iannello,P; Lau,H; Todd,A;
**EPI_ISL_419280**	EPI1721540|N10036386.4	Asia / Thailand /	2019-10-	A/Nakhonphanom/485	WHO National Influenza Centre, National Institute of Medical Research	WHO Collaborating Centre for Reference	Deng,Y-M; Iannello,P; Lau,H; Todd,A;
**EPI_ISL_419609**	EPI1722108|N10037158.4	Oceania / Australia / Australian	2020-01-	A/Canberra/59/2020	Canberra Hospital	WHO Collaborating Centre for Reference	Deng,Y-M; Iannello,P; Lau,H; Todd,A;
**EPI_ISL_419610**	EPI1722111|N10037159.4	Oceania / Australia / Australian	2020-01-	A/Canberra/58/2020	Canberra Hospital	WHO Collaborating Centre for Reference	Deng,Y-M; Iannello,P; Lau,H; Todd,A;
**EPI_ISL_419611**	EPI1722114|N10037160.4	Oceania / Australia / Australian	2020-01-	A/Canberra/49/2020	Canberra Hospital	WHO Collaborating Centre for Reference	Deng,Y-M; Iannello,P; Lau,H; Todd,A;
**EPI_ISL_419612**	EPI1722117|N10037162.4	Oceania / Australia / Northern	2020-03-	A/Darwin/85/2020	Influenza Surveillance Centre for Disease Control	WHO Collaborating Centre for Reference	Deng,Y-M; Iannello,P; Lau,H; Todd,A;
**EPI_ISL_419613**	EPI1722120|N10037163.4	Oceania / Australia / Victoria /	2020-01-	A/Victoria/62/2020	Alfred Hospital	WHO Collaborating Centre for Reference	Deng,Y-M; Iannello,P; Lau,H; Todd,A;
**EPI_ISL_419614**	EPI1722123|N10037164.4	Oceania / Australia / New	2020-02-	A/Sydney/1005/2020	Institute of Medical and Veterinary Science (IMVS)	WHO Collaborating Centre for Reference	Deng,Y-M; Iannello,P; Lau,H; Todd,A;
**EPI_ISL_419616**	EPI1722129|N10037166.4	Oceania / Australia / Victoria /	2020-02-	A/Victoria/31/2020	Victorian Infectious Diseases Reference Laboratory	WHO Collaborating Centre for Reference	Deng,Y-M; Iannello,P; Lau,H; Todd,A;
**EPI_ISL_419617**	EPI1722132|N10037167.4	Oceania / Australia / Australian	2020-01-	A/Canberra/62/2020	Canberra Hospital	WHO Collaborating Centre for Reference	Deng,Y-M; Iannello,P; Lau,H; Todd,A;

**EPI_ISL_419619**	EPI1722138|N10037169.4	Oceania / Australia / Australian	2020-01-	A/Canberra/68/2020	Canberra Hospital	WHO Collaborating Centre for Reference	Deng,Y-M; Iannello,P; Lau,H; Todd,A;
**EPI_ISL_419620**	EPI1722141|N10037171.4	Asia / Malaysia	2019-12-	A/Malaysia/RP4587/20	Institut Penyelidikan Perubatan	WHO Collaborating Centre for Reference	Deng,Y-M; Iannello,P; Lau,H; Todd,A;
**EPI_ISL_421098**	EPI1723455|3026055841_ZZ	North America / United States /	2020-03-	A/Washington/24/202	Washington State Public Health Laboratory	Centers for Disease Control and Prevention	NA
**EPI_ISL_421100**	EPI1723471|3026055812_ZZ	North America / United States /	2020-03-	A/Texas/42/2020	Houston Department of Health and Human Services	Centers for Disease Control and Prevention	NA
**EPI_ISL_421123**	EPI1723656|3000827470_N8	South America / Bolivia,	2019-10-	A/Bolivia/1978/2019	Instituto Nacional de Laboratoriosde Salud (INLASA)	Centers for Disease Control and Prevention	NA
**EPI_ISL_421642**	EPI1724517|HA-4312000648	Europe / Netherlands	2020-03-	A/Netherlands/10321/	National Institute for Public Health and the Environment (RIVM)	National Institute for Public Health and the	A,Meijer; P,Overduin; S, vd Brink
**EPI_ISL_421643**	EPI1724519|HA-4312000665	Europe / Netherlands	2020-03-	A/Netherlands/10325/	National Institute for Public Health and the Environment (RIVM)	National Institute for Public Health and the	A,Meijer; P,Overduin; S, vd Brink
**EPI_ISL_421646**	EPI1724525|HA-4312000724	Europe / Netherlands	2020-03-	A/Netherlands/10339/	National Institute for Public Health and the Environment (RIVM)	National Institute for Public Health and the	A,Meijer; P,Overduin; S, vd Brink
**EPI_ISL_421647**	EPI1724527|HA-4312000729	Europe / Netherlands	2020-03-	A/Netherlands/10341/	National Institute for Public Health and the Environment (RIVM)	National Institute for Public Health and the	A,Meijer; P,Overduin; S, vd Brink
**EPI_ISL_421650**	EPI1724533|HA-4312000814	Europe / Netherlands	2020-03-	A/Netherlands/10346/	National Institute for Public Health and the Environment (RIVM)	National Institute for Public Health and the	A,Meijer; P,Overduin; S, vd Brink
**EPI_ISL_424733**	EPI1725715|3026055870_ZZ	North America / United States /	2020-02-	A/Louisiana/15/2020	Louisiana Department of Health and Hospitals	Centers for Disease Control and Prevention	NA
**EPI_ISL_424734**	EPI1725723|3026055860_ZZ	North America / United States /	2020-03-	A/South	South Dakota Public Health Lab	Centers for Disease Control and Prevention	NA
**EPI_ISL_424736**	EPI1725739|3026055833_ZZ	North America / United States /	2020-03-	A/Connecticut/16/202	Connecticut Department. of Public Health	Centers for Disease Control and Prevention	NA
**EPI_ISL_424737**	EPI1725747|3026055834_ZZ	North America / United States /	2020-03-	A/Connecticut/17/202	Connecticut Department. of Public Health	Centers for Disease Control and Prevention	NA
**EPI_ISL_424738**	EPI1725755|3026055851_ZZ	North America / United States /	2020-03-	A/Maryland/19/2020	Maryland Department of Health and Mental Hygiene	Centers for Disease Control and Prevention	NA
**EPI_ISL_424739**	EPI1725763|3026055852_ZZ	North America / United States /	2020-03-	A/Maryland/20/2020	Maryland Department of Health and Mental Hygiene	Centers for Disease Control and Prevention	NA
**EPI_ISL_424745**	EPI1725811|3026055941_ZZ	North America / United States /	2020-03-	A/Wisconsin/52/2020	Wisconsin State Laboratory of Hygiene	Centers for Disease Control and Prevention	NA
**EPI_ISL_424750**	EPI1725851|3026055940_ZZ	North America / United States /	2020-03-	A/California/49/2020	California Department of Health Services	Centers for Disease Control and Prevention	NA
**EPI_ISL_424752**	EPI1725867|3026055942_ZZ	North America / United States /	2020-03-	A/Wisconsin/53/2020	Wisconsin State Laboratory of Hygiene	Centers for Disease Control and Prevention	NA
**EPI_ISL_424754**	EPI1725883|3026055886_ZZ	North America / United States /	2020-03-	A/Illinois/15/2020	Illinois Department of Public Health-Carbondale	Centers for Disease Control and Prevention	NA
**EPI_ISL_424755**	EPI1725891|3026055885_ZZ	North America / United States /	2020-03-	A/Illinois/14/2020	Illinois Department of Public Health-Carbondale	Centers for Disease Control and Prevention	NA
**EPI_ISL_427993**	EPI1730176|A/South	Asia / Korea, Republic of	2020-01-	A/South	U.S. Air Force School of Aerospace Medicine	U.S. Air Force School of Aerospace	Gruner, W.E.; Fries, A.C.; Garrett, C.M.; Powell,
**EPI_ISL_428426**	EPI1731952|A/MIYAZAKI/84/2	Asia / Japan	2019-12-	A/MIYAZAKI/84/2019	Miyazaki Prefectural Institute for Public Health and Environment	National Institute of Infectious Diseases	Takashita,Emi;Fujisaki,Seiichiro;Shirakura,Masa
**EPI_ISL_428429**	EPI1731971|A/NAGANO/2598	Asia / Japan	2019-09-	A/NAGANO/2598/201	Nagano Environmental Conservation Research Institute	National Institute of Infectious Diseases	Takashita,Emi;Fujisaki,Seiichiro;Shirakura,Masa
**EPI_ISL_428434**	EPI1731986|A/ISHIKAWA/149/	Asia / Japan	2019-12-	A/ISHIKAWA/149/201	Ishikawa Prefectural Institute of Public Health and Environmental	National Institute of Infectious Diseases	Takashita,Emi;Fujisaki,Seiichiro;Shirakura,Masa
**EPI_ISL_428529**	EPI1732147|A_ENG_575_201	Europe / United Kingdom /	2019-12-	A/England/575/2019	Microbiology Services Colindale, Public Health England	Microbiology Services Colindale, Public	NA
**EPI_ISL_428532**	EPI1732171|A_ENG_580_201	Europe / United Kingdom /	2019-12-	A/England/580/2019	Microbiology Services Colindale, Public Health England	Microbiology Services Colindale, Public	NA
**EPI_ISL_428539**	EPI1732227|A_ENG_616_201	Europe / United Kingdom /	2019-12-	A/England/616/2019	Microbiology Services Colindale, Public Health England	Microbiology Services Colindale, Public	NA
**EPI_ISL_428657**	EPI1733171|A_ENG_1951608	Europe / United Kingdom /	2019-12-	A/England/195160830	Microbiology Services Colindale, Public Health England	Microbiology Services Colindale, Public	NA
**EPI_ISL_429108**	EPI1733834|S10037671.4	Oceania / Australia / Northern	2020-03-	A/Darwin/132/2020	Influenza Surveillance Centre for Disease Control	WHO Collaborating Centre for Reference	Deng,Y-M; Iannello,P; Lau,H; Todd,A;
**EPI_ISL_429109**	EPI1733837|S10037672.4	Oceania / Australia / Victoria /	2020-03-	A/Victoria/79/2020	Royal Chidrens Hospital	WHO Collaborating Centre for Reference	Deng,Y-M; Iannello,P; Lau,H; Todd,A;
**EPI_ISL_429110**	EPI1733840|S10037673.4	Oceania / Australia / Northern	2020-03-	A/Darwin/106/2020	Influenza Surveillance Centre for Disease Control	WHO Collaborating Centre for Reference	Deng,Y-M; Iannello,P; Lau,H; Todd,A;
**EPI_ISL_429111**	EPI1733843|S10037674.4	Oceania / Australia / Northern	2020-03-	A/Darwin/113/2020	Influenza Surveillance Centre for Disease Control	WHO Collaborating Centre for Reference	Deng,Y-M; Iannello,P; Lau,H; Todd,A;
**EPI_ISL_429112**	EPI1733846|S10037675.4	Oceania / Australia / Northern	2020-03-	A/Darwin/94/2020	Influenza Surveillance Centre for Disease Control	WHO Collaborating Centre for Reference	Deng,Y-M; Iannello,P; Lau,H; Todd,A;
**EPI_ISL_429113**	EPI1733849|S10037676.4	Oceania / Australia / New	2020-02-	A/Sydney/2/2020	Childrens Hospital Westmead	WHO Collaborating Centre for Reference	Deng,Y-M; Iannello,P; Lau,H; Todd,A;
**EPI_ISL_429114**	EPI1733852|S10037677.4	Oceania / Australia / Western	2020-01-	A/Perth/20/2020	Pathwest QE II Medical Centre	WHO Collaborating Centre for Reference	Deng,Y-M; Iannello,P; Lau,H; Todd,A;
**EPI_ISL_429198**	EPI1737592|A/Khabarovsk/48	Europe / Russian Federation /	2020-03-	A/Khabarovsk/48V/20	Center of Hygiene and Epidemiology in Khabarovsk Territory	State Research Center of Virology and	Alexey,Danilenko; Ivan,Susloparov;
**EPI_ISL_430754**	EPI1734713|3026055984_ZZ	North America / United States /	2020-03-	A/California/52/2020	California Department of Health Services	Centers for Disease Control and Prevention	NA
**EPI_ISL_430756**	EPI1734729|3026055983_ZZ	North America / United States /	2020-03-	A/Utah/17/2020	Utah Department of Health	Centers for Disease Control and Prevention	NA
**EPI_ISL_431099**	EPI1735138|S10037460.4	Oceania / Australia / Australian	2019-12-	A/Canberra/18A/2019	Canberra Hospital	WHO Collaborating Centre for Reference	Deng,Y-M; Iannello,P; Lau,H; Todd,A;
**EPI_ISL_434387**	EPI1735272|73-	Africa / Algeria	2020-02-	A/Algiers/368/2020	Institut Pasteur d'Algerie	Crick Worldwide Influenza Centre	NA
**EPI_ISL_434393**	EPI1735284|67-	Europe / Germany	2020-03-	A/Bremen/7/2020	Robert Koch Institute Nationales Referenzzentrum für Influenza	Crick Worldwide Influenza Centre	NA
**EPI_ISL_434398**	EPI1735294|40-	Europe / Bulgaria	2020-03-	A/Bulgaria/1772/2020	National Centre of Infectious and	Crick Worldwide Influenza Centre	NA
**EPI_ISL_434418**	EPI1735333|88-	Europe / Cyprus	2020-02-	A/Cyprus/F804/2020	Nicosia General Hospital	Crick Worldwide Influenza Centre	NA
**EPI_ISL_434420**	EPI1735337|85-	Europe / Cyprus	2020-02-	A/Cyprus/F842/2020	Nicosia General Hospital	Crick Worldwide Influenza Centre	NA
**EPI_ISL_434426**	EPI1735349|66-A_Rheinland-	Europe / Germany	2020-03-	A/Rheinland-	Robert Koch Institute Nationales Referenzzentrum für Influenza	Crick Worldwide Influenza Centre	NA
**EPI_ISL_434433**	EPI1735363|62-	Europe / Slovenia	2020-02-	A/Slovenia/1270/2020	Laboratory for Virology, National Institute of Public Health	Crick Worldwide Influenza Centre	NA
**EPI_ISL_434438**	EPI1735373|58-	Europe / Slovenia	2020-03-	A/Slovenia/1708/2020	Laboratory for Virology, National Institute of Public Health	Crick Worldwide Influenza Centre	NA
**EPI_ISL_434439**	EPI1735375|56-	Europe / Slovenia	2020-03-	A/Slovenia/1752/2020	Laboratory for Virology, National Institute of Public Health	Crick Worldwide Influenza Centre	NA
**EPI_ISL_435197**	EPI1735631|A/Italy/11874/202	Europe / Italy	2020-02-	A/Italy/11874/2020	U.S. Air Force School of Aerospace Medicine	U.S. Air Force School of Aerospace	Gruner, W.E.; Fries, A.C.; Garrett, C.M.; Powell,
**EPI_ISL_435325**	EPI1736308|3026055953_ZZ	North America / United States /	2020-03-	A/Connecticut/18/202	Connecticut Department. of Public Health	Centers for Disease Control and Prevention	NA
**EPI_ISL_435326**	EPI1736316|3026055954_ZZ	North America / United States /	2020-03-	A/Connecticut/21/202	Connecticut Department. of Public Health	Centers for Disease Control and Prevention	NA
**EPI_ISL_443119**	EPI1737771|3026055995_ZZ	North America / United States /	2020-03-	A/Minnesota/21/2020	Minnesota Department of Health	Centers for Disease Control and Prevention	NA
**EPI_ISL_443120**	EPI1737779|3000827503_N8	Africa / Nigeria	2019-07-	A/Nigeria/3984/2019	NCDC Public Health Reference Laboratory	Centers for Disease Control and Prevention	NA
**EPI_ISL_443125**	EPI1737818|3000827512_N8	Africa / Nigeria	2019-08-	A/Nigeria/4115/2019	NCDC Public Health Reference Laboratory	Centers for Disease Control and Prevention	NA
**EPI_ISL_443126**	EPI1737826|3000827513_N8	Africa / Nigeria	2019-08-	A/Nigeria/4117/2019	NCDC Public Health Reference Laboratory	Centers for Disease Control and Prevention	NA
**EPI_ISL_443131**	EPI1737865|3000827493_N8	Africa / Nigeria	2019-07-	A/Nigeria/3956/2019	NCDC Public Health Reference Laboratory	Centers for Disease Control and Prevention	NA
**EPI_ISL_443133**	EPI1737881|3000827491_N8	Africa / Nigeria	2019-07-	A/Nigeria/3954/2019	NCDC Public Health Reference Laboratory	Centers for Disease Control and Prevention	NA
**EPI_ISL_443134**	EPI1737889|3000827490_N8	Africa / Nigeria	2019-06-	A/Nigeria/3952/2019	NCDC Public Health Reference Laboratory	Centers for Disease Control and Prevention	NA
**EPI_ISL_443137**	EPI1737913|3000827502_N8	Africa / Nigeria	2019-07-	A/Nigeria/3983/2019	NCDC Public Health Reference Laboratory	Centers for Disease Control and Prevention	NA
**EPI_ISL_443138**	EPI1737921|3000827497_N8	Africa / Nigeria	2019-06-	A/Nigeria/3975/2019	NCDC Public Health Reference Laboratory	Centers for Disease Control and Prevention	NA
**EPI_ISL_443139**	EPI1737929|3000827499_N8	Africa / Nigeria	2020	A/Nigeria/3979/2020	NCDC Public Health Reference Laboratory	Centers for Disease Control and Prevention	NA
**EPI_ISL_445203**	EPI1738545|A/Romania/1230/	Europe / Romania / Bucharest	2020-03-	A/Romania/1230/2020	National Institute for Infectious Diseases “Prof. Dr. Matei Balş”	National Institute of Infectious Diseases-Prof.	'Banica, Leontina; Surleac, Marius; Paraschiv,
**EPI_ISL_445204**	EPI1738552|A/Romania/1234/	Europe / Romania / Bucharest	2020-03-	A/Romania/1234/2020	National Institute for Infectious Diseases “Prof. Dr. Matei Balş”	National Institute of Infectious Diseases-Prof.	'Banica, Leontina; Surleac, Marius; Paraschiv,
**EPI_ISL_445205**	EPI1738615|A/Romania/1261/	Europe / Romania / Bucharest	2020-03-	A/Romania/1261/2020	National Institute for Infectious Diseases “Prof. Dr. Matei Balş”	National Institute of Infectious Diseases-Prof.	'Banica, Leontina; Surleac, Marius; Paraschiv,
**EPI_ISL_445206**	EPI1738564|A/Romania/1265/	Europe / Romania / Bucharest	2020-03-	A/Romania/1265/2020	National Institute for Infectious Diseases “Prof. Dr. Matei Balş”	National Institute of Infectious Diseases-Prof.	'Banica, Leontina; Surleac, Marius; Paraschiv,
**EPI_ISL_447873**	EPI1738791|Infl_20-	Europe / Sweden /	2020-03-	A/Sweden/86/2020	Public Health Agency of Sweden	Public Health Agency of Sweden	NA
**EPI_ISL_447876**	EPI1738808|Infl_20-	Europe / Sweden /	2020-03-	A/Sweden/89/2020	Public Health Agency of Sweden	Public Health Agency of Sweden	NA

**EPI_ISL_447877**	EPI1738816|Infl_20-	Europe / Sweden / Skane lan	2020-03-	A/Sweden/90/2020	Public Health Agency of Sweden	Public Health Agency of Sweden	NA
**EPI_ISL_447878**	EPI1738824|Infl_20-	Europe / Sweden / Hallands	2020-03-	A/Sweden/91/2020	Public Health Agency of Sweden	Public Health Agency of Sweden	NA
**EPI_ISL_447879**	EPI1738832|Infl_20-	Europe / Sweden / Hallands	2020-03-	A/Sweden/92/2020	Public Health Agency of Sweden	Public Health Agency of Sweden	NA
**EPI_ISL_447881**	EPI1738846|Infl_20-	Europe / Sweden / Skane lan	2020-03-	A/Sweden/95/2020	Public Health Agency of Sweden	Public Health Agency of Sweden	NA
**EPI_ISL_447882**	EPI1738854|Infl_20-	Europe / Sweden / Jonkopings	2020-03-	A/Sweden/96/2020	Public Health Agency of Sweden	Public Health Agency of Sweden	NA
**EPI_ISL_447883**	EPI1738862|Infl_20-	Europe / Sweden / Stockholms	2020-03-	A/Stockholm/32/2020	Klinisk mikrobiologi, Karolinska Universitetslaboratoriet, Karolinska	Public Health Agency of Sweden	NA
**EPI_ISL_447885**	EPI1738872|Infl_20-	Europe / Sweden / Skane lan	2020-03-	A/Sweden/97/2020	Public Health Agency of Sweden	Public Health Agency of Sweden	NA
**EPI_ISL_449731**	EPI1738896|3030724483_ZZ	North America / United States /	2020-03-	A/Minnesota/23/2020	Minnesota Department of Health	Centers for Disease Control and Prevention	NA
**EPI_ISL_449732**	EPI1738904|3030724482_ZZ	North America / United States /	2020-03-	A/Minnesota/24/2020	Minnesota Department of Health	Centers for Disease Control and Prevention	NA
**EPI_ISL_450392**	EPI1739402|S10038113.4	Oceania / Australia / Northern	2020	A/Darwin/141/2020	Influenza Surveillance Centre for Disease Control	WHO Collaborating Centre for Reference	Deng,Y-M; Iannello,P; Lau,H; Todd,A;
**EPI_ISL_453800**	EPI1740028|N10037967.4	Oceania / Australia / South	2019-07-	A/South	Institute of Medical and Veterinary Science (IMVS)	WHO Collaborating Centre for Reference	Deng,Y-M; Iannello,P; Lau,H; Todd,A;
**EPI_ISL_453807**	EPI1740084|N10037974.4	Asia / Philippines / National	2019-08-	A/Philippines/15/2019	Research Institute of Tropical Medicine	WHO Collaborating Centre for Reference	Deng,Y-M; Iannello,P; Lau,H; Todd,A;
**EPI_ISL_453810**	EPI1740108|N10037977.4	Oceania / Australia / New	2019-08-	A/Sydney/1200/2019	Institute of Medical and Veterinary Science (IMVS)	WHO Collaborating Centre for Reference	Deng,Y-M; Iannello,P; Lau,H; Todd,A;
**EPI_ISL_453812**	EPI1740129|N10037980.4	Oceania / Australia / New	2020-02-	A/Sydney/22/2020	Childrens Hospital Westmead	WHO Collaborating Centre for Reference	Deng,Y-M; Iannello,P; Lau,H; Todd,A;
**EPI_ISL_454745**	EPI1740228|A/NIIGATA/156/2	Asia / Japan	2020-02-	A/NIIGATA/156/2020	Niigata Prefectural Institute of Public Health and Environmental Sciences	National Institute of Infectious Diseases	Takashita,Emi;Fujisaki,Seiichiro;Shirakura,Masa
**EPI_ISL_454747**	EPI1740244|A/SAITAMA-	Asia / Japan	2020-02-	A/SAITAMA-	Saitama City Institute of Health Science and Research	National Institute of Infectious Diseases	Takashita,Emi;Fujisaki,Seiichiro;Shirakura,Masa
**EPI_ISL_455555**	EPI1740383|60-	Europe / France	2020-03-	A/Picardie/1840/2020	Institut Pasteur	Crick Worldwide Influenza Centre	NA
**EPI_ISL_455559**	EPI1740391|71-	Asia / Lebanon	2020-03-	A/Saida/361/2020	Rafic Hariri University Hospital 4th floor DNA/Research Lab	Crick Worldwide Influenza Centre	NA
**EPI_ISL_455563**	EPI1740398|46-	Europe / Switzerland	2020-03-	A/Switzerland/3278/20	Hopital Cantonal Universitaire de Geneves	Crick Worldwide Influenza Centre	NA
**EPI_ISL_455564**	EPI1740400|44-	Europe / Switzerland	2020-03-	A/Switzerland/8482/20	Hopital Cantonal Universitaire de Geneves	Crick Worldwide Influenza Centre	NA
**EPI_ISL_455797**	EPI1740466|3026055738_ZZ	North America / United States /	2020-03-	A/Illinois/10/2020	Illinois Department of Public Health-Carbondale	Centers for Disease Control and Prevention	NA
**EPI_ISL_455799**	EPI1740482|3026055825_ZZ	North America / United States /	2020-03-	A/Wisconsin/49/2020	Wisconsin State Laboratory of Hygiene	Centers for Disease Control and Prevention	NA
**EPI_ISL_455800**	EPI1740490|3026055826_ZZ	North America / United States /	2020-03-	A/Wisconsin/48/2020	Wisconsin State Laboratory of Hygiene	Centers for Disease Control and Prevention	NA
**EPI_ISL_459747**	EPI1741059|A_ENG_660_201	Europe / United Kingdom /	2019-12-	A/England/660/2019	Microbiology Services Colindale, Public Health England	Microbiology Services Colindale, Public	NA
**EPI_ISL_463766**	EPI1744596|A/Delaware/1197	North America / United States /	2020-03-	A/Delaware/11974/20	U.S. Air Force School of Aerospace Medicine	U.S. Air Force School of Aerospace	Gruner, W.E.; Fries, A.C.; Garrett, C.M.; Powell,
**EPI_ISL_464108**	EPI1745601|A/Alicante/19_10	Europe / Spain / Valencian	2020-03-	A/Alicante/19_1053_2	FISABIO - Public Health, Generalitat Valenciana	Center for Public Health Research (CSISP)	Lopez, F. Xavier; Diez, Javier
**EPI_ISL_467019**	EPI1745658|A/Finland/184/20	Europe / Finland / Lansi-	2020-03-	A/Finland/184/2020	National Institute for Health and Welfare	National Institute for Health and Welfare	NA
**EPI_ISL_468165**	EPI1745835|A/Finland/183/20	Europe / Finland / Etela-	2020-03-	A/Finland/183/2020	National Institute for Health and Welfare	National Institute for Health and Welfare	Ikonen, N; Haveri, A
**EPI_ISL_470884**	EPI1746272|A_ENG_2002205	Europe / United Kingdom /	2019-12-	A/England/200220520	Microbiology Services Colindale, Public Health England	Microbiology Services Colindale, Public	NA
**EPI_ISL_470931**	EPI1746510|A_ENG_2001001	Europe / United Kingdom /	2019-12-	A/England/200100148	Microbiology Services Colindale, Public Health England	Microbiology Services Colindale, Public	NA
**EPI_ISL_470933**	EPI1746526|A_ENG_916_201	Europe / United Kingdom /	2019-12-	A/England/916/2019	Microbiology Services Colindale, Public Health England	Microbiology Services Colindale, Public	NA
**EPI_ISL_471277**	EPI1747175|A/Saint-	Europe / Russian Federation /	2020-03-	A/Saint-Petersburg/RII-	Research Institute of Influenza, Ministry of Healthcare of the Russian	WHO National Influenza Centre Russian	NA
**EPI_ISL_471278**	EPI1747183|A/Saint-	Europe / Russian Federation /	2020-03-	A/Saint-Petersburg/RII-	Research Institute of Influenza, Ministry of Healthcare of the Russian	WHO National Influenza Centre Russian	NA
**EPI_ISL_471279**	EPI1747191|A/Saint-	Europe / Russian Federation /	2020-03-	A/Saint-Petersburg/RII-	Research Institute of Influenza, Ministry of Healthcare of the Russian	WHO National Influenza Centre Russian	NA
**EPI_ISL_471280**	EPI1747199|A/Saint-	Europe / Russian Federation /	2020-03-	A/Saint-Petersburg/RII-	Research Institute of Influenza, Ministry of Healthcare of the Russian	WHO National Influenza Centre Russian	NA
**EPI_ISL_471281**	EPI1747207|A/Saint-	Europe / Russian Federation /	2020-03-	A/Saint-Petersburg/RII-	Research Institute of Influenza, Ministry of Healthcare of the Russian	WHO National Influenza Centre Russian	NA
**EPI_ISL_471282**	EPI1747215|A/Saint-	Europe / Russian Federation /	2020-03-	A/Saint-Petersburg/RII-	Research Institute of Influenza, Ministry of Healthcare of the Russian	WHO National Influenza Centre Russian	NA
**EPI_ISL_471283**	EPI1747223|A/Saint-	Europe / Russian Federation /	2020-03-	A/Saint-Petersburg/RII-	Research Institute of Influenza, Ministry of Healthcare of the Russian	WHO National Influenza Centre Russian	NA
**EPI_ISL_471284**	EPI1747231|A/Saint-	Europe / Russian Federation /	2020-03-	A/Saint-Petersburg/RII-	Research Institute of Influenza, Ministry of Healthcare of the Russian	WHO National Influenza Centre Russian	NA
**EPI_ISL_471285**	EPI1747239|A/Saint-	Europe / Russian Federation /	2020-03-	A/Saint-Petersburg/RII-	Research Institute of Influenza, Ministry of Healthcare of the Russian	WHO National Influenza Centre Russian	NA
**EPI_ISL_471286**	EPI1747247|A/Saint-	Europe / Russian Federation /	2020-03-	A/Saint-Petersburg/RII-	Research Institute of Influenza, Ministry of Healthcare of the Russian	WHO National Influenza Centre Russian	NA
**EPI_ISL_471287**	EPI1747255|A/Saint-	Europe / Russian Federation /	2020-03-	A/Saint-Petersburg/RII-	Research Institute of Influenza, Ministry of Healthcare of the Russian	WHO National Influenza Centre Russian	NA
**EPI_ISL_471288**	EPI1747263|A/Saint-	Europe / Russian Federation /	2020-03-	A/Saint-Petersburg/RII-	Research Institute of Influenza, Ministry of Healthcare of the Russian	WHO National Influenza Centre Russian	NA
**EPI_ISL_471289**	EPI1747271|A/Saint-	Europe / Russian Federation /	2020-03-	A/Saint-Petersburg/RII-	Research Institute of Influenza, Ministry of Healthcare of the Russian	WHO National Influenza Centre Russian	NA
**EPI_ISL_471290**	EPI1747279|A/Saint-	Europe / Russian Federation /	2020-03-	A/Saint-Petersburg/RII-	Research Institute of Influenza, Ministry of Healthcare of the Russian	WHO National Influenza Centre Russian	NA
**EPI_ISL_471291**	EPI1747287|A/Saint-	Europe / Russian Federation /	2020-03-	A/Saint-Petersburg/RII-	Research Institute of Influenza, Ministry of Healthcare of the Russian	WHO National Influenza Centre Russian	NA
**EPI_ISL_471292**	EPI1747295|A/Saint-	Europe / Russian Federation /	2020-03-	A/Saint-Petersburg/RII-	Research Institute of Influenza, Ministry of Healthcare of the Russian	WHO National Influenza Centre Russian	NA
**EPI_ISL_471293**	EPI1747303|A/Saint-	Europe / Russian Federation /	2020-03-	A/Saint-Petersburg/RII-	Research Institute of Influenza, Ministry of Healthcare of the Russian	WHO National Influenza Centre Russian	NA
**EPI_ISL_471386**	EPI1748043|A/Saint-	Europe / Russian Federation /	2020-03-	A/Saint-Petersburg/RII-	Research Institute of Influenza, Ministry of Healthcare of the Russian	WHO National Influenza Centre Russian	NA
**EPI_ISL_471388**	EPI1748056|A/Saint-	Europe / Russian Federation /	2020-03-	A/Saint-Petersburg/RII-	Research Institute of Influenza, Ministry of Healthcare of the Russian	WHO National Influenza Centre Russian	NA
**EPI_ISL_471389**	EPI1748064|A/Saint-	Europe / Russian Federation /	2020-03-	A/Saint-Petersburg/RII-	Research Institute of Influenza, Ministry of Healthcare of the Russian	WHO National Influenza Centre Russian	NA
**EPI_ISL_471532**	EPI1748115|3000827611_N8	North America / Canada	2020-03-	A/Alberta/RV5268/202	National Microbiology Laboratory, Public Health Agency of Canada	Centers for Disease Control and Prevention	NA
**EPI_ISL_471533**	EPI1748123|3000827609_N8	North America / Canada	2020-03-	A/Manitoba/RV5046/2	National Microbiology Laboratory, Public Health Agency of Canada	Centers for Disease Control and Prevention	NA
**EPI_ISL_471534**	EPI1748131|3000827607_N8	North America / Canada	2020-03-	A/Ontario/RV4025/202	National Microbiology Laboratory, Public Health Agency of Canada	Centers for Disease Control and Prevention	NA
**EPI_ISL_471535**	EPI1748139|3000827604_N8	North America / Canada	2020-03-	A/Saskatchewan/RV3	National Microbiology Laboratory, Public Health Agency of Canada	Centers for Disease Control and Prevention	NA
**EPI_ISL_471536**	EPI1748147|3000827601_N8	North America / Canada	2020-03-	A/Quebec/RV3597/20	National Microbiology Laboratory, Public Health Agency of Canada	Centers for Disease Control and Prevention	NA
**EPI_ISL_471537**	EPI1748155|3000827618_N8	Europe / Russian Federation	2020-03-	A/St. Petersburg/RII-	Russian Academy of Medical Sciences	Centers for Disease Control and Prevention	NA
**EPI_ISL_471538**	EPI1748163|3000827617_N8	Europe / Russian Federation	2020-03-	A/St. Petersburg/RII-	Russian Academy of Medical Sciences	Centers for Disease Control and Prevention	NA
**EPI_ISL_477264**	EPI1748818|3000827579_N8	North America / Cayman	2020	A/Cayman	Caribbean Epidemiology Center	Centers for Disease Control and Prevention	NA
**EPI_ISL_477477**	EPI1749196|96-	Europe / Belgium	2020-02-	A/Belgium/S0831/202	Scientific Institute of Public Health	Crick Worldwide Influenza Centre	NA
**EPI_ISL_477537**	EPI1749316|72-	Europe / Iceland	2020-03-	A/Iceland/25/2020	Landspitali - University Hospital	Crick Worldwide Influenza Centre	NA
**EPI_ISL_477538**	EPI1749318|71-	Europe / Iceland	2020-03-	A/Iceland/26/2020	Landspitali - University Hospital	Crick Worldwide Influenza Centre	NA
**EPI_ISL_477539**	EPI1749320|69-	Europe / Iceland	2020-03-	A/Iceland/29/2020	Landspitali - University Hospital	Crick Worldwide Influenza Centre	NA
**EPI_ISL_477540**	EPI1749322|70-	Europe / Iceland	2020-03-	A/Iceland/30/2020	Landspitali - University Hospital	Crick Worldwide Influenza Centre	NA
**EPI_ISL_477543**	EPI1749328|68-	Europe / Iceland	2020-03-	A/Iceland/37/2020	Landspitali - University Hospital	Crick Worldwide Influenza Centre	NA
**EPI_ISL_477549**	EPI1749340|33-	Asia / Kyrgyzstan	2020-01-	A/Kyrgyzstan	National Virology Laboratory, Center Microbiological Investigations	Crick Worldwide Influenza Centre	NA
**EPI_ISL_477550**	EPI1749342|31-	Asia / Kyrgyzstan	2020-01-	A/Kyrgyzstan	National Virology Laboratory, Center Microbiological Investigations	Crick Worldwide Influenza Centre	NA
**EPI_ISL_477551**	EPI1749344|30-	Asia / Kyrgyzstan	2020-01-	A/Kyrgyzstan	National Virology Laboratory, Center Microbiological Investigations	Crick Worldwide Influenza Centre	NA

**EPI_ISL_477553**	EPI1749348|28-	Asia / Kyrgyzstan	2020-01-	A/Kyrgyzstan	National Virology Laboratory, Center Microbiological Investigations	Crick Worldwide Influenza Centre	NA
**EPI_ISL_477555**	EPI1749352|35-	Asia / Kyrgyzstan	2020-01-	A/Kyrgyzstan	National Virology Laboratory, Center Microbiological Investigations	Crick Worldwide Influenza Centre	NA
**EPI_ISL_477557**	EPI1749356|25-	Asia / Kyrgyzstan	2020-02-	A/Kyrgyzstan	National Virology Laboratory, Center Microbiological Investigations	Crick Worldwide Influenza Centre	NA
**EPI_ISL_477559**	EPI1749360|26-	Asia / Kyrgyzstan	2020-01-	A/Kyrgyzstan	National Virology Laboratory, Center Microbiological Investigations	Crick Worldwide Influenza Centre	NA
**EPI_ISL_477561**	EPI1749364|34-	Asia / Kyrgyzstan	2020-01-	A/Kyrgyzstan	National Virology Laboratory, Center Microbiological Investigations	Crick Worldwide Influenza Centre	NA
**EPI_ISL_477569**	EPI1749380|47-	Asia / Kyrgyzstan	2019-12-	A/Kyrgyzstan	National Virology Laboratory, Center Microbiological Investigations	Crick Worldwide Influenza Centre	NA
**EPI_ISL_477572**	EPI1749386|45-	Asia / Kyrgyzstan	2019-12-	A/Kyrgyzstan	National Virology Laboratory, Center Microbiological Investigations	Crick Worldwide Influenza Centre	NA
**EPI_ISL_477575**	EPI1749392|54-	Asia / Kyrgyzstan	2019-11-	A/Kyrgyzstan	National Virology Laboratory, Center Microbiological Investigations	Crick Worldwide Influenza Centre	NA
**EPI_ISL_477588**	EPI1749418|69-	Europe / Norway	2020-03-	A/Norway/2069/2020	WHO National Influenza Centre	Crick Worldwide Influenza Centre	NA
**EPI_ISL_477589**	EPI1749420|61-	Europe / Norway	2020-03-	A/Norway/2115/2020	WHO National Influenza Centre	Crick Worldwide Influenza Centre	NA
**EPI_ISL_477590**	EPI1749422|66-	Europe / Norway	2020-03-	A/Norway/2224/2020	WHO National Influenza Centre	Crick Worldwide Influenza Centre	NA
**EPI_ISL_477591**	EPI1749424|14-	Europe / Norway	2020-03-	A/Norway/2290/2020	WHO National Influenza Centre	Crick Worldwide Influenza Centre	NA
**EPI_ISL_477592**	EPI1749426|13-	Europe / Norway	2020-03-	A/Norway/2316/2020	WHO National Influenza Centre	Crick Worldwide Influenza Centre	NA
**EPI_ISL_477602**	EPI1749446|81-	Europe / Russian Federation	2020-03-	A/S.Petersburg/RII-	WHO National Influenza Centre Russian Federation	Crick Worldwide Influenza Centre	NA
**EPI_ISL_477610**	EPI1749462|82-	Europe / Russian Federation	2020-03-	A/Stavropol/10/2020	WHO National Influenza Centre Russian Federation	Crick Worldwide Influenza Centre	NA
**EPI_ISL_480451**	EPI1749614|A/ISHIKAWA/1/20	Asia / Japan	2020-01-	A/ISHIKAWA/1/2020	Ishikawa Prefectural Institute of Public Health and Environmental	National Institute of Infectious Diseases	Takashita,Emi;Fujisaki,Seiichiro;Shirakura,Masa
**EPI_ISL_480456**	EPI1749644|A/AICHI/98/2020_	Asia / Japan	2020-03-	A/AICHI/98/2020	Aichi Prefectural Institute of Public Health	National Institute of Infectious Diseases	Takashita,Emi;Fujisaki,Seiichiro;Shirakura,Masa
**EPI_ISL_480457**	EPI1749652|A_ENG_392_201	Europe / United Kingdom	2019-11-	A/England/392/2019	Microbiology Services Colindale, Public Health England	Microbiology Services Colindale, Public	NA
**EPI_ISL_480458**	EPI1749660|A_ENG_586_201	Europe / United Kingdom	2019-12-	A/England/586/2019	Microbiology Services Colindale, Public Health England	Microbiology Services Colindale, Public	NA
**EPI_ISL_481342**	EPI1750818|A/Singapore/KK2	Asia / Singapore	2020-01-	A/Singapore/KK272/2	Ministry of Health, Singapore	Ministry of Health, Singapore	Chen,B.;Zhou, Z.; Mak,T.M.; Cui,L; Lin,R.T.P.
**EPI_ISL_481456**	EPI1751098|HA_20-	Europe / Germany	2020-03-	A/Bremen/14/2020	Robert Koch-Institute Nationales Referenzzentrum für Influenza	Robert Koch Institute Nationales	NA
**EPI_ISL_481457**	EPI1751099|HA_20-	Europe / Germany	2020-03-	A/Nordrhein-	Robert Koch-Institute Nationales Referenzzentrum für Influenza	Robert Koch Institute Nationales	NA
**EPI_ISL_481458**	EPI1751100|HA_20-	Europe / Germany	2020-03-	A/Thueringen/65/2020	Robert Koch-Institute Nationales Referenzzentrum für Influenza	Robert Koch Institute Nationales	NA
**EPI_ISL_481459**	EPI1751101|HA_20-	Europe / Germany	2020-03-	A/Baden-	Robert Koch-Institute Nationales Referenzzentrum für Influenza	Robert Koch Institute Nationales	NA
**EPI_ISL_481460**	EPI1751102|HA_20-	Europe / Germany	2020-03-	A/Niedersachsen/90/2	Robert Koch-Institute Nationales Referenzzentrum für Influenza	Robert Koch Institute Nationales	NA
**EPI_ISL_481462**	EPI1751104|HA_20-	Europe / Germany	2020-03-	A/Schleswig-	Robert Koch-Institute Nationales Referenzzentrum für Influenza	Robert Koch Institute Nationales	NA
**EPI_ISL_481463**	EPI1751105|HA_20-	Europe / Germany	2020-03-	A/Rheinland-	Robert Koch-Institute Nationales Referenzzentrum für Influenza	Robert Koch Institute Nationales	NA
**EPI_ISL_481464**	EPI1751106|HA_20-	Europe / Germany	2020-03-	A/Hessen/34/2020	Robert Koch-Institute Nationales Referenzzentrum für Influenza	Robert Koch Institute Nationales	NA
**EPI_ISL_481465**	EPI1751107|HA_20-	Europe / Germany	2020-03-	A/Nordrhein-	Robert Koch-Institute Nationales Referenzzentrum für Influenza	Robert Koch Institute Nationales	NA
**EPI_ISL_481466**	EPI1751108|HA_20-	Europe / Germany	2020-03-	A/Nordrhein-	Robert Koch-Institute Nationales Referenzzentrum für Influenza	Robert Koch Institute Nationales	NA
**EPI_ISL_481467**	EPI1751109|HA_20-	Europe / Germany	2020-03-	A/Baden-	Robert Koch-Institute Nationales Referenzzentrum für Influenza	Robert Koch Institute Nationales	NA
**EPI_ISL_481468**	EPI1751110|HA_20-	Europe / Germany	2020-03-	A/Berlin/59/2020	Robert Koch-Institute Nationales Referenzzentrum für Influenza	Robert Koch Institute Nationales	NA
**EPI_ISL_481469**	EPI1751111|HA_20-	Europe / Germany	2020-03-	A/Niedersachsen/93/2	Robert Koch-Institute Nationales Referenzzentrum für Influenza	Robert Koch Institute Nationales	NA
**EPI_ISL_481470**	EPI1751112|HA_20-	Europe / Germany	2020-03-	A/Rheinland-	Robert Koch-Institute Nationales Referenzzentrum für Influenza	Robert Koch Institute Nationales	NA
**EPI_ISL_481471**	EPI1751113|HA_20-	Europe / Germany	2020-03-	A/Niedersachsen/136/	Robert Koch-Institute Nationales Referenzzentrum für Influenza	Robert Koch Institute Nationales	NA
**EPI_ISL_481472**	EPI1751114|HA_20-	Europe / Germany	2020-03-	A/Hessen/45/2020	Robert Koch-Institute Nationales Referenzzentrum für Influenza	Robert Koch Institute Nationales	NA
**EPI_ISL_481473**	EPI1751115|HA_20-	Europe / Germany	2020-03-	A/Brandenburg/14/202	Robert Koch-Institute Nationales Referenzzentrum für Influenza	Robert Koch Institute Nationales	NA
**EPI_ISL_481474**	EPI1751116|HA_20-	Europe / Germany	2020-03-	A/Berlin/65/2020	Robert Koch-Institute Nationales Referenzzentrum für Influenza	Robert Koch Institute Nationales	NA
**EPI_ISL_481475**	EPI1751117|HA_20-	Europe / Germany	2020-03-	A/Hessen/36/2020	Robert Koch-Institute Nationales Referenzzentrum für Influenza	Robert Koch Institute Nationales	NA
**EPI_ISL_481476**	EPI1751118|HA_20-	Europe / Germany	2020-03-	A/Baden-	Robert Koch-Institute Nationales Referenzzentrum für Influenza	Robert Koch Institute Nationales	NA
**EPI_ISL_481477**	EPI1751119|HA_20-	Europe / Germany	2020-03-	A/Hessen/46/2020	Robert Koch-Institute Nationales Referenzzentrum für Influenza	Robert Koch Institute Nationales	NA
**EPI_ISL_481478**	EPI1751120|HA_20-	Europe / Germany	2020-03-	A/Nordrhein-	Robert Koch-Institute Nationales Referenzzentrum für Influenza	Robert Koch Institute Nationales	NA
**EPI_ISL_481479**	EPI1751121|HA_20-	Europe / Germany	2020-03-	A/Niedersachsen/97/2	Robert Koch-Institute Nationales Referenzzentrum für Influenza	Robert Koch Institute Nationales	NA
**EPI_ISL_481480**	EPI1751122|HA_20-	Europe / Germany	2020-03-	A/Bayern/56/2020	Robert Koch-Institute Nationales Referenzzentrum für Influenza	Robert Koch Institute Nationales	NA
**EPI_ISL_481481**	EPI1751123|HA_20-	Europe / Germany	2020-03-	A/Bremen/10/2020	Robert Koch-Institute Nationales Referenzzentrum für Influenza	Robert Koch Institute Nationales	NA
**EPI_ISL_481482**	EPI1751124|HA_20-	Europe / Germany	2020-03-	A/Bremen/12/2020	Robert Koch-Institute Nationales Referenzzentrum für Influenza	Robert Koch Institute Nationales	NA
**EPI_ISL_482890**	EPI1751871|3000824211_N8	South America / Brazil	2020-02-	A/Minas	Instituto Oswaldo Cruz FIOCRUZ - Laboratory of Respiratory Viruses	Centers for Disease Control and Prevention	NA
**EPI_ISL_482893**	EPI1751895|3000824223_N8	South America / Brazil	2020-03-	A/Alagoas/496/2020	Instituto Oswaldo Cruz FIOCRUZ - Laboratory of Respiratory Viruses	Centers for Disease Control and Prevention	NA
**EPI_ISL_482894**	EPI1751903|3000824221_N8	South America / Brazil	2020-02-	A/Rio De	Instituto Oswaldo Cruz FIOCRUZ - Laboratory of Respiratory Viruses	Centers for Disease Control and Prevention	NA
**EPI_ISL_482895**	EPI1751911|3000824220_N8	South America / Brazil	2020-02-	A/Rio De	Instituto Oswaldo Cruz FIOCRUZ - Laboratory of Respiratory Viruses	Centers for Disease Control and Prevention	NA
**EPI_ISL_482896**	EPI1751919|3000824225_N8	South America / Brazil	2020-03-	A/Alagoas/552/2020	Instituto Oswaldo Cruz FIOCRUZ - Laboratory of Respiratory Viruses	Centers for Disease Control and Prevention	NA
**EPI_ISL_483574**	EPI1752480|S10038740.4	Oceania / Australia / Tasmania	2020-02-	A/Tasmania/503/2020	Hobart Pathology	WHO Collaborating Centre for Reference	Deng,Y-M; Iannello,P; Lau,H; Todd,A;
**EPI_ISL_483575**	EPI1752482|S10038741.4	Oceania / Australia / Western	2020-03-	A/Perth/45/2020	Pathwest QE II Medical Centre	WHO Collaborating Centre for Reference	Deng,Y-M; Iannello,P; Lau,H; Todd,A;
**EPI_ISL_485414**	EPI1752758|2020-CX0490.4	Asia / China / Gansu	2020-02-	A/Gansu-	WHO Chinese National Influenza Center	WHO Chinese National Influenza Center	Xiaoxu,Zeng;Xiyan,Li;Weijuan,Huang;Lei,Yang;D
**EPI_ISL_485415**	EPI1752761|2020-CX0489.4	Asia / China / Neimenggu	2020-03-	A/Neimenggu-	WHO Chinese National Influenza Center	WHO Chinese National Influenza Center	Xiaoxu,Zeng;Xiyan,Li;Weijuan,Huang;Lei,Yang;D
**EPI_ISL_485416**	EPI1752764|2020-CX0488.4	Asia / China / Yunnan	2020-03-	A/Yunnan-	WHO Chinese National Influenza Center	WHO Chinese National Influenza Center	Xiaoxu,Zeng;Xiyan,Li;Weijuan,Huang;Lei,Yang;D
**EPI_ISL_485417**	EPI1752767|2020-CX0487.4	Asia / China / Liaoning	2020-03-	A/Liaoning-	WHO Chinese National Influenza Center	WHO Chinese National Influenza Center	Xiaoxu,Zeng;Xiyan,Li;Weijuan,Huang;Lei,Yang;D
**EPI_ISL_485419**	EPI1752773|2020-CX0484.4	Asia / China / Heilongjiang	2020-03-	A/Heilongjiang-	WHO Chinese National Influenza Center	WHO Chinese National Influenza Center	Xiaoxu,Zeng;Xiyan,Li;Weijuan,Huang;Lei,Yang;D
**EPI_ISL_485458**	EPI1752890|2020-CX0446.4	Asia / China / Guangdong	2020-01-	A/Guangdong-	WHO Chinese National Influenza Center	WHO Chinese National Influenza Center	Xiaoxu,Zeng;Xiyan,Li;Weijuan,Huang;Lei,Yang;D
**EPI_ISL_485465**	EPI1752911|2020-CX0439.4	Asia / China / Liaoning	2020-03-	A/Liaoning-	WHO Chinese National Influenza Center	WHO Chinese National Influenza Center	Xiaoxu,Zeng;Xiyan,Li;Weijuan,Huang;Lei,Yang;D
**EPI_ISL_485466**	EPI1752914|2020-CX0438.4	Asia / China / Shaanxi	2020-02-	A/Shaanxi-	WHO Chinese National Influenza Center	WHO Chinese National Influenza Center	Xiaoxu,Zeng;Xiyan,Li;Weijuan,Huang;Lei,Yang;D
**EPI_ISL_485467**	EPI1752917|2020-CX0437.4	Asia / China	2020-02-	A/Liaoning-	WHO Chinese National Influenza Center	WHO Chinese National Influenza Center	Xiaoxu,Zeng;Xiyan,Li;Weijuan,Huang;Lei,Yang;D
**EPI_ISL_485468**	EPI1752920|2020-CX0436.4	Asia / China / Neimenggu	2020-02-	A/Neimenggu-	WHO Chinese National Influenza Center	WHO Chinese National Influenza Center	Xiaoxu,Zeng;Xiyan,Li;Weijuan,Huang;Lei,Yang;D
**EPI_ISL_485469**	EPI1752923|2020-CX0435.4	Asia / China / Hunan	2020-02-	A/Hunan-	WHO Chinese National Influenza Center	WHO Chinese National Influenza Center	Xiaoxu,Zeng;Xiyan,Li;Weijuan,Huang;Lei,Yang;D
**EPI_ISL_485470**	EPI1752926|2020-CX0434.4	Asia / China / Yunnan	2020-02-	A/Yunnan-	WHO Chinese National Influenza Center	WHO Chinese National Influenza Center	Xiaoxu,Zeng;Xiyan,Li;Weijuan,Huang;Lei,Yang;D
**EPI_ISL_485471**	EPI1752929|2020-CX0433.4	Asia / China / Guangdong	2020-02-	A/Guangdong-	WHO Chinese National Influenza Center	WHO Chinese National Influenza Center	Xiaoxu,Zeng;Xiyan,Li;Weijuan,Huang;Lei,Yang;D
**EPI_ISL_485472**	EPI1752932|2020-CX0432.4	Asia / China / Guangxi	2020-01-	A/Guangxi-	WHO Chinese National Influenza Center	WHO Chinese National Influenza Center	Xiaoxu,Zeng;Xiyan,Li;Weijuan,Huang;Lei,Yang;D
**EPI_ISL_485473**	EPI1752935|2020-CX0431.4	Asia / China / Yunnan	2020-02-	A/Yunnan-	WHO Chinese National Influenza Center	WHO Chinese National Influenza Center	Xiaoxu,Zeng;Xiyan,Li;Weijuan,Huang;Lei,Yang;D

**EPI_ISL_485475**	EPI1752941|2020-CX0429.4	Asia / China / Yunnan	2020-03-	A/Yunnan-	WHO Chinese National Influenza Center	WHO Chinese National Influenza Center	Xiaoxu,Zeng;Xiyan,Li;Weijuan,Huang;Lei,Yang;D
**EPI_ISL_485476**	EPI1752944|2020-CX0428.4	Asia / China / Neimenggu	2020-03-	A/Neimenggu-	WHO Chinese National Influenza Center	WHO Chinese National Influenza Center	Xiaoxu,Zeng;Xiyan,Li;Weijuan,Huang;Lei,Yang;D
**EPI_ISL_485477**	EPI1752947|2020-CX0427.4	Asia / China	2020-03-	A/Liaoning-	WHO Chinese National Influenza Center	WHO Chinese National Influenza Center	Xiaoxu,Zeng;Xiyan,Li;Weijuan,Huang;Lei,Yang;D
**EPI_ISL_485512**	EPI1753052|2020-CX0354.4	Asia / Korea, Republic of	2019-11-	A/Jeonnam/1305/201	WHO Chinese National Influenza Center	WHO Chinese National Influenza Center	Xiaoxu,Zeng;Xiyan,Li;Weijuan,Huang;Lei,Yang;D
**EPI_ISL_485515**	EPI1753061|2020-CX0351.4	Asia / Korea, Republic of /	2019-11-	A/Incheon/1286/2019	WHO Chinese National Influenza Center	WHO Chinese National Influenza Center	Xiaoxu,Zeng;Xiyan,Li;Weijuan,Huang;Lei,Yang;D
**EPI_ISL_485516**	EPI1753064|2020-CX0350.4	Asia / Korea, Republic of	2019-10-	A/Gyeongbuk/1271/20	WHO Chinese National Influenza Center	WHO Chinese National Influenza Center	Xiaoxu,Zeng;Xiyan,Li;Weijuan,Huang;Lei,Yang;D
**EPI_ISL_485911**	EPI1753631|2020-CX0609.4	Asia / China / Beijing	2020-01-	A/Beijing-	WHO Chinese National Influenza Center	WHO Chinese National Influenza Center	Li,Xin;Dayan,Wang
**EPI_ISL_486683**	EPI1754012|72-	Europe / Cyprus	2020-02-	A/Cyprus/F1267/2020	Nicosia General Hospital	Crick Worldwide Influenza Centre	NA
**EPI_ISL_486688**	EPI1754022|72-	Europe / Cyprus	2020-02-	A/Cyprus/F926/2020	Nicosia General Hospital	Crick Worldwide Influenza Centre	NA
**EPI_ISL_486766**	EPI1754177|76-	Europe / Switzerland	2020-03-	A/Switzerland/6206/20	Hopital Cantonal Universitaire de Geneves	Crick Worldwide Influenza Centre	NA
**EPI_ISL_487058**	EPI1754785|A_ENG_2005806	Europe / United Kingdom /	2020-01-	A/England/200580698	Microbiology Services Colindale, Public Health England	Microbiology Services Colindale, Public	NA
**EPI_ISL_487073**	EPI1754923|A_ENG_2008204	Europe / United Kingdom /	2020-02-	A/England/200820416	Microbiology Services Colindale, Public Health England	Microbiology Services Colindale, Public	NA
**EPI_ISL_487232**	EPI1755653|LNS6274702_HA	Europe / Luxembourg	2020-03-	A/Luxembourg/LNS62	Laboratoire National de Santé	Laboratoire National de Santé	Wienecke-Baldacchino, Anke; Fournier,
**EPI_ISL_487233**	EPI1755661|LNS8654682_HA	Europe / Luxembourg	2020-03-	A/Luxembourg/LNS86	Laboratoire National de Santé	Laboratoire National de Santé	Wienecke-Baldacchino, Anke; Fournier,
**EPI_ISL_487237**	EPI1755693|LNS8249633_HA	Europe / Luxembourg	2020-03-	A/Luxembourg/LNS82	Laboratoire National de Santé	Laboratoire National de Santé	Wienecke-Baldacchino, Anke; Fournier,
**EPI_ISL_487239**	EPI1755709|LNS4363553_HA	Europe / Luxembourg	2020-02-	A/Luxembourg/LNS43	Laboratoire National de Santé	Laboratoire National de Santé	Wienecke-Baldacchino, Anke; Fournier,
**EPI_ISL_487240**	EPI1755717|LNS1245335_HA	Europe / Luxembourg	2020-03-	A/Luxembourg/LNS12	Laboratoire National de Santé	Laboratoire National de Santé	Wienecke-Baldacchino, Anke; Fournier,
**EPI_ISL_487242**	EPI1755733|LNS8679840_HA	Europe / Luxembourg	2020-02-	A/Luxembourg/LNS86	Laboratoire National de Santé	Laboratoire National de Santé	Wienecke-Baldacchino, Anke; Fournier,
**EPI_ISL_487472**	EPI1755907|3000824295_N8	North America / Guadeloupe	2020-03-	A/Guadeloupe/447/20	National Influenza Center French Guiana and French Indies	Centers for Disease Control and Prevention	NA
**EPI_ISL_487473**	EPI1755915|3000824296_N8	North America / Guadeloupe	2020-02-	A/Guadeloupe/451/20	National Influenza Center French Guiana and French Indies	Centers for Disease Control and Prevention	NA
**EPI_ISL_487474**	EPI1755923|3000824228_N8	South America / Brazil	2020-03-	A/Rio De	Instituto Oswaldo Cruz FIOCRUZ - Laboratory of Respiratory Viruses	Centers for Disease Control and Prevention	NA
**EPI_ISL_490877**	EPI1756786|A/Colorado/1210	North America / United States /	2020-03-	A/Colorado/12106/202	U.S. Air Force School of Aerospace Medicine	U.S. Air Force School of Aerospace	Gruner, W.E., Fries, A.C., Garrett, C.M., Powell,
**EPI_ISL_490880**	EPI1756802|A/Delaware/1210	North America / United States /	2020-03-	A/Delaware/12109/20	U.S. Air Force School of Aerospace Medicine	U.S. Air Force School of Aerospace	Gruner, W.E., Fries, A.C., Garrett, C.M., Powell,
**EPI_ISL_490881**	EPI1756810|A/Delaware/1211	North America / United States /	2020-03-	A/Delaware/12110/20	U.S. Air Force School of Aerospace Medicine	U.S. Air Force School of Aerospace	Gruner, W.E., Fries, A.C., Garrett, C.M., Powell,
**EPI_ISL_490912**	EPI1757010|A/Ohio/12141/20	North America / United States /	2020-03-	A/Ohio/12141/2020	U.S. Air Force School of Aerospace Medicine	U.S. Air Force School of Aerospace	Gruner, W.E., Fries, A.C., Garrett, C.M., Powell,
**EPI_ISL_491766**	EPI1757829|3001289930_N8	Africa / Togo	2019-09-	A/Togo/1146/2019	Institute National D'Hygiene	Centers for Disease Control and Prevention	NA
**EPI_ISL_491771**	EPI1757869|3000826201_N8	Africa / Togo	2019-12-	A/Togo/1942/2019	Institute National D'Hygiene	Centers for Disease Control and Prevention	NA
**EPI_ISL_491772**	EPI1757877|3000826202_N8	Africa / Togo	2019-12-	A/Togo/1973/2019	Institute National D'Hygiene	Centers for Disease Control and Prevention	NA
**EPI_ISL_491775**	EPI1757901|3000826191_N8	Africa / Togo	2019-11-	A/Togo/1925/2019	Institute National D'Hygiene	Centers for Disease Control and Prevention	NA
**EPI_ISL_491777**	EPI1757917|3000826182_N8	Africa / Togo	2019-11-	A/Togo/1784/2019	Institute National D'Hygiene	Centers for Disease Control and Prevention	NA
**EPI_ISL_491778**	EPI1757925|3000826181_N8	Africa / Togo	2019-11-	A/Togo/1697/2019	Institute National D'Hygiene	Centers for Disease Control and Prevention	NA
**EPI_ISL_491783**	EPI1757963|3000826165_N8	Africa / Togo	2019-11-	A/Togo/1654/2019	Institute National D'Hygiene	Centers for Disease Control and Prevention	NA
**EPI_ISL_491796**	EPI1758064|3000827509_N8	Africa / Nigeria	2019-08-	A/Nigeria/4084/2019	NCDC Public Health Reference Laboratory	Centers for Disease Control and Prevention	NA
**EPI_ISL_491803**	EPI1758113|3001292350_N8	Asia / Bangladesh	2019-10-	A/Bangladesh/18-	Institute of Epidemiology Disease Control and Research (IEDCR) &	Centers for Disease Control and Prevention	NA
**EPI_ISL_491808**	EPI1758153|3000826552_N8	Africa / Mali	2019-10-	A/Mali/167/2019	NIC Lab CVD-MALI	Centers for Disease Control and Prevention	NA
**EPI_ISL_491810**	EPI1758169|3000826546_N8	Africa / Mali	2019-10-	A/Mali/166/2019	NIC Lab CVD-MALI	Centers for Disease Control and Prevention	NA
**EPI_ISL_491811**	EPI1758177|3000826544_N8	Africa / Mali	2019-10-	A/Mali/164/2019	NIC Lab CVD-MALI	Centers for Disease Control and Prevention	NA
**EPI_ISL_491831**	EPI1758332|3001289944_N8	Africa / Togo	2019-09-	A/Togo/1255/2019	Institute National D'Hygiene	Centers for Disease Control and Prevention	NA
**EPI_ISL_493142**	EPI1759260|S10038995.4	Oceania / Australia /	2020-03-	A/Brisbane/14/2020	Queensland Health Forensic and Scientific Services	WHO Collaborating Centre for Reference	Deng,Y-M; Iannello,P; Lau,H; Todd,A;
**EPI_ISL_493143**	EPI1759263|S10038999.4	Oceania / Australia /	2020-01-	A/Townsville/4/2020	Queensland Health Forensic and Scientific Services	WHO Collaborating Centre for Reference	Deng,Y-M; Iannello,P; Lau,H; Todd,A;
**EPI_ISL_493144**	EPI1759266|S10039000.4	Oceania / Australia / Victoria /	2020-02-	A/Victoria/134/2020	Alfred Hospital	WHO Collaborating Centre for Reference	Deng,Y-M; Iannello,P; Lau,H; Todd,A;
**EPI_ISL_493147**	EPI1759275|S10039166.4	Asia / Indonesia	2019-11-	A/Indonesia/NIHRDSB	Ministry of Health, NIHRD	WHO Collaborating Centre for Reference	Siswanto,S; Setiawaty,V; Deng, Y-M;
**EPI_ISL_493148**	EPI1759278|S10039167.4	Asia / Malaysia	2020	A/Malaysia/RP0961/20	Institut Penyelidikan Perubatan	WHO Collaborating Centre for Reference	Deng,Y-M; Iannello,P; Lau,H; Todd,A;
**EPI_ISL_497911**	EPI1763037|A/Novosibirsk/RII-	Europe / Russian Federation /	2020-02-	A/Novosibirsk/RII-	Research Institute of Experimental and Clinical Medicine	WHO National Influenza Centre Russian	NA
**EPI_ISL_498216**	EPI1763497|A/GUNMA/39/202	Asia / Japan	2020-02-	A/GUNMA/39/2020	Gunma Prefectural Institute of Public Health and Environmental	National Institute of Infectious Diseases	Takashita,Emi;Fujisaki,Seiichiro;Shirakura,Masa
**EPI_ISL_498217**	EPI1763505|A/GUNMA/50/202	Asia / Japan	2020-02-	A/GUNMA/50/2020	Gunma Prefectural Institute of Public Health and Environmental	National Institute of Infectious Diseases	Takashita,Emi;Fujisaki,Seiichiro;Shirakura,Masa
**EPI_ISL_498218**	EPI1763513|A/OSAKA-	Asia / Japan	2020-02-	A/OSAKA-	Osaka City Institute of Public Health and Environmental Sciences	National Institute of Infectious Diseases	Takashita,Emi;Fujisaki,Seiichiro;Shirakura,Masa
**EPI_ISL_498298**	EPI1763589|19-	Europe / France	2020-03-	A/Centre/2293/2020	Institut Pasteur	Crick Worldwide Influenza Centre	NA
**EPI_ISL_498349**	EPI1763690|84-	Europe / France	2020-03-	A/Lyon/1631/2020	CNR Virus des Infections Respiratoires - France SUD	Crick Worldwide Influenza Centre	NA
**EPI_ISL_498357**	EPI1763706|20-	Europe / France	2020-03-	A/Lyon/1826/2020	CNR Virus des Infections Respiratoires - France SUD	Crick Worldwide Influenza Centre	NA
**EPI_ISL_498391**	EPI1763772|23-	Europe / Portugal	2020-03-	A/Portugal/MS74/2020	Instituto Nacional de Saude (INSA)	Crick Worldwide Influenza Centre	NA
**EPI_ISL_499050**	EPI1765568|3000824331_N8	South America / Chile	2020-01-	A/Santiago/4891/2020	Instituto de Salud Publica de Chile	Centers for Disease Control and Prevention	NA
**EPI_ISL_499051**	EPI1765575|3000824332_N8	South America / Chile	2020-02-	A/Punta	Instituto de Salud Publica de Chile	Centers for Disease Control and Prevention	NA
**EPI_ISL_499052**	EPI1765583|3000824333_N8	South America / Chile	2020-03-	A/Santiago/26038/202	Instituto de Salud Publica de Chile	Centers for Disease Control and Prevention	NA
**EPI_ISL_500726**	EPI1766858|48-	Europe / Cyprus	2020-03-	A/Cyprus/F1305/2020	Nicosia General Hospital	Crick Worldwide Influenza Centre	NA
**EPI_ISL_500732**	EPI1766870|84-	Europe / Estonia	2020-03-	A/Estonia/126846/202	Health Protection Inspectorate	Crick Worldwide Influenza Centre	NA
**EPI_ISL_500733**	EPI1766872|83-	Europe / Estonia	2020-03-	A/Estonia/127241/202	Health Protection Inspectorate	Crick Worldwide Influenza Centre	NA
**EPI_ISL_500734**	EPI1766874|77-	Europe / Norway	2020-03-	A/Norway/1900/2020	WHO National Influenza Centre	Crick Worldwide Influenza Centre	NA
**EPI_ISL_500735**	EPI1766876|76-	Europe / Norway	2020-03-	A/Norway/1906/2020	WHO National Influenza Centre	Crick Worldwide Influenza Centre	NA
**EPI_ISL_500738**	EPI1766882|69-	Europe / Norway	2020-03-	A/Norway/2174/2020	WHO National Influenza Centre	Crick Worldwide Influenza Centre	NA
**EPI_ISL_500739**	EPI1766884|14-	Europe / Norway	2020-03-	A/Norway/2176/2020	WHO National Influenza Centre	Crick Worldwide Influenza Centre	NA
**EPI_ISL_500740**	EPI1766886|72-	Europe / Norway	2020-03-	A/Norway/2205/2020	WHO National Influenza Centre	Crick Worldwide Influenza Centre	NA
**EPI_ISL_500741**	EPI1766888|73-	Europe / Norway	2020-03-	A/Norway/2208/2020	WHO National Influenza Centre	Crick Worldwide Influenza Centre	NA
**EPI_ISL_500742**	EPI1766890|71-	Europe / Norway	2020-03-	A/Norway/2228/2020	WHO National Influenza Centre	Crick Worldwide Influenza Centre	NA
**EPI_ISL_500744**	EPI1766894|67-	Europe / Norway	2020-03-	A/Norway/2292/2020	WHO National Influenza Centre	Crick Worldwide Influenza Centre	NA
**EPI_ISL_500745**	EPI1766896|64-	Europe / Norway	2020-03-	A/Norway/2310/2020	WHO National Influenza Centre	Crick Worldwide Influenza Centre	NA
**EPI_ISL_500746**	EPI1766898|65-	Europe / Norway	2020-03-	A/Norway/2320/2020	WHO National Influenza Centre	Crick Worldwide Influenza Centre	NA
**EPI_ISL_500747**	EPI1766900|63-	Europe / Norway	2020-03-	A/Norway/2357/2020	WHO National Influenza Centre	Crick Worldwide Influenza Centre	NA

**EPI_ISL_510020**	EPI1795461|N10039349.4	Oceania / Australia /	2020-03-	A/Brisbane/15/2020	Queensland Health Forensic and Scientific Services	WHO Collaborating Centre for Reference	Deng,Y-M; Iannello,P; Lau,H; Todd,A;
**EPI_ISL_510021**	EPI1795464|N10039350.4	Africa / South Africa / KwaZulu	2019-07-	A/South	National Institute for Communicable Disease	WHO Collaborating Centre for Reference	Deng,Y-M; Iannello,P; Lau,H; Todd,A;
**EPI_ISL_514677**	EPI1797803|N10039637.4	Asia / Cambodia / Kratie	2020-03-	A/Cambodia/FSS4291	Institute Pasteur du Cambodia	WHO Collaborating Centre for Reference	Deng,Y-M; Iannello,P; Lau,H; Todd,A;
**EPI_ISL_514678**	EPI1797806|N10039638.4	Asia / Cambodia / Battambang	2020-03-	A/Cambodia/FSS4293	Institute Pasteur du Cambodia	WHO Collaborating Centre for Reference	Deng,Y-M; Iannello,P; Lau,H; Todd,A;
**EPI_ISL_514680**	EPI1797812|N10039640.4	Asia / Cambodia / Khett Kandal	2020-01-	A/Cambodia/FSS4236	Institute Pasteur du Cambodia	WHO Collaborating Centre for Reference	Deng,Y-M; Iannello,P; Lau,H; Todd,A;
**EPI_ISL_514681**	EPI1797815|N10039642.4	Asia / Cambodia / Oddor Mean	2020-03-	A/Cambodia/e040336	Institute Pasteur du Cambodia	WHO Collaborating Centre for Reference	Deng,Y-M; Iannello,P; Lau,H; Todd,A;
**EPI_ISL_514682**	EPI1797818|N10039643.4	Asia / Cambodia / Khett Svay	2020-02-	A/Cambodia/e030437	Institute Pasteur du Cambodia	WHO Collaborating Centre for Reference	Deng,Y-M; Iannello,P; Lau,H; Todd,A;
**EPI_ISL_514683**	EPI1797821|N10039644.4	Asia / Cambodia / Khett	2020-03-	A/Cambodia/e040337	Institute Pasteur du Cambodia	WHO Collaborating Centre for Reference	Deng,Y-M; Iannello,P; Lau,H; Todd,A;
**EPI_ISL_514689**	EPI1797839|N10039650.4	Asia / Cambodia / Mundulkiri	2020-04-	A/Cambodia/e051238	Institute Pasteur du Cambodia	WHO Collaborating Centre for Reference	Deng,Y-M; Iannello,P; Lau,H; Todd,A;
**EPI_ISL_514690**	EPI1797842|N10039651.4	Asia / Cambodia / Siem Reap	2020-03-	A/Cambodia/e051238	Institute Pasteur du Cambodia	WHO Collaborating Centre for Reference	Deng,Y-M; Iannello,P; Lau,H; Todd,A;
**EPI_ISL_514701**	EPI1797875|N10039663.4	Asia / Cambodia / Oddor Mean	2020-02-	A/Cambodia/e022636	Institute Pasteur du Cambodia	WHO Collaborating Centre for Reference	Deng,Y-M; Iannello,P; Lau,H; Todd,A;
**EPI_ISL_514702**	EPI1797878|N10039664.4	Asia / Cambodia / Phnom Penh	2020-03-	A/Cambodia/e040338	Institute Pasteur du Cambodia	WHO Collaborating Centre for Reference	Deng,Y-M; Iannello,P; Lau,H; Todd,A;
**EPI_ISL_514703**	EPI1797881|N10039665.4	Asia / Cambodia / Mundulkiri	2020-03-	A/Cambodia/e040338	Institute Pasteur du Cambodia	WHO Collaborating Centre for Reference	Deng,Y-M; Iannello,P; Lau,H; Todd,A;
**EPI_ISL_514704**	EPI1797884|N10039666.4	Asia / Cambodia / Khett	2020-01-	A/Cambodia/e020637	Institute Pasteur du Cambodia	WHO Collaborating Centre for Reference	Deng,Y-M; Iannello,P; Lau,H; Todd,A;
**EPI_ISL_514708**	EPI1797896|N10039670.4	Asia / Cambodia / Khett Svay	2020-03-	A/Cambodia/e040338	Institute Pasteur du Cambodia	WHO Collaborating Centre for Reference	Deng,Y-M; Iannello,P; Lau,H; Todd,A;
**EPI_ISL_514709**	EPI1797899|N10039671.4	Asia / Brunei	2020-02-	A/Brunei/48/2020	R.I.P.A.S. Hospital, Department of Laboratory Services	WHO Collaborating Centre for Reference	Deng,Y-M; Iannello,P; Lau,H; Todd,A;
**EPI_ISL_514712**	EPI1797908|N10039674.4	Asia / Cambodia / Palin	2020-03-	A/Cambodia/e032736	Institute Pasteur du Cambodia	WHO Collaborating Centre for Reference	Deng,Y-M; Iannello,P; Lau,H; Todd,A;
**EPI_ISL_514713**	EPI1797911|N10039675.4	Asia / Cambodia / Oddor Mean	2020-03-	A/Cambodia/e032736	Institute Pasteur du Cambodia	WHO Collaborating Centre for Reference	Deng,Y-M; Iannello,P; Lau,H; Todd,A;
**EPI_ISL_514718**	EPI1797926|N10039680.4	Asia / Malaysia	2019-12-	A/Malaysia/RP0701/20	Institut Penyelidikan Perubatan	WHO Collaborating Centre for Reference	Deng,Y-M; Iannello,P; Lau,H; Todd,A;
**EPI_ISL_514719**	EPI1797929|N10039682.4	Oceania / Australia /	2020-03-	A/Brisbane/17/2020	Queensland Health Forensic and Scientific Services	WHO Collaborating Centre for Reference	Deng,Y-M; Iannello,P; Lau,H; Todd,A;
**EPI_ISL_515539**	EPI1798556|A/Bretagne/2248/	Europe / France / Bretagne	2020-03-	A/Bretagne/2248/2020	Institut Pasteur	Institut Pasteur	NA
**EPI_ISL_516098**	EPI1798777|416-20_HA	South America / Brazil / Estado	2020-03-	A/Rio de	Laboratório Central de Saúde Pública (LACEN RJ)	Instituto Oswaldo Cruz FIOCRUZ -	Resende,P.; Motta,F.; Caetano,B.;
**EPI_ISL_516110**	EPI1798810|965-19_HA	South America / Brazil / Estado	2019-09-	A/Santa	LACEN SC	Instituto Oswaldo Cruz FIOCRUZ -	Resende,P.; Motta,F.; Caetano,B.;
**EPI_ISL_516113**	EPI1798827|1020-19_HA	South America / Brazil / Estado	2019-10-	A/Santa	LACEN SC	Instituto Oswaldo Cruz FIOCRUZ -	Resende,P.; Motta,F.; Caetano,B.;
**EPI_ISL_516134**	EPI1798862|1067-19_HA	South America / Brazil / Estado	2019-09-	A/Rio Grande do	LACEN/RS - Laboratório Central de Saúde Pública do Rio Grande do	Instituto Oswaldo Cruz FIOCRUZ -	Resende,P.; Motta,F.; Caetano,B.;
**EPI_ISL_516135**	EPI1798870|26-20_HA	South America / Brazil / Estado	2020-01-	A/Rio Grande do	LACEN/RS - Laboratório Central de Saúde Pública do Rio Grande do	Instituto Oswaldo Cruz FIOCRUZ -	Resende,P.; Motta,F.; Caetano,B.;
**EPI_ISL_516138**	EPI1798880|122-20_HA	South America / Brazil / Estado	2020-02-	A/Rio Grande do	LACEN/RS - Laboratório Central de Saúde Pública do Rio Grande do	Instituto Oswaldo Cruz FIOCRUZ -	Resende,P.; Motta,F.; Caetano,B.;
**EPI_ISL_516140**	EPI1798889|212-20_HA	South America / Brazil / Estado	2020-02-	A/Rio Grande do	LACEN/RS - Laboratório Central de Saúde Pública do Rio Grande do	Instituto Oswaldo Cruz FIOCRUZ -	Resende,P.; Motta,F.; Caetano,B.;
**EPI_ISL_516141**	EPI1798897|988-19_HA	South America / Brazil / Estado	2019-09-	A/Parana/988/2019	Laboratório Central do Estado do Paraná - LACEN/PR	Instituto Oswaldo Cruz FIOCRUZ -	Resende,P.; Motta,F.; Caetano,B.;
**EPI_ISL_516144**	EPI1798907|41-20_HA	South America / Brazil / Estado	2020-01-	A/Parana/41/2020	Laboratório Central do Estado do Paraná - LACEN/PR	Instituto Oswaldo Cruz FIOCRUZ -	Resende,P.; Motta,F.; Caetano,B.;
**EPI_ISL_516148**	EPI1798918|51-20_HA	South America / Brazil / Estado	2020-01-	A/Parana/51/2020	Laboratório Central do Estado do Paraná - LACEN/PR	Instituto Oswaldo Cruz FIOCRUZ -	Resende,P.; Motta,F.; Caetano,B.;
**EPI_ISL_516150**	EPI1798927|1990-20_HA	South America / Brazil / Estado	2020-03-	A/Parana/1990/2020	Laboratório Central do Estado do Paraná - LACEN/PR	Instituto Oswaldo Cruz FIOCRUZ -	Resende,P.; Motta,F.; Caetano,B.;
**EPI_ISL_516154**	EPI1798938|1994-20_HA	South America / Brazil / Estado	2020-03-	A/Parana/1994/2020	Laboratório Central do Estado do Paraná - LACEN/PR	Instituto Oswaldo Cruz FIOCRUZ -	Resende,P.; Motta,F.; Caetano,B.;
**EPI_ISL_516175**	EPI1798966|191-20_HA	South America / Brazil / Estado	2020-02-	A/Espirito	LACEN/ES - Laboratório Central de Saúde Pública do Espírito Santo	Instituto Oswaldo Cruz FIOCRUZ -	Resende,P.; Motta,F.; Caetano,B.;
**EPI_ISL_516789**	EPI1798991|A/Romania/2592	Europe / Romania / Judetul Olt	2020-03-	A/Romania/259277/20	Cantacuzino Institute	Cantacuzino Institute	NA
**EPI_ISL_516796**	EPI1799041|A/Romania/2598	Europe / Romania / Judetul Iasi	2020-03-	A/Romania/259803/20	Cantacuzino Institute	Cantacuzino Institute	Luiza,Ustea; Nicoleta,Paraschiv; Mihaela,Lazar
**EPI_ISL_516797**	EPI1799048|A/Romania/2598	Europe / Romania / Judetul Iasi	2020-03-	A/Romania/259804/20	Cantacuzino Institute	Cantacuzino Institute	Luiza,Ustea; Nicoleta,Paraschiv; Mihaela,Lazar
**EPI_ISL_522425**	EPI1799932|2020-CX0656.4	Asia / China / Fujian	2020-04-	A/Fujian-	WHO Chinese National Influenza Center	WHO Chinese National Influenza Center	Xin li,Li Xiyan, Dayan Wang, Zeng Xiaoxu
**EPI_ISL_522427**	EPI1799938|2020-CX0653.4	Asia / China / Yunnan	2020-05-	A/Yunnan-	WHO Chinese National Influenza Center	WHO Chinese National Influenza Center	Xin li,Li Xiyan, Dayan Wang, Zeng Xiaoxu
**EPI_ISL_522428**	EPI1799941|2020-CX0652.4	Asia / China / Yunnan	2020-06-	A/Yunnan-	WHO Chinese National Influenza Center	WHO Chinese National Influenza Center	Xin li,Li Xiyan, Dayan Wang, Zeng Xiaoxu
**EPI_ISL_527221**	EPI1800576|78-	Europe / Hungary	2020-03-	A/Hungary/108/2020	Central Veterinary Institute Budapest	Crick Worldwide Influenza Centre	NA
**EPI_ISL_527231**	EPI1800595|91-	Europe / Ireland	2020-03-	A/Ireland/23296/2020	UCD National Virus Reference Laboratory	Crick Worldwide Influenza Centre	NA
**EPI_ISL_527239**	EPI1800611|92-	Europe / Russian Federation	2020-03-	A/Moscow/119/2020	Ivanovsky Research Institute of Virology RAMS	Crick Worldwide Influenza Centre	NA
**EPI_ISL_528732**	EPI1800922|A/Saint-	Europe / Russian Federation /	2020-03-	A/Saint-Petersburg/RII-	Research Institute of Influenza, Ministry of Healthcare of the Russian	WHO National Influenza Centre Russian	NA
**EPI_ISL_528754**	EPI1800961|N10039976.4	Oceania / New Zealand /	2020-04-	A/Christchurch/502/20	Canterbury Health Services	WHO Collaborating Centre for Reference	Deng,Y-M; Iannello,P; Lau,H; Todd,A;
**EPI_ISL_528755**	EPI1800964|N10039977.4	Asia / Malaysia / Federal	2020-01-	A/Malaysia/12156400/	University Malaya	WHO Collaborating Centre for Reference	Deng,Y-M; Iannello,P; Lau,H; Todd,A;
**EPI_ISL_528757**	EPI1800970|N10039979.4	Oceania / New Zealand /	2020-03-	A/Christchurch/513/20	Canterbury Health Services	WHO Collaborating Centre for Reference	Deng,Y-M; Iannello,P; Lau,H; Todd,A;
**EPI_ISL_528758**	EPI1800973|N10040137.4	Oceania / Australia / Northern	2020-03-	A/Darwin/121/2020	Influenza Surveillance Centre for Disease Control	WHO Collaborating Centre for Reference	Deng,Y-M; Iannello,P; Lau,H; Todd,A;
**EPI_ISL_528870**	EPI1801189|3000824595_N8	Africa / Togo	2020-01-	A/Togo/129/2020	Institute National D'Hygiene	Centers for Disease Control and Prevention	NA
**EPI_ISL_528872**	EPI1801205|3000824613_N8	Africa / Togo	2020-03-	A/Togo/556/2020	Institute National D'Hygiene	Centers for Disease Control and Prevention	NA
**EPI_ISL_528873**	EPI1801213|3000824585_N8	North America / Honduras	2020-03-	A/Honduras/6723/202	Laboratorio Nacional de Virologia	Centers for Disease Control and Prevention	NA
**EPI_ISL_528874**	EPI1801221|3000824581_N8	North America / Honduras	2020-03-	A/Honduras/6580/202	Laboratorio Nacional de Virologia	Centers for Disease Control and Prevention	NA
**EPI_ISL_528875**	EPI1801229|3000824611_N8	Africa / Togo	2020-03-	A/Togo/434/2020	Institute National D'Hygiene	Centers for Disease Control and Prevention	NA
**EPI_ISL_528876**	EPI1801237|3000824609_N8	Africa / Togo	2020-03-	A/Togo/431/2020	Institute National D'Hygiene	Centers for Disease Control and Prevention	NA
**EPI_ISL_528883**	EPI1801284|3000824594_N8	Africa / Togo	2020-01-	A/Togo/127/2020	Institute National D'Hygiene	Centers for Disease Control and Prevention	NA
**EPI_ISL_528889**	EPI1801326|3000824612_N8	Africa / Togo	2020-03-	A/Togo/509/2020	Institute National D'Hygiene	Centers for Disease Control and Prevention	NA
**EPI_ISL_528890**	EPI1801334|3000824608_N8	Africa / Togo	2020-03-	A/Togo/459/2020	Institute National D'Hygiene	Centers for Disease Control and Prevention	NA
**EPI_ISL_528892**	EPI1801350|3000824605_N8	Africa / Togo	2020-02-	A/Togo/438/2020	Institute National D'Hygiene	Centers for Disease Control and Prevention	NA
**EPI_ISL_528893**	EPI1801358|3000824607_N8	Africa / Togo	2020-03-	A/Togo/455/2020	Institute National D'Hygiene	Centers for Disease Control and Prevention	NA
**EPI_ISL_529037**	EPI1801867|50-	Europe / Spain	2020-03-	A/Aragon/13140/2020	Instituto de Salud Carlos III	Crick Worldwide Influenza Centre	NA
**EPI_ISL_529039**	EPI1801871|58-	Asia / Lebanon	2020-03-	A/Beirut/421/2020	Rafic Hariri University Hospital 4th floor DNA/Research Lab	Crick Worldwide Influenza Centre	NA
**EPI_ISL_529043**	EPI1801879|57-	Europe / Iceland	2020-03-	A/Iceland/23/2020	Landspitali - University Hospital	Crick Worldwide Influenza Centre	NA
**EPI_ISL_529047**	EPI1801887|47-	Europe / Moldova, Republic of	2020-03-	A/Moldova/416.1/2020	National Centre for Preventive Medicine	Crick Worldwide Influenza Centre	NA
**EPI_ISL_529129**	EPI1802083|N10040328.4	Oceania / Australia /	2020-02-	A/Brisbane/16/2020	Queensland Health Forensic and Scientific Services	WHO Collaborating Centre for Reference	Deng,Y-M; Iannello,P; Lau,H; Todd,A;
**EPI_ISL_534009**	EPI1803979|A/Baramulla/UG_	Asia / India	2020-02-	A/Baramulla/UG_76/2	Sher-i-Kashmir Institute of Medical Sciences	CNR Virus des Infections Respiratoires -	NA
**EPI_ISL_534308**	EPI1804371|VIR20080709_H	Asia / Cambodia / Takeo	2020-08-	A/Cambodia/2008070	National Public Health Laboratory	Institut Pasteur du Cambodia	Savuth, C; Darapheak, C; Yann,S; Karlsson, E.
**EPI_ISL_534310**	EPI1804375|VIR20080717_H	Asia / Cambodia / Takeo	2020-08-	A/Cambodia/2008071	National Public Health Laboratory	Institut Pasteur du Cambodia	Savuth, C; Darapheak, C; Yann,S; Karlsson, E.
**EPI_ISL_534350**	EPI1804391|3000824616_N8	Africa / Togo	2020-03-	A/Togo/565/2020	Institute National D'Hygiene	Centers for Disease Control and Prevention	NA

**EPI_ISL_534351**	EPI1804399|3000824625_N8	Africa / Togo	2020-03-	A/Togo/545/2020	Institute National D'Hygiene	Centers for Disease Control and Prevention	NA
**EPI_ISL_534352**	EPI1804407|3000824624_N8	Africa / Togo	2020-03-	A/Togo/539/2020	Institute National D'Hygiene	Centers for Disease Control and Prevention	NA
**EPI_ISL_534353**	EPI1804414|3000824622_N8	Africa / Togo	2020-03-	A/Togo/534/2020	Institute National D'Hygiene	Centers for Disease Control and Prevention	NA
**EPI_ISL_534354**	EPI1804422|3000824621_N8	Africa / Togo	2020-03-	A/Togo/530/2020	Institute National D'Hygiene	Centers for Disease Control and Prevention	NA
**EPI_ISL_534355**	EPI1804430|3000824623_N8	Africa / Togo	2020-03-	A/Togo/538/2020	Institute National D'Hygiene	Centers for Disease Control and Prevention	NA
**EPI_ISL_534356**	EPI1804438|3000824619_N8	Africa / Togo	2020-03-	A/Togo/575/2020	Institute National D'Hygiene	Centers for Disease Control and Prevention	NA
**EPI_ISL_534357**	EPI1804445|3000824620_N8	Africa / Togo	2020-03-	A/Togo/529/2020	Institute National D'Hygiene	Centers for Disease Control and Prevention	NA
**EPI_ISL_534358**	EPI1804453|3000824615_N8	Africa / Togo	2020-03-	A/Togo/526/2020	Institute National D'Hygiene	Centers for Disease Control and Prevention	NA
**EPI_ISL_535490**	EPI1804563|A/HIROSHIMA-	Asia / Japan	2019-10-	A/HIROSHIMA-	Hiroshima City Institute of Public Health	National Institute of Infectious Diseases	Takashita,Emi;Fujisaki,Seiichiro;Shirakura,Masa
**EPI_ISL_536566**	EPI1804669|e0911253-H3	Asia / Cambodia / Siem Reap	2020-09-	A/Cambodia/e091125	National Public Health Laboratory	Institut Pasteur du Cambodia	Savuth, C; Darapheak,C; Y,P; Yann,S;
**EPI_ISL_536567**	EPI1804670|e0908364-H3	Asia / Cambodia / Phnom Penh	2020-07-	A/Cambodia/e090836	National Public Health Laboratory	Institut Pasteur du Cambodia	Savuth, C; Darapheak,C; Y,P; Yann,S;
**EPI_ISL_536568**	EPI1804671|e0908371-H3	Asia / Cambodia / Khett Svay	2020-07-	A/Cambodia/e090837	National Public Health Laboratory	Institut Pasteur du Cambodia	Savuth, C; Darapheak,C; Y,P; Yann,S;
**EPI_ISL_536569**	EPI1804672|e0908377-H3	Asia / Cambodia / Phnom Penh	2020-07-	A/Cambodia/e090837	National Public Health Laboratory	Institut Pasteur du Cambodia	Savuth, C; Darapheak,C; Y,P; Yann,S;
**EPI_ISL_536570**	EPI1804673|70632-H3	Asia / Cambodia / Koh Kong	2020-09-	A/Cambodia/70632/20	National Public Health Laboratory	Institut Pasteur du Cambodia	Savuth, C; Darapheak,C; Y,P; Yann,S;
**EPI_ISL_572186**	EPI1805148|A/KYOTO-	Asia / Japan	2019-09-	A/KYOTO-C/9/2019	Kyoto City Institute of Health and Environmental Sciences	National Institute of Infectious Diseases	Takashita,Emi;Fujisaki,Seiichiro;Shirakura,Masa
**EPI_ISL_572194**	EPI1805172|A/Myanmar/I128/	Asia / Myanmar	2020-03-	A/Myanmar/I128/2020	National Health Laboratory	National Institute of Infectious Diseases	Takashita,Emi;Fujisaki,Seiichiro;Shirakura,Masa
**EPI_ISL_577608**	EPI1805888|A/GUNMA/51/202	Asia / Japan	2020-03-	A/GUNMA/51/2020	Gunma Prefectural Institute of Public Health and Environmental	National Institute of Infectious Diseases	Takashita,Emi;Fujisaki,Seiichiro;Shirakura,Masa
**EPI_ISL_577614**	EPI1805926|A/SAITAMA/89/2	Asia / Japan	2020-02-	A/SAITAMA/89/2020	Saitama Institute of Public Health	National Institute of Infectious Diseases	Takashita,Emi;Fujisaki,Seiichiro;Shirakura,Masa
**EPI_ISL_577615**	EPI1805934|A/SAITAMA/92/2	Asia / Japan	2020-03-	A/SAITAMA/92/2020	Saitama Institute of Public Health	National Institute of Infectious Diseases	Takashita,Emi;Fujisaki,Seiichiro;Shirakura,Masa
**EPI_ISL_577616**	EPI1805942|A/SAITAMA/95/2	Asia / Japan	2020-03-	A/SAITAMA/95/2020	Saitama Institute of Public Health	National Institute of Infectious Diseases	Takashita,Emi;Fujisaki,Seiichiro;Shirakura,Masa
**EPI_ISL_577618**	EPI1805953|A/Nepal/20FL024	Asia / Nepal	2020-02-	A/Nepal/20FL0243/20	National Public Health Laboratory	National Institute of Infectious Diseases	Takashita,Emi;Fujisaki,Seiichiro;Shirakura,Masa
**EPI_ISL_577619**	EPI1805961|A/Nepal/20FL068	Asia / Nepal	2020-03-	A/Nepal/20FL0682/20	National Public Health Laboratory	National Institute of Infectious Diseases	Takashita,Emi;Fujisaki,Seiichiro;Shirakura,Masa
**EPI_ISL_577620**	EPI1805964|A/Nepal/20FL077	Asia / Nepal	2020-03-	A/Nepal/20FL0776/20	National Public Health Laboratory	National Institute of Infectious Diseases	Takashita,Emi;Fujisaki,Seiichiro;Shirakura,Masa
**EPI_ISL_577621**	EPI1805967|A/Nepal/20FL085	Asia / Nepal	2020-03-	A/Nepal/20FL0852/20	National Public Health Laboratory	National Institute of Infectious Diseases	Takashita,Emi;Fujisaki,Seiichiro;Shirakura,Masa
**EPI_ISL_577622**	EPI1805975|A/Nepal/20FL100	Asia / Nepal	2020-03-	A/Nepal/20FL1005/20	National Public Health Laboratory	National Institute of Infectious Diseases	Takashita,Emi;Fujisaki,Seiichiro;Shirakura,Masa
**EPI_ISL_583902**	EPI1806123|S10040730.4	Asia / Indonesia	2020-03-	A/Indonesia/NIHRDBJ	Ministry of Health, NIHRD	WHO Collaborating Centre for Reference	Siswanto,S; Setiawaty,V; Deng, Y-M;
**EPI_ISL_583999**	EPI1806183|3001288014_N8	South America / Brazil	2019-07-	A/Minas	Instituto Oswaldo Cruz FIOCRUZ - Laboratory of Respiratory Viruses	Centers for Disease Control and Prevention	NA
**EPI_ISL_584000**	EPI1806191|3001288017_N8	South America / Brazil	2019-08-	A/Minas	Instituto Oswaldo Cruz FIOCRUZ - Laboratory of Respiratory Viruses	Centers for Disease Control and Prevention	NA
**EPI_ISL_584001**	EPI1806197|3001288053_N8	North America / Guatemala	2020-03-	A/Guatemala/138/202	Laboratorio Nacional De Salud Guatemala	Centers for Disease Control and Prevention	NA
**EPI_ISL_584002**	EPI1806205|3001288128_N8	Asia / Bangladesh	2020-01-	A/Bangladesh/3003/2	icddr,b International Centre for Diarrhoeal Disease Research,	Centers for Disease Control and Prevention	NA
**EPI_ISL_584003**	EPI1806213|3001288110_N8	Asia / Bangladesh	2020-08-	A/Bangladesh/10006/	icddr,b International Centre for Diarrhoeal Disease Research,	Centers for Disease Control and Prevention	NA
**EPI_ISL_584004**	EPI1806221|3001288103_N8	Asia / Bangladesh	2020-08-	A/Bangladesh/8001/2	icddr,b International Centre for Diarrhoeal Disease Research,	Centers for Disease Control and Prevention	NA
**EPI_ISL_584005**	EPI1806229|3001288108_N8	Asia / Bangladesh	2020-08-	A/Bangladesh/10009/	icddr,b International Centre for Diarrhoeal Disease Research,	Centers for Disease Control and Prevention	NA
**EPI_ISL_584006**	EPI1806237|3001288035_N8	South America / Brazil	2020-02-	A/Minas	Instituto Oswaldo Cruz FIOCRUZ - Laboratory of Respiratory Viruses	Centers for Disease Control and Prevention	NA
**EPI_ISL_584007**	EPI1806245|3001288034_N8	South America / Brazil	2020-03-	A/Minas	Instituto Oswaldo Cruz FIOCRUZ - Laboratory of Respiratory Viruses	Centers for Disease Control and Prevention	NA
**EPI_ISL_584008**	EPI1806253|3001288026_N8	South America / Brazil	2020-01-	A/Minas	Instituto Oswaldo Cruz FIOCRUZ - Laboratory of Respiratory Viruses	Centers for Disease Control and Prevention	NA
**EPI_ISL_584009**	EPI1806258|3001288044_N8	North America / Guatemala	2020-03-	A/Guatemala/07/2020	Laboratorio Nacional De Salud Guatemala	Centers for Disease Control and Prevention	NA
**EPI_ISL_584010**	EPI1806266|3001288025_N8	South America / Brazil	2019-12-	A/Minas	Instituto Oswaldo Cruz FIOCRUZ - Laboratory of Respiratory Viruses	Centers for Disease Control and Prevention	NA
**EPI_ISL_593785**	EPI1806955|A/Nepal/20FL085	Asia / Nepal	2020-03-	A/Nepal/20FL0850/20	National Public Health Laboratory	National Institute of Infectious Diseases	Takashita,Emi;Fujisaki,Seiichiro;Shirakura,Masa
**EPI_ISL_613415**	EPI1807503|3000824646_N8	South America / Argentina	2020-03-	A/Argentina/14818/20	Intituto Nacional de Epidemiologia	Centers for Disease Control and Prevention	NA
**EPI_ISL_614328**	EPI1811530|2019-CX2210.4	Asia / China / Guangxi	2019-06-	A/Guangxi-	WHO Chinese National Influenza Center	WHO Chinese National Influenza Center	Lixiyan, Huangweijuan, Dayan Wang, Liu Jia
**EPI_ISL_614333**	EPI1811545|2019-CX2205.4	Asia / China / Guangxi	2019-06-	A/Guangxi-	WHO Chinese National Influenza Center	WHO Chinese National Influenza Center	Lixiyan, Huangweijuan, Dayan Wang, Liu Jia
**EPI_ISL_614338**	EPI1808406|S10040832.4	Asia / Indonesia	2020-03-	A/Indonesia/NIHRDS	Ministry of Health, NIHRD	WHO Collaborating Centre for Reference	Siswanto,S; Setiawaty,V; Deng, Y-M;
**EPI_ISL_614339**	EPI1808412|S10040833.4	Asia / Indonesia	2020-03-	A/Indonesia/NIHRDDP	Ministry of Health, NIHRD	WHO Collaborating Centre for Reference	Siswanto,S; Setiawaty,V; Deng, Y-M;
**EPI_ISL_614340**	EPI1808418|S10040834.4	Asia / Indonesia	2020-03-	A/Indonesia/NIHRDS	Ministry of Health, NIHRD	WHO Collaborating Centre for Reference	Siswanto,S; Setiawaty,V; Deng, Y-M;
**EPI_ISL_614341**	EPI1808423|S10040835.4	Asia / Indonesia	2020-03-	A/Indonesia/NIHRDLP	Ministry of Health, NIHRD	WHO Collaborating Centre for Reference	Siswanto,S; Setiawaty,V; Deng, Y-M;
**EPI_ISL_614342**	EPI1808430|S10040836.4	Asia / Indonesia	2020-03-	A/Indonesia/NIHRDBD	Ministry of Health, NIHRD	WHO Collaborating Centre for Reference	Siswanto,S; Setiawaty,V; Deng, Y-M;
**EPI_ISL_614343**	EPI1808435|S10040837.4	Asia / Indonesia	2020-03-	A/Indonesia/NIHRDS	Ministry of Health, NIHRD	WHO Collaborating Centre for Reference	Siswanto,S; Setiawaty,V; Deng, Y-M;
**EPI_ISL_628766**	EPI1811745|3001288303_N8	Asia / Bhutan	2020-02-	A/Bhutan/1129/2020	Royal Centre for Disease Control	Centers for Disease Control and Prevention	NA
**EPI_ISL_628767**	EPI1811752|3001288302_N8	Asia / Bhutan	2020-02-	A/Bhutan/1123/2020	Royal Centre for Disease Control	Centers for Disease Control and Prevention	NA
**EPI_ISL_628768**	EPI1811760|3001288300_N8	Asia / Bhutan	2020-02-	A/Bhutan/1104/2020	Royal Centre for Disease Control	Centers for Disease Control and Prevention	NA
**EPI_ISL_628769**	EPI1811768|3001288281_N8	South America / Ecuador	2019-12-	A/Ecuador/1494/2019	INSPI	Centers for Disease Control and Prevention	NA
**EPI_ISL_628770**	EPI1811776|3001288306_N8	Asia / Bhutan	2020-01-	A/Bhutan/769/2020	Royal Centre for Disease Control	Centers for Disease Control and Prevention	NA
**EPI_ISL_628771**	EPI1811784|3001288307_N8	Asia / Bhutan	2020-02-	A/Bhutan/770/2020	Royal Centre for Disease Control	Centers for Disease Control and Prevention	NA
**EPI_ISL_628772**	EPI1811792|3001288311_N8	Asia / Bhutan	2020-03-	A/Bhutan/683/2020	Royal Centre for Disease Control	Centers for Disease Control and Prevention	NA
**EPI_ISL_628773**	EPI1811800|3001288310_N8	Asia / Bhutan	2020-02-	A/Bhutan/665/2020	Royal Centre for Disease Control	Centers for Disease Control and Prevention	NA
**EPI_ISL_628774**	EPI1811808|3001288294_N8	Asia / Bhutan	2020-01-	A/Bhutan/390/2020	Royal Centre for Disease Control	Centers for Disease Control and Prevention	NA
**EPI_ISL_628775**	EPI1811816|3001288298_N8	Asia / Bhutan	2020-03-	A/Bhutan/978/2020	Royal Centre for Disease Control	Centers for Disease Control and Prevention	NA
**EPI_ISL_628776**	EPI1811824|3001288297_N8	Asia / Bhutan	2020-02-	A/Bhutan/954/2020	Royal Centre for Disease Control	Centers for Disease Control and Prevention	NA
**EPI_ISL_628777**	EPI1811832|3001288309_N8	Asia / Bhutan	2020-02-	A/Bhutan/663/2020	Royal Centre for Disease Control	Centers for Disease Control and Prevention	NA
**EPI_ISL_644649**	EPI1813629|N10040723.4	Asia / Thailand / Nonthaburi	2020-01-	A/Nonthaburi/3/2020	WHO National Influenza Centre, National Institute of Medical Research	WHO Collaborating Centre for Reference	Deng,Y-M; Iannello,P; Lau,H; Todd,A;
**EPI_ISL_644651**	EPI1813645|N10040725.4	Asia / Thailand /	2020-01-	A/Nakhonphanom/25/	WHO National Influenza Centre, National Institute of Medical Research	WHO Collaborating Centre for Reference	Deng,Y-M; Iannello,P; Lau,H; Todd,A;
**EPI_ISL_644652**	EPI1813653|N10040726.4	Asia / Indonesia	2020-03-	A/Indonesia/NIHRDDP	Ministry of Health, NIHRD	WHO Collaborating Centre for Reference	Siswanto,S; Setiawaty,V; Deng, Y-M;
**EPI_ISL_644654**	EPI1813669|N10040728.4	Asia / Thailand / Chiang Rai	2020-02-	A/Chiang Rai/48/2020	WHO National Influenza Centre, National Institute of Medical Research	WHO Collaborating Centre for Reference	Deng,Y-M; Iannello,P; Lau,H; Todd,A;
**EPI_ISL_645155**	EPI1813793|HA_20-	Europe / Germany	2020-03-	A/Berlin/45/2020	Robert Koch-Institute Nationales Referenzzentrum für Influenza	Robert Koch Institute Nationales	NA
**EPI_ISL_645156**	EPI1813794|HA_20-	Europe / Germany	2020-03-	A/Niedersachsen/138/	Robert Koch-Institute Nationales Referenzzentrum für Influenza	Robert Koch Institute Nationales	NA
**EPI_ISL_645157**	EPI1813795|HA_20-	Europe / Germany	2020-03-	A/Sachsen/72/2020	Robert Koch-Institute Nationales Referenzzentrum für Influenza	Robert Koch Institute Nationales	NA
**EPI_ISL_645210**	EPI1813808|HA_20-	Europe / Germany	2020-03-	A/Brandenburg/13/202	Robert Koch-Institute Nationales Referenzzentrum für Influenza	Robert Koch Institute Nationales	NA

**EPI_ISL_645211**	EPI1813817|HA_20-	Europe / Germany	2020-03-	A/Berlin/64/2020	Robert Koch-Institute Nationales Referenzzentrum für Influenza	Robert Koch Institute Nationales	NA
**EPI_ISL_654685**	EPI1813999|3001288367_N8	South America / Bolivia,	2020-03-	A/Bolivia/430/2020	Instituto Nacional de Laboratoriosde Salud (INLASA)	Centers for Disease Control and Prevention	NA
**EPI_ISL_654686**	EPI1814007|3001288368_N8	South America / Bolivia,	2020-03-	A/Bolivia/436/2020	Instituto Nacional de Laboratoriosde Salud (INLASA)	Centers for Disease Control and Prevention	NA
**EPI_ISL_654687**	EPI1814015|3001288327_N8	Asia / Bhutan	2020-01-	A/Bhutan/286/2020	Royal Centre for Disease Control	Centers for Disease Control and Prevention	NA
**EPI_ISL_654688**	EPI1814023|3001288328_N8	Asia / Bhutan	2020-02-	A/Bhutan/287/2020	Royal Centre for Disease Control	Centers for Disease Control and Prevention	NA
**EPI_ISL_660119**	EPI1814614|S10040994.4	Oceania / Timor-Leste	2020-08-	A/Timor-Leste/2/2020	Laboratório Nacional da Saúde	WHO Collaborating Centre for Reference	Da Costa,E; Tilman,A; Barreto,I; Freitas,L;
**EPI_ISL_660120**	EPI1814617|S10040995.4	Oceania / Timor-Leste	2020-07-	A/Timor-	Laboratório Nacional da Saúde	WHO Collaborating Centre for Reference	Da Costa,E; Tilman,A; Barreto,I; Freitas,L;
**EPI_ISL_671412**	EPI1814758|A/Lyon/1773/202	Europe / France	2020-03-	A/Lyon/1773/2020	CNR Virus des Infections Respiratoires - France SUD	CNR Virus des Infections Respiratoires -	NA
**EPI_ISL_684048**	EPI1815212|A/YOKOHAMA/2	Asia / Japan	2019-11-	A/YOKOHAMA/224/20	Yokohama City Institute of Public Health.	National Institute of Infectious Diseases	Takashita,Emi;Fujisaki,Seiichiro;Shirakura,Masa
**EPI_ISL_684050**	EPI1815228|A/YAMANASHI/1	Asia / Japan	2019-12-	A/YAMANASHI/19395/	Yamanashi Institute for Public Health	National Institute of Infectious Diseases	Takashita,Emi;Fujisaki,Seiichiro;Shirakura,Masa
**EPI_ISL_684051**	EPI1815236|A/SAPPORO/61/	Asia / Japan	2019-11-	A/SAPPORO/61/2019	Sapporo City Institute of Public Health	National Institute of Infectious Diseases	Takashita,Emi;Fujisaki,Seiichiro;Shirakura,Masa
**EPI_ISL_684054**	EPI1815260|A/GUNMA/47/202	Asia / Japan	2020-02-	A/GUNMA/47/2020	Gunma Prefectural Institute of Public Health and Environmental	National Institute of Infectious Diseases	Takashita,Emi;Fujisaki,Seiichiro;Shirakura,Masa
**EPI_ISL_707784**	EPI1836966|A/Grenoble/2789/	Europe / France	2020-10-	A/Grenoble/2789/202	CNR Virus des Infections Respiratoires - France SUD	CNR Virus des Infections Respiratoires -	NA
**EPI_ISL_710454**	EPI1837690|N10041132.4	Asia / Cambodia / Battambang	2020-09-	A/Cambodia/nC_7183	Institute Pasteur du Cambodia	WHO Collaborating Centre for Reference	Deng,Y-M; Iannello,P; Lau,H; Spirason,N;
**EPI_ISL_710455**	EPI1837693|N10041133.4	Asia / Cambodia / Battambang	2020-09-	A/Cambodia/nC_7184	Institute Pasteur du Cambodia	WHO Collaborating Centre for Reference	Deng,Y-M; Iannello,P; Lau,H; Spirason,N;
**EPI_ISL_710456**	EPI1837696|N10041134.4	Asia / Cambodia / Battambang	2020-09-	A/Cambodia/nC_7184	Institute Pasteur du Cambodia	WHO Collaborating Centre for Reference	Deng,Y-M; Iannello,P; Lau,H; Spirason,N;
**EPI_ISL_710457**	EPI1837699|N10041135.4	Asia / Cambodia / Ratanak Kiri	2020-09-	A/Cambodia/nC_7330	Institute Pasteur du Cambodia	WHO Collaborating Centre for Reference	Deng,Y-M; Iannello,P; Lau,H; Spirason,N;
**EPI_ISL_710458**	EPI1837702|N10041136.4	Asia / Cambodia / Ratanak Kiri	2020-09-	A/Cambodia/nC_7330	Institute Pasteur du Cambodia	WHO Collaborating Centre for Reference	Deng,Y-M; Iannello,P; Lau,H; Spirason,N;
**EPI_ISL_710459**	EPI1837705|N10041137.4	Asia / Cambodia / Kampong	2020-09-	A/Cambodia/nC_7739	Institute Pasteur du Cambodia	WHO Collaborating Centre for Reference	Deng,Y-M; Iannello,P; Lau,H; Spirason,N;
**EPI_ISL_710460**	EPI1837708|N10041140.4	Asia / Cambodia / Khett Svay	2020-09-	A/Cambodia/nC_8062	Institute Pasteur du Cambodia	WHO Collaborating Centre for Reference	Deng,Y-M; Iannello,P; Lau,H; Spirason,N;
**EPI_ISL_710461**	EPI1837711|N10041146.4	Asia / Cambodia / Battambang	2020-11-	A/Cambodia/nC_1061	Institute Pasteur du Cambodia	WHO Collaborating Centre for Reference	Deng,Y-M; Iannello,P; Lau,H; Spirason,N;
**EPI_ISL_710462**	EPI1837714|N10041147.4	Asia / Cambodia / Takeo	2020-09-	A/Cambodia/20080-	Institute Pasteur du Cambodia	WHO Collaborating Centre for Reference	Deng,Y-M; Iannello,P; Lau,H; Spirason,N;
**EPI_ISL_710463**	EPI1837717|N10041148.4	Asia / Cambodia / Khett Preah	2020-08-	A/Cambodia/20080-	Institute Pasteur du Cambodia	WHO Collaborating Centre for Reference	Deng,Y-M; Iannello,P; Lau,H; Spirason,N;
**EPI_ISL_710464**	EPI1837720|N10041150.4	Asia / Cambodia / Khett Preah	2020-08-	A/Cambodia/20080-	Institute Pasteur du Cambodia	WHO Collaborating Centre for Reference	Deng,Y-M; Iannello,P; Lau,H; Spirason,N;
**EPI_ISL_710465**	EPI1837723|N10041151.4	Asia / Cambodia / Khett Preah	2020-08-	A/Cambodia/20080-	Institute Pasteur du Cambodia	WHO Collaborating Centre for Reference	Deng,Y-M; Iannello,P; Lau,H; Spirason,N;
**EPI_ISL_710466**	EPI1837726|N10041152.4	Asia / Cambodia / Khett Preah	2020-08-	A/Cambodia/20080-	Institute Pasteur du Cambodia	WHO Collaborating Centre for Reference	Deng,Y-M; Iannello,P; Lau,H; Spirason,N;
**EPI_ISL_710467**	EPI1837729|N10041153.4	Asia / Cambodia / Tbong	2020-08-	A/Cambodia/VIR2023	Institute Pasteur du Cambodia	WHO Collaborating Centre for Reference	Deng,Y-M; Iannello,P; Lau,H; Spirason,N;
**EPI_ISL_710468**	EPI1837732|N10041154.4	Asia / Cambodia / Tbong	2020-08-	A/Cambodia/VIR2023	Institute Pasteur du Cambodia	WHO Collaborating Centre for Reference	Deng,Y-M; Iannello,P; Lau,H; Spirason,N;
**EPI_ISL_710469**	EPI1837735|N10041155.4	Asia / Cambodia / Tbong	2020-08-	A/Cambodia/VIR2023	Institute Pasteur du Cambodia	WHO Collaborating Centre for Reference	Deng,Y-M; Iannello,P; Lau,H; Spirason,N;
**EPI_ISL_710470**	EPI1837738|N10041156.4	Asia / Cambodia / Battambang	2020-08-	A/Cambodia/VIR	Institute Pasteur du Cambodia	WHO Collaborating Centre for Reference	Deng,Y-M; Iannello,P; Lau,H; Spirason,N;
**EPI_ISL_710471**	EPI1837741|N10041157.4	Asia / Cambodia / Phnom Penh	2020-08-	A/Cambodia/VIR	Institute Pasteur du Cambodia	WHO Collaborating Centre for Reference	Deng,Y-M; Iannello,P; Lau,H; Spirason,N;
**EPI_ISL_710472**	EPI1837744|N10041158.4	Asia / Cambodia	2020-08-	A/Cambodia/VIR	Institute Pasteur du Cambodia	WHO Collaborating Centre for Reference	Deng,Y-M; Iannello,P; Lau,H; Spirason,N;
**EPI_ISL_710473**	EPI1837747|N10041159.4	Asia / Cambodia / Phnom Penh	2020-08-	A/Cambodia/VIR	Institute Pasteur du Cambodia	WHO Collaborating Centre for Reference	Deng,Y-M; Iannello,P; Lau,H; Spirason,N;
**EPI_ISL_710474**	EPI1837750|N10041160.4	Asia / Cambodia / Phnom Penh	2020-08-	A/Cambodia/VIR	Institute Pasteur du Cambodia	WHO Collaborating Centre for Reference	Deng,Y-M; Iannello,P; Lau,H; Spirason,N;
**EPI_ISL_710475**	EPI1837753|N10041161.4	Asia / Cambodia / Battambang	2020-07-	A/Cambodia/e082636	Institute Pasteur du Cambodia	WHO Collaborating Centre for Reference	Deng,Y-M; Iannello,P; Lau,H; Spirason,N;
**EPI_ISL_710478**	EPI1837762|N10041167.4	Asia / Cambodia / Phnom Penh	2020-08-	A/Cambodia/e090838	Institute Pasteur du Cambodia	WHO Collaborating Centre for Reference	Deng,Y-M; Iannello,P; Lau,H; Spirason,N;
**EPI_ISL_710480**	EPI1837765|N10041162.4	Asia / Cambodia / Battambang	2020-07-	A/Cambodia/e082636	Institute Pasteur du Cambodia	WHO Collaborating Centre for Reference	Deng,Y-M; Iannello,P; Lau,H; Spirason,N;
**EPI_ISL_710481**	EPI1837768|N10041163.4	Asia / Cambodia / Battambang	2020-07-	A/Cambodia/e082636	Institute Pasteur du Cambodia	WHO Collaborating Centre for Reference	Deng,Y-M; Iannello,P; Lau,H; Spirason,N;
**EPI_ISL_710482**	EPI1837771|N10041164.4	Asia / Cambodia / Battambang	2020-07-	A/Cambodia/e082636	Institute Pasteur du Cambodia	WHO Collaborating Centre for Reference	Deng,Y-M; Iannello,P; Lau,H; Spirason,N;
**EPI_ISL_710500**	EPI1837825|A/AICHI/358/2019	Asia / Japan	2019-11-	A/AICHI/358/2019	Aichi Prefectural Institute of Public Health	National Institute of Infectious Diseases	Takashita,Emi;Fujisaki,Seiichiro;Shirakura,Masa
**EPI_ISL_710501**	EPI1837833|A/SAITAMA/88/2	Asia / Japan	2020-02-	A/SAITAMA/88/2020	Saitama Institute of Public Health	National Institute of Infectious Diseases	Takashita,Emi;Fujisaki,Seiichiro;Shirakura,Masa
**EPI_ISL_710502**	EPI1837841|A/SAITAMA/94/2	Asia / Japan	2020-03-	A/SAITAMA/94/2020	Saitama Institute of Public Health	National Institute of Infectious Diseases	Takashita,Emi;Fujisaki,Seiichiro;Shirakura,Masa
**EPI_ISL_717646**	EPI1838081|3030724497_ZZ	North America / United States /	2020-10-	A/California/55/2020	California Department of Health Services	Centers for Disease Control and Prevention	NA
**EPI_ISL_717648**	EPI1838097|3001288248_N8	Asia / Lao, People's	2020-03-	A/Laos/892/2020	National Center for Laboratory and Epidemiology	Centers for Disease Control and Prevention	NA
**EPI_ISL_717651**	EPI1838121|3001288246_N8	Asia / Lao, People's	2020-03-	A/Laos/735/2020	National Center for Laboratory and Epidemiology	Centers for Disease Control and Prevention	NA
**EPI_ISL_717665**	EPI1838233|3001288247_N8	Asia / Lao, People's	2020-03-	A/Laos/939/2020	National Center for Laboratory and Epidemiology	Centers for Disease Control and Prevention	NA
**EPI_ISL_717667**	EPI1838249|3001288407_N8	Africa / Mali	2020-03-	A/Mali/0051/2020	NIC Lab CVD-MALI	Centers for Disease Control and Prevention	NA
**EPI_ISL_717669**	EPI1838265|3001288405_N8	Africa / Mali	2020-03-	A/Mali/20064/2020	NIC Lab CVD-MALI	Centers for Disease Control and Prevention	NA
**EPI_ISL_717671**	EPI1838281|3001288400_N8	Africa / Mali	2020-03-	A/Mali/20076/2020	NIC Lab CVD-MALI	Centers for Disease Control and Prevention	NA
**EPI_ISL_717672**	EPI1838289|3001288406_N8	Africa / Mali	2020-03-	A/Mali/0048/2020	NIC Lab CVD-MALI	Centers for Disease Control and Prevention	NA
**EPI_ISL_717673**	EPI1838296|3001288449_N8	Asia / Bangladesh	2020-10-	A/Bangladesh/101000	icddr,b International Centre for Diarrhoeal Disease Research,	Centers for Disease Control and Prevention	NA
**EPI_ISL_717674**	EPI1838303|3001288456_N8	Asia / Bangladesh	2020-10-	A/Bangladesh/4005/2	icddr,b International Centre for Diarrhoeal Disease Research,	Centers for Disease Control and Prevention	NA
**EPI_ISL_717675**	EPI1838311|3001288448_N8	Asia / Bangladesh	2020-10-	A/Bangladesh/100400	icddr,b International Centre for Diarrhoeal Disease Research,	Centers for Disease Control and Prevention	NA
**EPI_ISL_717676**	EPI1838318|3001288446_N8	Asia / Bangladesh	2020-09-	A/Bangladesh/3016/2	icddr,b International Centre for Diarrhoeal Disease Research,	Centers for Disease Control and Prevention	NA
**EPI_ISL_717677**	EPI1838326|3001288443_N8	Asia / Bangladesh	2020-09-	A/Bangladesh/1003/2	icddr,b International Centre for Diarrhoeal Disease Research,	Centers for Disease Control and Prevention	NA
**EPI_ISL_717678**	EPI1838334|3001288439_N8	Asia / Bangladesh	2020-09-	A/Bangladesh/2007/2	icddr,b International Centre for Diarrhoeal Disease Research,	Centers for Disease Control and Prevention	NA
**EPI_ISL_717679**	EPI1838341|3001288438_N8	Asia / Bangladesh	2020-09-	A/Bangladesh/1001/2	icddr,b International Centre for Diarrhoeal Disease Research,	Centers for Disease Control and Prevention	NA
**EPI_ISL_717680**	EPI1838349|3001288445_N8	Asia / Bangladesh	2020-09-	A/Bangladesh/3011/2	icddr,b International Centre for Diarrhoeal Disease Research,	Centers for Disease Control and Prevention	NA
**EPI_ISL_717681**	EPI1838357|3001288440_N8	Asia / Bangladesh	2020-09-	A/Bangladesh/3005/2	icddr,b International Centre for Diarrhoeal Disease Research,	Centers for Disease Control and Prevention	NA
**EPI_ISL_717682**	EPI1838362|3001288453_N8	Asia / Bangladesh	2020-10-	A/Bangladesh/2002/2	icddr,b International Centre for Diarrhoeal Disease Research,	Centers for Disease Control and Prevention	NA
**EPI_ISL_717683**	EPI1838369|3001288450_N8	Asia / Bangladesh	2020-10-	A/Bangladesh/101000	icddr,b International Centre for Diarrhoeal Disease Research,	Centers for Disease Control and Prevention	NA
**EPI_ISL_717684**	EPI1838376|3001288451_N8	Asia / Bangladesh	2020-10-	A/Bangladesh/1002/2	icddr,b International Centre for Diarrhoeal Disease Research,	Centers for Disease Control and Prevention	NA
**EPI_ISL_717685**	EPI1838384|3001288454_N8	Asia / Bangladesh	2020-10-	A/Bangladesh/2004/2	icddr,b International Centre for Diarrhoeal Disease Research,	Centers for Disease Control and Prevention	NA
**EPI_ISL_717686**	EPI1838392|3001288444_N8	Asia / Bangladesh	2020-09-	A/Bangladesh/3014/2	icddr,b International Centre for Diarrhoeal Disease Research,	Centers for Disease Control and Prevention	NA
**EPI_ISL_717687**	EPI1838400|3001288442_N8	Asia / Bangladesh	2020-09-	A/Bangladesh/2019/2	icddr,b International Centre for Diarrhoeal Disease Research,	Centers for Disease Control and Prevention	NA
**EPI_ISL_717688**	EPI1838408|3001288437_N8	Asia / Bangladesh	2020-09-	A/Bangladesh/911009	icddr,b International Centre for Diarrhoeal Disease Research,	Centers for Disease Control and Prevention	NA
**EPI_ISL_717689**	EPI1838416|3001288447_N8	Asia / Bangladesh	2020-10-	A/Bangladesh/4002/2	icddr,b International Centre for Diarrhoeal Disease Research,	Centers for Disease Control and Prevention	NA
**EPI_ISL_717690**	EPI1838421|3001288455_N8	Asia / Bangladesh	2020-10-	A/Bangladesh/3004/2	icddr,b International Centre for Diarrhoeal Disease Research,	Centers for Disease Control and Prevention	NA

**EPI_ISL_718125**	EPI1838609|N10041168.4	Asia / Cambodia / Phnom Penh	2020-08-	A/Cambodia/e090838	Institute Pasteur du Cambodia	WHO Collaborating Centre for Reference	Deng,Y-M; Iannello,P; Lau,H; Spirason,N;
**EPI_ISL_718126**	EPI1838612|S10041126.4	Asia / Indonesia	2020-02-	A/Indonesia/NIHRDPA	Ministry of Health, NIHRD	WHO Collaborating Centre for Reference	Siswanto,S; Setiawaty,V; Deng, Y-M;
**EPI_ISL_718129**	EPI1838621|S10041186.4	Asia / Indonesia	2020-03-	A/Indonesia/NIHRDML	Ministry of Health, NIHRD	WHO Collaborating Centre for Reference	Siswanto,S; Setiawaty,V; Deng, Y-M;
**EPI_ISL_718130**	EPI1838624|S10041187.4	Asia / Sri Lanka /	2020-01-	A/Sri Lanka/65/2020	Medical Research Institute	WHO Collaborating Centre for Reference	Deng,Y-M; Iannello,P; Lau,H; Spirason,N;
**EPI_ISL_718131**	EPI1838627|S10041188.4	Asia / Sri Lanka / Southern	2020-01-	A/Sri Lanka/62/2020	Medical Research Institute	WHO Collaborating Centre for Reference	Deng,Y-M; Iannello,P; Lau,H; Spirason,N;
**EPI_ISL_732313**	EPI1838905|37-	Asia / Cambodia	2020-09-	A/Cambodia/71851/20	Institut Pasteur du Cambodia	Crick Worldwide Influenza Centre	NA
**EPI_ISL_732314**	EPI1838907|36-	Asia / Cambodia	2020-09-	A/Cambodia/73299/20	Institut Pasteur du Cambodia	Crick Worldwide Influenza Centre	NA
**EPI_ISL_732316**	EPI1838911|34-	Asia / Cambodia	2020-09-	A/Cambodia/925256/2	Institut Pasteur du Cambodia	Crick Worldwide Influenza Centre	NA
**EPI_ISL_732318**	EPI1838915|85-	Asia / Hong Kong (SAR)	2020-03-	A/Hong	Government Virus Unit	Crick Worldwide Influenza Centre	NA
**EPI_ISL_732319**	EPI1838917|95-	Asia / Cambodia	2020-09-	A/Cambodia/923251/2	Institut Pasteur du Cambodia	Crick Worldwide Influenza Centre	NA
**EPI_ISL_732320**	EPI1838919|94-	Africa / Tunisia	2020-03-	A/Tunisia/3085/2020	National Influenza Centre-Tunis, Unit Virology, Microbiology Laboratory,	Crick Worldwide Influenza Centre	NA
**EPI_ISL_732321**	EPI1838921|93-	Africa / Tunisia	2020-03-	A/Tunisia/3442/2020	National Influenza Centre-Tunis, Unit Virology, Microbiology Laboratory,	Crick Worldwide Influenza Centre	NA
**EPI_ISL_732322**	EPI1838922|92-	Africa / Tunisia	2020-03-	A/Tunisia/3666/2020	National Influenza Centre-Tunis, Unit Virology, Microbiology Laboratory,	Crick Worldwide Influenza Centre	NA
**EPI_ISL_732324**	EPI1838925|88-	Asia / Hong Kong (SAR)	2020-03-	A/Hong	Government Virus Unit	Crick Worldwide Influenza Centre	NA
**EPI_ISL_732326**	EPI1838929|86-	Asia / Hong Kong (SAR)	2020-03-	A/Hong	Government Virus Unit	Crick Worldwide Influenza Centre	NA
**EPI_ISL_732328**	EPI1838933|84-	Asia / Hong Kong (SAR)	2020-03-	A/Hong	Government Virus Unit	Crick Worldwide Influenza Centre	NA
**EPI_ISL_732329**	EPI1838935|83-	Asia / Hong Kong (SAR)	2020-03-	A/Hong	Government Virus Unit	Crick Worldwide Influenza Centre	NA
**EPI_ISL_732330**	EPI1838937|82-	Asia / Hong Kong (SAR)	2020-03-	A/Hong	Government Virus Unit	Crick Worldwide Influenza Centre	NA
**EPI_ISL_732331**	EPI1838938|81-	Asia / Hong Kong (SAR)	2020-03-	A/Hong	Government Virus Unit	Crick Worldwide Influenza Centre	NA
**EPI_ISL_732336**	EPI1838948|87-	Asia / Hong Kong (SAR)	2020-03-	A/Hong	Government Virus Unit	Crick Worldwide Influenza Centre	NA
**EPI_ISL_766791**	EPI1839538|3001288387_N8	Africa / Mali	2020-03-	A/Mali/20044/2020	NIC Lab CVD-MALI	Centers for Disease Control and Prevention	NA
**EPI_ISL_766793**	EPI1839554|3001288413_N8	Africa / Mali	2020-03-	A/Mali/20026/2020	NIC Lab CVD-MALI	Centers for Disease Control and Prevention	NA
**EPI_ISL_766794**	EPI1839562|3001288409_N8	Africa / Mali	2020-03-	A/Mali/20052/2020	NIC Lab CVD-MALI	Centers for Disease Control and Prevention	NA
**EPI_ISL_766795**	EPI1839570|3001288414_N8	Africa / Mali	2020-03-	A/Mali/0026/2020	NIC Lab CVD-MALI	Centers for Disease Control and Prevention	NA
**EPI_ISL_766796**	EPI1839578|3001288402_N8	Africa / Mali	2020-03-	A/Mali/20077/2020	NIC Lab CVD-MALI	Centers for Disease Control and Prevention	NA
**EPI_ISL_766797**	EPI1839586|3001288389_N8	Africa / Mali	2020-03-	A/Mali/0044/2020	NIC Lab CVD-MALI	Centers for Disease Control and Prevention	NA
**EPI_ISL_766798**	EPI1839594|3001288388_N8	Africa / Mali	2020-03-	A/Mali/0046/2020	NIC Lab CVD-MALI	Centers for Disease Control and Prevention	NA
**EPI_ISL_766799**	EPI1839602|3001288381_N8	Africa / Mali	2020-03-	A/Mali/20018/2020	NIC Lab CVD-MALI	Centers for Disease Control and Prevention	NA
**EPI_ISL_766801**	EPI1839618|3001288382_N8	Africa / Mali	2020-03-	A/Mali/20060/2020	NIC Lab CVD-MALI	Centers for Disease Control and Prevention	NA
**EPI_ISL_766802**	EPI1839626|3001288383_N8	Africa / Mali	2020-03-	A/Mali/20019/2020	NIC Lab CVD-MALI	Centers for Disease Control and Prevention	NA
**EPI_ISL_766803**	EPI1839632|3001288408_N8	Africa / Mali	2020-03-	A/Mali/20084/2020	NIC Lab CVD-MALI	Centers for Disease Control and Prevention	NA
**EPI_ISL_766804**	EPI1839639|3001288404_N8	Africa / Mali	2020-03-	A/Mali/20079/2020	NIC Lab CVD-MALI	Centers for Disease Control and Prevention	NA
**EPI_ISL_766805**	EPI1839647|3001288403_N8	Africa / Mali	2020-03-	A/Mali/20078/2020	NIC Lab CVD-MALI	Centers for Disease Control and Prevention	NA
**EPI_ISL_766806**	EPI1839655|3001288401_N8	Africa / Mali	2020-03-	A/Mali/20057/2020	NIC Lab CVD-MALI	Centers for Disease Control and Prevention	NA
**EPI_ISL_766808**	EPI1839667|3001288385_N8	Africa / Mali	2020-03-	A/Mali/20042/2020	NIC Lab CVD-MALI	Centers for Disease Control and Prevention	NA
**EPI_ISL_766809**	EPI1839674|3001288399_N8	Africa / Mali	2020-03-	A/Mali/20075/2020	NIC Lab CVD-MALI	Centers for Disease Control and Prevention	NA
**EPI_ISL_766810**	EPI1839682|3001288415_N8	Africa / Mali	2020-03-	A/Mali/20092/2020	NIC Lab CVD-MALI	Centers for Disease Control and Prevention	NA
**EPI_ISL_766811**	EPI1839690|3001288412_N8	Africa / Mali	2020-03-	A/Mali/20025/2020	NIC Lab CVD-MALI	Centers for Disease Control and Prevention	NA
**EPI_ISL_766813**	EPI1839706|3001288384_N8	Africa / Mali	2020-03-	A/Mali/20020/2020	NIC Lab CVD-MALI	Centers for Disease Control and Prevention	NA
**EPI_ISL_766816**	EPI1839730|3001288417_N8	Asia / Lao, People's	2020-09-	A/Laos/1741/2020	National Center for Laboratory and Epidemiology	Centers for Disease Control and Prevention	NA
**EPI_ISL_766817**	EPI1839738|3001288428_N8	Asia / Lao, People's	2020-09-	A/Laos/1752/2020	National Center for Laboratory and Epidemiology	Centers for Disease Control and Prevention	NA
**EPI_ISL_766821**	EPI1839770|3001288423_N8	Asia / Lao, People's	2020-09-	A/Laos/1747/2020	National Center for Laboratory and Epidemiology	Centers for Disease Control and Prevention	NA
**EPI_ISL_766822**	EPI1839778|3001288425_N8	Asia / Lao, People's	2020-09-	A/Laos/1749/2020	National Center for Laboratory and Epidemiology	Centers for Disease Control and Prevention	NA
**EPI_ISL_766823**	EPI1839786|3001288418_N8	Asia / Lao, People's	2020-09-	A/Laos/1742/2020	National Center for Laboratory and Epidemiology	Centers for Disease Control and Prevention	NA
**EPI_ISL_766831**	EPI1839849|3001288422_N8	Asia / Lao, People's	2020-09-	A/Laos/1746/2020	National Center for Laboratory and Epidemiology	Centers for Disease Control and Prevention	NA
**EPI_ISL_766834**	EPI1839873|3001288421_N8	Asia / Lao, People's	2020-09-	A/Laos/1745/2020	National Center for Laboratory and Epidemiology	Centers for Disease Control and Prevention	NA
**EPI_ISL_766835**	EPI1839881|3001288429_N8	Asia / Lao, People's	2020-09-	A/Laos/1753/2020	National Center for Laboratory and Epidemiology	Centers for Disease Control and Prevention	NA
**EPI_ISL_766837**	EPI1839897|3001288419_N8	Asia / Lao, People's	2020-09-	A/Laos/1743/2020	National Center for Laboratory and Epidemiology	Centers for Disease Control and Prevention	NA
**EPI_ISL_766838**	EPI1839905|3001288420_N8	Asia / Lao, People's	2020-09-	A/Laos/1744/2020	National Center for Laboratory and Epidemiology	Centers for Disease Control and Prevention	NA
**EPI_ISL_766840**	EPI1839921|3001288426_N8	Asia / Lao, People's	2020-09-	A/Laos/1750/2020	National Center for Laboratory and Epidemiology	Centers for Disease Control and Prevention	NA
**EPI_ISL_766849**	EPI1839988|3001288427_ZZ	Asia / Lao, People's	2020-09-	A/Laos/1751/2020	National Center for Laboratory and Epidemiology	Centers for Disease Control and Prevention	NA
**EPI_ISL_766852**	EPI1840012|3001288452_N8	Asia / Bangladesh	2020-10-	A/Bangladesh/120101	icddr,b International Centre for Diarrhoeal Disease Research,	Centers for Disease Control and Prevention	NA
**EPI_ISL_768591**	EPI1841178|2020-CX0816.4	Asia / China / Yunnan	2020-07-	A/Yunnan-	WHO Chinese National Influenza Center	WHO Chinese National Influenza Center	Li Xiyan, Liu Jia, Dayan Wang, Zeng Xiaoxu,
**EPI_ISL_768592**	EPI1841186|2020-CX0815.4	Asia / China / Yunnan	2020-07-	A/Yunnan-	WHO Chinese National Influenza Center	WHO Chinese National Influenza Center	Li Xiyan, Liu Jia, Dayan Wang, Zeng Xiaoxu,
**EPI_ISL_768593**	EPI1841194|2020-CX0814.4	Asia / China / Yunnan	2020-07-	A/Yunnan-	WHO Chinese National Influenza Center	WHO Chinese National Influenza Center	Li Xiyan, Liu Jia, Dayan Wang, Zeng Xiaoxu,
**EPI_ISL_768594**	EPI1841202|2020-CX0813.4	Asia / China / Yunnan	2020-07-	A/Yunnan-	WHO Chinese National Influenza Center	WHO Chinese National Influenza Center	Li Xiyan, Liu Jia, Dayan Wang, Zeng Xiaoxu,
**EPI_ISL_768595**	EPI1841210|2020-CX0812.4	Asia / China / Yunnan	2020-07-	A/Yunnan-	WHO Chinese National Influenza Center	WHO Chinese National Influenza Center	Li Xiyan, Liu Jia, Dayan Wang, Zeng Xiaoxu,
**EPI_ISL_768596**	EPI1841218|2020-CX0811.4	Asia / China / Yunnan	2020-07-	A/Yunnan-	WHO Chinese National Influenza Center	WHO Chinese National Influenza Center	Li Xiyan, Liu Jia, Dayan Wang, Zeng Xiaoxu,
**EPI_ISL_768597**	EPI1841226|2020-CX0810.4	Asia / China / Yunnan	2020-07-	A/Yunnan-	WHO Chinese National Influenza Center	WHO Chinese National Influenza Center	Li Xiyan, Liu Jia, Dayan Wang, Zeng Xiaoxu,
**EPI_ISL_860634**	EPI1842216|Infl_21-	Europe / Sweden / Stockholms	2021-01-	A/Stockholm/1/2021	Klinisk mikrobiologi, Karolinska Universitetslaboratoriet, Karolinska	Public Health Agency of Sweden	NA
**EPI_ISL_882806**	EPI1842243|3001288466_N8	Asia / Cambodia	2020-09-	A/Cambodia/E092525	WHO Collaborating Centre for Reference and Research on Influenza	Centers for Disease Control and Prevention	NA
**EPI_ISL_882807**	EPI1842251|3001288482_N8	Africa / Togo	2020-10-	A/Togo/771/2020	Institute National D'Hygiene	Centers for Disease Control and Prevention	NA
**EPI_ISL_882808**	EPI1842259|3001288487_N8	Africa / Togo	2020-10-	A/Togo/827/2020	Institute National D'Hygiene	Centers for Disease Control and Prevention	NA
**EPI_ISL_882809**	EPI1842267|3001288584_N8	Asia / Bangladesh	2020-10-	A/Bangladesh/1011/2	Institute of Epidemiology Disease Control and Research (IEDCR) &	Centers for Disease Control and Prevention	NA
**EPI_ISL_882810**	EPI1842275|3001288565_N8	Asia / Bangladesh	2020-09-	A/Bangladesh/1924/2	Institute of Epidemiology Disease Control and Research (IEDCR) &	Centers for Disease Control and Prevention	NA
**EPI_ISL_882811**	EPI1842283|3001288590_N8	Asia / Bangladesh	2020-09-	A/Bangladesh/589/20	Institute of Epidemiology Disease Control and Research (IEDCR) &	Centers for Disease Control and Prevention	NA
**EPI_ISL_882812**	EPI1842291|3001288564_N8	Asia / Bangladesh	2020-09-	A/Bangladesh/1922/2	Institute of Epidemiology Disease Control and Research (IEDCR) &	Centers for Disease Control and Prevention	NA
**EPI_ISL_882813**	EPI1842299|3001288582_N8	Asia / Bangladesh	2020-10-	A/Bangladesh/868/20	Institute of Epidemiology Disease Control and Research (IEDCR) &	Centers for Disease Control and Prevention	NA

**EPI_ISL_882814**	EPI1842307|3001288572_N8	Asia / Bangladesh	2020-09-	A/Bangladesh/1450/2	Institute of Epidemiology Disease Control and Research (IEDCR) &	Centers for Disease Control and Prevention	NA
**EPI_ISL_882815**	EPI1842315|3001288586_N8	Asia / Bangladesh	2020-09-	A/Bangladesh/1005/2	Institute of Epidemiology Disease Control and Research (IEDCR) &	Centers for Disease Control and Prevention	NA
**EPI_ISL_882816**	EPI1842323|3001288575_N8	Asia / Bangladesh	2020-09-	A/Bangladesh/1453/2	Institute of Epidemiology Disease Control and Research (IEDCR) &	Centers for Disease Control and Prevention	NA
**EPI_ISL_882817**	EPI1842331|3001288576_N8	Asia / Bangladesh	2020-09-	A/Bangladesh/1154/2	Institute of Epidemiology Disease Control and Research (IEDCR) &	Centers for Disease Control and Prevention	NA
**EPI_ISL_882818**	EPI1842339|3001288566_N8	Asia / Bangladesh	2020-09-	A/Bangladesh/1926/2	Institute of Epidemiology Disease Control and Research (IEDCR) &	Centers for Disease Control and Prevention	NA
**EPI_ISL_882819**	EPI1842347|3001288568_N8	Asia / Bangladesh	2020-09-	A/Bangladesh/1166/2	Institute of Epidemiology Disease Control and Research (IEDCR) &	Centers for Disease Control and Prevention	NA
**EPI_ISL_882820**	EPI1842355|3001288591_N8	Asia / Bangladesh	2020-10-	A/Bangladesh/1019/2	Institute of Epidemiology Disease Control and Research (IEDCR) &	Centers for Disease Control and Prevention	NA
**EPI_ISL_882821**	EPI1842362|3001288562_N8	Asia / Bangladesh	2020-10-	A/Bangladesh/1937/2	Institute of Epidemiology Disease Control and Research (IEDCR) &	Centers for Disease Control and Prevention	NA
**EPI_ISL_882822**	EPI1842370|3001288574_N8	Asia / Bangladesh	2020-09-	A/Bangladesh/1452/2	Institute of Epidemiology Disease Control and Research (IEDCR) &	Centers for Disease Control and Prevention	NA
**EPI_ISL_882823**	EPI1842376|3001288579_N8	Asia / Bangladesh	2020-09-	A/Bangladesh/1916/2	Institute of Epidemiology Disease Control and Research (IEDCR) &	Centers for Disease Control and Prevention	NA
**EPI_ISL_882824**	EPI1842382|3001288558_N8	Asia / Bangladesh	2020-10-	A/Bangladesh/1478/2	Institute of Epidemiology Disease Control and Research (IEDCR) &	Centers for Disease Control and Prevention	NA
**EPI_ISL_882825**	EPI1842390|3001288589_N8	Asia / Bangladesh	2020-09-	A/Bangladesh/1008/2	Institute of Epidemiology Disease Control and Research (IEDCR) &	Centers for Disease Control and Prevention	NA
**EPI_ISL_882826**	EPI1842398|3001288570_N8	Asia / Bangladesh	2020-10-	A/Bangladesh/1936/2	Institute of Epidemiology Disease Control and Research (IEDCR) &	Centers for Disease Control and Prevention	NA
**EPI_ISL_882827**	EPI1842406|3001288567_N8	Asia / Bangladesh	2020-09-	A/Bangladesh/1813/2	Institute of Epidemiology Disease Control and Research (IEDCR) &	Centers for Disease Control and Prevention	NA
**EPI_ISL_882828**	EPI1842414|3001288578_N8	Asia / Bangladesh	2020-09-	A/Bangladesh/1912/2	Institute of Epidemiology Disease Control and Research (IEDCR) &	Centers for Disease Control and Prevention	NA
**EPI_ISL_882829**	EPI1842419|3001288560_N8	Asia / Bangladesh	2020-10-	A/Bangladesh/1817/2	Institute of Epidemiology Disease Control and Research (IEDCR) &	Centers for Disease Control and Prevention	NA
**EPI_ISL_882830**	EPI1842427|3001288559_N8	Asia / Bangladesh	2020-10-	A/Bangladesh/1928/2	Institute of Epidemiology Disease Control and Research (IEDCR) &	Centers for Disease Control and Prevention	NA
**EPI_ISL_882831**	EPI1842435|3001288561_N8	Asia / Bangladesh	2020-10-	A/Bangladesh/1168/2	Institute of Epidemiology Disease Control and Research (IEDCR) &	Centers for Disease Control and Prevention	NA
**EPI_ISL_882832**	EPI1842443|3001288573_N8	Asia / Bangladesh	2020-09-	A/Bangladesh/1451/2	Institute of Epidemiology Disease Control and Research (IEDCR) &	Centers for Disease Control and Prevention	NA
**EPI_ISL_882833**	EPI1842449|3001288577_N8	Asia / Bangladesh	2020-09-	A/Bangladesh/1157/2	Institute of Epidemiology Disease Control and Research (IEDCR) &	Centers for Disease Control and Prevention	NA
**EPI_ISL_882834**	EPI1842456|3001288571_N8	Asia / Bangladesh	2020-10-	A/Bangladesh/1821/2	Institute of Epidemiology Disease Control and Research (IEDCR) &	Centers for Disease Control and Prevention	NA
**EPI_ISL_882835**	EPI1842464|3001288523_N8	Asia / Bangladesh	2020-09-	A/Bangladesh/3012/2	icddr,b International Centre for Diarrhoeal Disease Research,	Centers for Disease Control and Prevention	NA
**EPI_ISL_882836**	EPI1842472|3001288533_N8	Asia / Bangladesh	2020-09-	A/Bangladesh/320090	icddr,b International Centre for Diarrhoeal Disease Research,	Centers for Disease Control and Prevention	NA
**EPI_ISL_882837**	EPI1842480|3001288510_N8	Asia / Bangladesh	2020-09-	A/Bangladesh/120090	icddr,b International Centre for Diarrhoeal Disease Research,	Centers for Disease Control and Prevention	NA
**EPI_ISL_882838**	EPI1842485|3001288546_N8	Asia / Bangladesh	2020-10-	A/Bangladesh/4006/2	icddr,b International Centre for Diarrhoeal Disease Research,	Centers for Disease Control and Prevention	NA
**EPI_ISL_882839**	EPI1842493|3001288512_N8	Asia / Bangladesh	2020-09-	A/Bangladesh/2006/2	icddr,b International Centre for Diarrhoeal Disease Research,	Centers for Disease Control and Prevention	NA
**EPI_ISL_882840**	EPI1842501|3001288511_N8	Asia / Bangladesh	2020-09-	A/Bangladesh/4004/2	icddr,b International Centre for Diarrhoeal Disease Research,	Centers for Disease Control and Prevention	NA
**EPI_ISL_882841**	EPI1842509|3001288522_N8	Asia / Bangladesh	2020-09-	A/Bangladesh/9005/2	icddr,b International Centre for Diarrhoeal Disease Research,	Centers for Disease Control and Prevention	NA
**EPI_ISL_882842**	EPI1842517|3001288516_N8	Asia / Bangladesh	2020-09-	A/Bangladesh/2014/2	icddr,b International Centre for Diarrhoeal Disease Research,	Centers for Disease Control and Prevention	NA
**EPI_ISL_882843**	EPI1842525|3001288531_N8	Asia / Bangladesh	2020-09-	A/Bangladesh/320090	icddr,b International Centre for Diarrhoeal Disease Research,	Centers for Disease Control and Prevention	NA
**EPI_ISL_882844**	EPI1842533|3001288530_N8	Asia / Bangladesh	2020-09-	A/Bangladesh/3009/2	icddr,b International Centre for Diarrhoeal Disease Research,	Centers for Disease Control and Prevention	NA
**EPI_ISL_882845**	EPI1842541|3001288537_N8	Asia / Bangladesh	2020-09-	A/Bangladesh/320090	icddr,b International Centre for Diarrhoeal Disease Research,	Centers for Disease Control and Prevention	NA
**EPI_ISL_882846**	EPI1842548|3001288524_N8	Asia / Bangladesh	2020-09-	A/Bangladesh/8013/2	icddr,b International Centre for Diarrhoeal Disease Research,	Centers for Disease Control and Prevention	NA
**EPI_ISL_882847**	EPI1842556|3001288535_N8	Asia / Bangladesh	2020-09-	A/Bangladesh/9010/2	icddr,b International Centre for Diarrhoeal Disease Research,	Centers for Disease Control and Prevention	NA
**EPI_ISL_882848**	EPI1842564|3001288532_N8	Asia / Bangladesh	2020-09-	A/Bangladesh/3013/2	icddr,b International Centre for Diarrhoeal Disease Research,	Centers for Disease Control and Prevention	NA
**EPI_ISL_882849**	EPI1842572|3001288534_N8	Asia / Bangladesh	2020-09-	A/Bangladesh/8009/2	icddr,b International Centre for Diarrhoeal Disease Research,	Centers for Disease Control and Prevention	NA
**EPI_ISL_882850**	EPI1842580|3001288519_N8	Asia / Bangladesh	2020-09-	A/Bangladesh/2009/2	icddr,b International Centre for Diarrhoeal Disease Research,	Centers for Disease Control and Prevention	NA
**EPI_ISL_882851**	EPI1842588|3001288525_N8	Asia / Bangladesh	2020-09-	A/Bangladesh/4007/2	icddr,b International Centre for Diarrhoeal Disease Research,	Centers for Disease Control and Prevention	NA
**EPI_ISL_882852**	EPI1842596|3001288508_N8	Asia / Bangladesh	2020-09-	A/Bangladesh/0002/2	icddr,b International Centre for Diarrhoeal Disease Research,	Centers for Disease Control and Prevention	NA
**EPI_ISL_882853**	EPI1842604|3001288513_N8	Asia / Bangladesh	2020-09-	A/Bangladesh/0004/2	icddr,b International Centre for Diarrhoeal Disease Research,	Centers for Disease Control and Prevention	NA
**EPI_ISL_882854**	EPI1842612|3001288540_N8	Asia / Bangladesh	2020-10-	A/Bangladesh/320100	icddr,b International Centre for Diarrhoeal Disease Research,	Centers for Disease Control and Prevention	NA
**EPI_ISL_882855**	EPI1842620|3001288514_N8	Asia / Bangladesh	2020-09-	A/Bangladesh/1013/2	icddr,b International Centre for Diarrhoeal Disease Research,	Centers for Disease Control and Prevention	NA
**EPI_ISL_882856**	EPI1842628|3001288507_N8	Asia / Bangladesh	2020-09-	A/Bangladesh/9002/2	icddr,b International Centre for Diarrhoeal Disease Research,	Centers for Disease Control and Prevention	NA
**EPI_ISL_882857**	EPI1842636|3001288526_N8	Asia / Bangladesh	2020-09-	A/Bangladesh/4013/2	icddr,b International Centre for Diarrhoeal Disease Research,	Centers for Disease Control and Prevention	NA
**EPI_ISL_882858**	EPI1842644|3001288545_N8	Asia / Bangladesh	2020-10-	A/Bangladesh/2010/2	icddr,b International Centre for Diarrhoeal Disease Research,	Centers for Disease Control and Prevention	NA
**EPI_ISL_882859**	EPI1842652|3001288544_N8	Asia / Bangladesh	2020-10-	A/Bangladesh/120101	icddr,b International Centre for Diarrhoeal Disease Research,	Centers for Disease Control and Prevention	NA
**EPI_ISL_882860**	EPI1842660|3001288517_N8	Asia / Bangladesh	2020-09-	A/Bangladesh/320091	icddr,b International Centre for Diarrhoeal Disease Research,	Centers for Disease Control and Prevention	NA
**EPI_ISL_882861**	EPI1842668|3001288536_N8	Asia / Bangladesh	2020-09-	A/Bangladesh/3015/2	icddr,b International Centre for Diarrhoeal Disease Research,	Centers for Disease Control and Prevention	NA
**EPI_ISL_882862**	EPI1842676|3001288520_N8	Asia / Bangladesh	2020-09-	A/Bangladesh/120091	icddr,b International Centre for Diarrhoeal Disease Research,	Centers for Disease Control and Prevention	NA
**EPI_ISL_882863**	EPI1842684|3001288529_N8	Asia / Bangladesh	2020-09-	A/Bangladesh/120090	icddr,b International Centre for Diarrhoeal Disease Research,	Centers for Disease Control and Prevention	NA
**EPI_ISL_882864**	EPI1842692|3001288518_N8	Asia / Bangladesh	2020-09-	A/Bangladesh/2017/2	icddr,b International Centre for Diarrhoeal Disease Research,	Centers for Disease Control and Prevention	NA
**EPI_ISL_882865**	EPI1842700|3001288515_N8	Asia / Bangladesh	2020-09-	A/Bangladesh/120090	icddr,b International Centre for Diarrhoeal Disease Research,	Centers for Disease Control and Prevention	NA
**EPI_ISL_882866**	EPI1842708|3001288527_N8	Asia / Bangladesh	2020-09-	A/Bangladesh/120091	icddr,b International Centre for Diarrhoeal Disease Research,	Centers for Disease Control and Prevention	NA
**EPI_ISL_882867**	EPI1842716|3001288539_N8	Asia / Bangladesh	2020-10-	A/Bangladesh/4012/2	icddr,b International Centre for Diarrhoeal Disease Research,	Centers for Disease Control and Prevention	NA
**EPI_ISL_882868**	EPI1842724|3001288521_N8	Asia / Bangladesh	2020-09-	A/Bangladesh/120090	icddr,b International Centre for Diarrhoeal Disease Research,	Centers for Disease Control and Prevention	NA
**EPI_ISL_882869**	EPI1842732|3001288509_N8	Asia / Bangladesh	2020-09-	A/Bangladesh/1007/2	icddr,b International Centre for Diarrhoeal Disease Research,	Centers for Disease Control and Prevention	NA
**EPI_ISL_882870**	EPI1842740|3001288543_N8	Asia / Bangladesh	2020-10-	A/Bangladesh/1014/2	icddr,b International Centre for Diarrhoeal Disease Research,	Centers for Disease Control and Prevention	NA
**EPI_ISL_882871**	EPI1842748|3001288541_N8	Asia / Bangladesh	2020-10-	A/Bangladesh/320100	icddr,b International Centre for Diarrhoeal Disease Research,	Centers for Disease Control and Prevention	NA
**EPI_ISL_944641**	EPI1843595|S10041710.4	Asia / Cambodia / Khett Preah	2020-10-	A/Cambodia/e100925	Institute Pasteur du Cambodia	WHO Collaborating Centre for Reference	Deng,Y-M; Iannello,P; Lau,H; Spirason,N;
**EPI_ISL_944642**	EPI1843598|S10041711.4	Asia / Cambodia / Khett Prey	2020-09-	A/Cambodia/e092525	Institute Pasteur du Cambodia	WHO Collaborating Centre for Reference	Deng,Y-M; Iannello,P; Lau,H; Spirason,N;
**EPI_ISL_956319**	EPI1843602|A/AbuDhabi/01/2	Asia / United Arab Emirates /	2021-01-	A/AbuDhabi/01/2021	Shaikh Khalifa Medical City	Sheikh Khalifa Medical City (SKMC)	Younan, Mary
**EPI_ISL_956338**	EPI1847030|A/AbuDhabi/2375	Asia / United Arab Emirates /	2021-01-	A/AbuDhabi/2375/202	Sheikh Khalifa Medical City (SKMC)	Sheikh Khalifa Medical City (SKMC)	Younan, Mary
**EPI_ISL_959655**	EPI1843691|55-a-cote_divoire-	Africa / Cote d'Ivoire	2020-11-	A/Cote	Pasteur Institut of Côte d'Ivoire	Crick Worldwide Influenza Centre	NA
**EPI_ISL_959656**	EPI1843693|93-a-cote_divoire-	Africa / Cote d'Ivoire	2020-11-	A/Cote	Pasteur Institut of Côte d'Ivoire	Crick Worldwide Influenza Centre	NA
**EPI_ISL_959657**	EPI1843695|49-a-ghana-112-	Africa / Ghana	2020-11-	A/Ghana/112/2020	University of Ghana	Crick Worldwide Influenza Centre	NA
**EPI_ISL_959658**	EPI1843696|46-	Africa / Ghana	2020-11-	A/Ghana/2170/2020	University of Ghana	Crick Worldwide Influenza Centre	NA
**EPI_ISL_959659**	EPI1843698|45-	Africa / Ghana	2020-11-	A/Ghana/2180/2020	University of Ghana	Crick Worldwide Influenza Centre	NA
**EPI_ISL_965035**	EPI1843859|3030724502_ZZ	North America / United States /	2020-11-	A/Michigan/173/2020	Michigan Department of Community Health	Centers for Disease Control and Prevention	NA

**EPI_ISL_965036**	EPI1843864|3001288585_N8	Asia / Bangladesh	2020-10-	A/Bangladesh/630/20	Institute of Epidemiology Disease Control and Research (IEDCR) &	Centers for Disease Control and Prevention	NA
**EPI_ISL_965037**	EPI1843873|3001288613_N8	Asia / India	2020-08-	A/India/PUN-	National Institute of Virology	Centers for Disease Control and Prevention	NA
**EPI_ISL_965038**	EPI1843878|3001288542_N8	Asia / Bangladesh	2020-10-	A/Bangladesh/9012/2	icddr,b International Centre for Diarrhoeal Disease Research,	Centers for Disease Control and Prevention	NA
**EPI_ISL_984696**	EPI1845830|16-	Africa / Cote d'Ivoire	2020-10-	A/Cote	Pasteur Institut of Côte d'Ivoire	Crick Worldwide Influenza Centre	NA
**EPI_ISL_984697**	EPI1845832|1-	Africa / Cote d'Ivoire	2020-11-	A/Cote	Pasteur Institut of Côte d'Ivoire	Crick Worldwide Influenza Centre	NA
**EPI_ISL_984699**	EPI1845836|24-	Africa / Cote d'Ivoire	2020-10-	A/Cote	Pasteur Institut of Côte d'Ivoire	Crick Worldwide Influenza Centre	NA
**EPI_ISL_984700**	EPI1845838|30-	Africa / Cote d'Ivoire	2020-10-	A/Cote	Pasteur Institut of Côte d'Ivoire	Crick Worldwide Influenza Centre	NA
**EPI_ISL_984701**	EPI1845840|31-	Africa / Cote d'Ivoire	2020-09-	A/Cote	Pasteur Institut of Côte d'Ivoire	Crick Worldwide Influenza Centre	NA
**EPI_ISL_984703**	EPI1845843|45-	Europe / France	2020-10-	A/Grenoble/2789/202	CNR Virus des Infections Respiratoires - France SUD	Crick Worldwide Influenza Centre	NA
**EPI_ISL_984705**	EPI1845847|5-	Africa / Cote d'Ivoire	2020-11-	A/Cote	Pasteur Institut of Côte d'Ivoire	Crick Worldwide Influenza Centre	NA
**EPI_ISL_984706**	EPI1845848|69-	Asia / Oman	2020-10-	A/Salalah/520266545/	Central Public Health Laboratory, Ministry of Health	Crick Worldwide Influenza Centre	NA
**EPI_ISL_984709**	EPI1845854|80-	Africa / Ghana	2020-11-	A/Ghana/2287/2020	University of Ghana	Crick Worldwide Influenza Centre	NA
**EPI_ISL_984710**	EPI1845856|81-	Africa / Cote d'Ivoire	2020-11-	A/Cote	Pasteur Institut of Côte d'Ivoire	Crick Worldwide Influenza Centre	NA
**EPI_ISL_984711**	EPI1845858|82-	Africa / Cote d'Ivoire	2020-11-	A/Cote	Pasteur Institut of Côte d'Ivoire	Crick Worldwide Influenza Centre	NA
**EPI_ISL_984712**	EPI1845860|83-	Africa / Cote d'Ivoire	2020-11-	A/Cote	Pasteur Institut of Côte d'Ivoire	Crick Worldwide Influenza Centre	NA
**EPI_ISL_984713**	EPI1845862|84-	Africa / Cote d'Ivoire	2020-11-	A/Cote	Pasteur Institut of Côte d'Ivoire	Crick Worldwide Influenza Centre	NA
**EPI_ISL_984714**	EPI1845864|85-	Africa / Cote d'Ivoire	2020-10-	A/Cote	Pasteur Institut of Côte d'Ivoire	Crick Worldwide Influenza Centre	NA
**EPI_ISL_985212**	EPI1846156|S10041963.4	Asia / Malaysia	2020-03-	A/Malaysia/RP2216/20	Institut Penyelidikan Perubatan	WHO Collaborating Centre for Reference	Deng,Y-M; Iannello,P; Lau,H; Spirason,N;
**EPI_ISL_985213**	EPI1846159|S10041964.4	Asia / Malaysia	2020-03-	A/Malaysia/RP2204/20	Institut Penyelidikan Perubatan	WHO Collaborating Centre for Reference	Deng,Y-M; Iannello,P; Lau,H; Spirason,N;
**EPI_ISL_985214**	EPI1846162|S10041965.4	Asia / Malaysia	2020-03-	A/Malaysia/RP2219/20	Institut Penyelidikan Perubatan	WHO Collaborating Centre for Reference	Deng,Y-M; Iannello,P; Lau,H; Spirason,N;
**EPI_ISL_985215**	EPI1846165|S10041966.4	Asia / Malaysia	2020-03-	A/Malaysia/RP2207/20	Institut Penyelidikan Perubatan	WHO Collaborating Centre for Reference	Deng,Y-M; Iannello,P; Lau,H; Spirason,N;
**EPI_ISL_985216**	EPI1846168|S10041967.4	Asia / Malaysia	2020-03-	A/Malaysia/RP2227/20	Institut Penyelidikan Perubatan	WHO Collaborating Centre for Reference	Deng,Y-M; Iannello,P; Lau,H; Spirason,N;
**EPI_ISL_985217**	EPI1846171|S10041968.4	Asia / Malaysia	2020-03-	A/Malaysia/RP2217/20	Institut Penyelidikan Perubatan	WHO Collaborating Centre for Reference	Deng,Y-M; Iannello,P; Lau,H; Spirason,N;
**EPI_ISL_985218**	EPI1846174|S10041969.4	Asia / Malaysia	2020-03-	A/Malaysia/RP2215/20	Institut Penyelidikan Perubatan	WHO Collaborating Centre for Reference	Deng,Y-M; Iannello,P; Lau,H; Spirason,N;
**EPI_ISL_985219**	EPI1846177|S10041970.4	Asia / Malaysia	2020-03-	A/Malaysia/RP2223/20	Institut Penyelidikan Perubatan	WHO Collaborating Centre for Reference	Deng,Y-M; Iannello,P; Lau,H; Spirason,N;
**EPI_ISL_996015**	EPI1846324|65-	Africa / Cote d'Ivoire	2020-11-	A/Cote	Pasteur Institut of Côte d'Ivoire	Crick Worldwide Influenza Centre	NA
**EPI_ISL_996020**	EPI1846334|15-	Africa / Senegal	2020-11-	A/Dakar/01/2020	Institut Pasteur de Dakar	Crick Worldwide Influenza Centre	NA
**EPI_ISL_996021**	EPI1846336|16-	Africa / Senegal	2020-10-	A/Dakar/02/2020	Institut Pasteur de Dakar	Crick Worldwide Influenza Centre	NA
**EPI_ISL_996022**	EPI1846338|17-	Africa / Senegal	2020-10-	A/Dakar/03/2020	Institut Pasteur de Dakar	Crick Worldwide Influenza Centre	NA
**EPI_ISL_996023**	EPI1846340|3-	Africa / Senegal	2020-10-	A/Dakar/10B/2020	Institut Pasteur de Dakar	Crick Worldwide Influenza Centre	NA
